# Change sign detection with differential MDL change statistics and its applications to COVID-19 pandemic analysis

**DOI:** 10.1038/s41598-021-98781-4

**Published:** 2021-10-05

**Authors:** Kenji Yamanishi, Linchuan Xu, Ryo Yuki, Shintaro Fukushima, Chuan-hao Lin

**Affiliations:** 1 Graduate School of Information Science and Technology, The University of Tokyo, Tokyo, 113-8656 Japan; 2 Department of Computing, The Hong Kong Polytechnic University, Hung Hom, Kowloon, Hong Kong

**Keywords:** Applied mathematics, Information technology, Statistics

## Abstract

We are concerned with the issue of detecting changes and their signs from a data stream. For example, when given time series of COVID-19 cases in a region, we may raise early warning signals of an epidemic by detecting signs of changes in the data. We propose a novel methodology to address this issue. The key idea is to employ a new information-theoretic notion, which we call the *differential minimum description length change statistics* (D-MDL), for measuring the scores of change sign. We first give a fundamental theory for D-MDL. We then demonstrate its effectiveness using synthetic datasets. We apply it to detecting early warning signals of the COVID-19 epidemic using time series of the cases for individual countries. We empirically demonstrate that D-MDL is able to raise early warning signals of events such as significant increase/decrease of cases. Remarkably, for about $64\%$ of the events of significant increase of cases in studied countries, our method can detect warning signals as early as nearly six days on average before the events, buying considerably long time for making responses. We further relate the warning signals to the dynamics of the basic reproduction number *R*0 and the timing of social distancing. The results show that our method is a promising approach to the epidemic analysis from a data science viewpoint.

## Introduction

### Motivation

We address the issue of detecting changes and their signs in a data stream. For example, when given time series of the number of COVID-19 cases in a region, we may expect to warn the beginning of an epidemic by detecting changes and their signs.

Although change detection^1–3^ is a classical issue, it has remained open how signs of changes can be found. In principle the degree of change at a given time point has been evaluated in terms of the discrepancy measure (e.g.. the Kullback–Leibler (KL) divergence) between probability distributions of data before and after that time point^1,4^. It is reasonable to think that the differentials of the KL divergence may be related to signs of change. This is because the first differential of the KL divergence is a velocity of change while its second differential is an acceleration of change.

The problem is here that in real cases, the KL-divergence and its differentials cannot be exactly calculated since the true distribution is unknown in advance. A question lies in how we can estimate the discrepancy measure and their differentials from data when the parameter values are unknown.

The purpose of this paper is to answer the above question from an information-theoretic viewpoint based on the *minimum description length* (MDL) principle^5^ (see also studies^6,7^ for its recent advances). The MDL principle gives a strategy for evaluating the goodness of a probabilistic model in terms of codelength required for encoding the data where a shorter codelength indicates a better model. We apply this principle to change detection where a shorter codelength indicates a more significant change. Along this idea, we introduce the notion called the *differential MDL change statistics* (D-MDL) for the measure of change signs. We theoretically and empirically justify this notion, and then apply it to the COVID-19 pandemic analysis using open datasets.

### Related work

There are plenty of work on change detection^1–4,8–11^. In many of them, the degree of change has been related to the discrepancy measure for two distributions before and after a time point, such as likelihood ratio, KL-divergence. However, there is no work on relating the differential information such as the velocity of the change to change sign detection.

Most of previous studies in change detection are concerned with detecting *abrupt changes*^3^. In the scenario of concept drift^12^, the issues of detecting various types of changes, including *incremental changes* and *gradual changes*, have been addressed. How to find signs of changes has been addressed in the scenarios of volatility shift detection^13^, gradual change detection^14^ and clustering change detection^15–17^. However, the notion of differential information has never been related to change sign detection.

The MDL change statistics has been proposed as a test statistics in the hypothesis testing for change detection^14,18^. It is defined as the difference between the total codelength required for encoding data for the non-change case and that for the change case at a specific time point *t*. A number of data compression-based change statistics similar to it have also been proposed in data mining^19–21^. However, any differential variation of the compression-based change statistics has never been proposed.

### Significance of this paper

The significance of this paper is summarized as follows:

1. *Proposal of D-MDL and its use for change sign detection.* We introduce a novel notion of D-MDL as an approximation of KL-divergence of change and its differentials. We then propose practical algorithms for on-line detection of change signs on the basis of D-MDL.
2. *Theoretical and empirical justification of D-MDL.* We theoretically justify D-MDL in the hypothesis testing of change detection. We consider the hypothesis tests which are equivalent with D-MDL scoring. We derive upper bounds on the error probabilities for these tests to show that they converge exponentially to zero as sample size increases. The bounds on the error probabilities are used to determine a threshold for raising an alarm with D-MDL. We also empirically justify D-MDL using synthetic datasets. We demonstrate that D-MDL outperforms existing change detection methods in terms of AUC for detecting the starting point of a gradual change.
3. *Applications to COVID-19 pandemic analysis.* On the basis of the theoretical and empirical advantages of D-MDL, we apply it to the COVID-19 pandemic analysis. We are mainly concerned with how early we are able to detect signs of outbreaks or the contraction of the epidemic for individual countries. The results showed that for about $64\%$ of outbreaks in studied countries, our method can detect signs as early as about 6 days on average before the outbreaks. Considering the rapid spread, 6 days can earn us considerably long time for making responses, e.g., implementing control measures^22–24^. The earned time is especially precious in the presence of a considerably long period of the incubation of the COVID-19^25–27^. Moreover, we analyze relations between the change detection results and social distancing events. One of findings is that for individual countries, an average of about four changes/change signs detected before the implementation of social distancing correlates a significant decline from the peak of daily new cases by the end of April 2020.

The change analysis is a pure data science methodology, which detects changes only using statistical models without using differential equations about the time evolution. Meanwhile, SIR (Susceptible Infected Recovered) model^28^ is a typical simulation method which predicts the time evolution of infected population with physics model-based differential equations. Although the fitness of the SIR model or its variants to COVID-19 data was argued^29,30^, the complicated situation of COVID-19 due to virus mutations^31–33^, international interactions, highly variable responses from authorities^34^, environmental effects^35,36^ etc. does not necessarily make any simulation model perfect. Therefore, the basic reproduction number *R*0^37^ (a term in epidemiology, representing the average number of people who will contract a contagious disease from one person with that disease) estimated from the SIR model may not be precise. We empirically demonstrate that as a byproduct, the dynamics of *R*0 can be monitored by our methodology which only requires the information of daily new cases. The data science approach then may form a complementary relation with the simulation approach and gives new insights into epidemic analysis. The effect of social distancing in Germany has been evaluated using the framework of change point analysis together with SIR model^38^. However, there is no work on machine learning approaches to detecting signs of outbreak for COVID-19.

The software for the experiments is available at https://github.com/IbarakikenYukishi/differential-mdl-change-statistics. An online detection system is available at https://ibarakikenyukishi.github.io/d-mdl-html/index.html

The rest of this paper is organized as follows: “Methods” introduces D-MDL and gives a theory of its use in the context of change sign detection. “Result I: experiments with synthetic data” gives empirical justification of D-MDL using synthetic datasets. “Result II: applications to COVID-19 pandemic analysis” gives applications of D-MDL to the COVID-19 pandemic analysis. “Conclusion” gives concluding remarks.

## Methods

### Definitions of changes and their signs

Let ${{\mathcal {X}}}$ be a domain, which is either discrete or continuous. Hereafter we assume that ${{\mathcal {X}}}$ is discrete without loss of generality. For a random variable ${{\varvec{x}}}\in {{\mathcal {X}}}$, let $p({{\varvec{x}}};\theta )=p_{_{\theta }}({{\varvec{x}}})$ be the probability mass function (or the probability density function in the continuous case) specified by a parameter $\theta$. Supposing that $\theta$ changes over time. In the case when $\theta$ gradually changes over time, we define the *signs of change* as the starting point of that change.

Let us consider the discrete time *t*. Let $\theta _{t}$ be the parameter value of $\theta$ at time *t*. Let *D*(*p*||*q*) denote the Kullback-Leibler (KL) divergence between two probability mass functions *p* and *q*:

$$\begin{aligned} D(p||q)=\sum _{{{\varvec{x}}}}p({{\varvec{x}}})\log \frac{p({{\varvec{x}}})}{q({{\varvec{x}}})}. \end{aligned}$$

We define the 0th, 1st, 2nd change degrees at time *t* as

$$\begin{aligned}&\Phi _{t}^{(0)}\buildrel {\rm def} \over =D(p_{_{\theta _{t}}}||p_{_{\theta _{t-1}}}),\\&\Phi _{t}^{(1)}\buildrel {\rm def }\over =\Phi _{t+1}^{(0)}-\Phi _{t}^{(0)} =D(p_{_{\theta _{t+1}}}||p_{_{\theta _{t}}})-D(p_{_{\theta _{t}}}||p_{_{\theta _{t-1}}}),\\&\Phi _{t}^{(2)}\mathop{=}^{\!\ \rm{def}}\Phi _{t}^{(1)}-\Phi _{t-1}^{(1)}=D(p_{_{\theta _{t+1}}}||p_{_{\theta _{t}}})-2D(p_{_{\theta _{t}}}||p_{_{\theta _{t-1}}})+D(p_{_{\theta _{t-1}}}||p_{_{\theta _{t-2}}}). \end{aligned}$$

When the parameter sequence $\{\theta _{t}: t\in {{\mathbb {Z}}}\}$ is known, we can define the degree of changes at any given time point. We can think of $\Phi _{t}^{(0)}$ as the degree of change of the parameter value itself at time *t*. We can think of $\Phi _{t}^{(1)}, \Phi _{t}^{(2)}$ as the *velocity of change* and the *acceleration of change* of the parameter at time *t*, respectively. All of them quantify the signs of change. However, the parameter values are not known in advance for general cases. The problem is how we can define the degree of changes for such cases.

### Differential MDL change statistics

In the case where the true parameter values are unknown, the *MDL change statistics* has been proposed to measure the change degree^14,18^ from a given data sequence. Below we denote $x_{a},\dots , x_{b}=x_{a}^{b}$. In the case of $a=1$, we may drop off *a* and write it as $x^{b}$.

When the parameter $\theta$ is unknown, we may estimate it as ${\hat{\theta }}$ using the maximum likelihood estimation method from a given sequence $x^{n}$. I.e., ${\hat{\theta }}= \text {argmax} _{\theta }p(x^{n};\theta ).$ Note that the maximum likelihood function $p(x^{n};{\hat{\theta }})$ does not form a probability distribution of $x^{n}$ because $\sum _{x^{n}}p(x^{n};{\hat{\theta }})>1$. Thus we construct a *normalized maximum likelihood* (NML) distribution^40^ by

$$\begin{aligned} p_{_{\rm{NML}}}(x^{n})\mathop{=}^{\! \rm{def}}\frac{\max _{\theta }p(x^{n};\theta )}{\sum _{y^{n}}\max _{\theta }p(y^{n};\theta )}=\frac{\max _{\theta }p(x^{n};\theta )}{C_{n}} \end{aligned}$$

and consider the logarithmic loss for $x^{n}$ relative to this distribution by

1 $$\begin{aligned} L_{\rm{NML}}(x^{n})\mathop{=}^{ \rm{def}}-\log p_{_{\rm{NML}}}(x^{n}), \end{aligned}$$

which we call the *NML codelength*, where log means the natural logarithm and $C_{n}$ is called the *parametric complexity* defined as

2 $$\begin{aligned} C_{n}\mathop{=}^{ \rm{def}}\sum _{x^{n}}\max _{\theta }p(x^{n};\theta ). \end{aligned}$$

It is known^39^ that Eq. (1) is the optimal codelength that achieves the Shtarkov’s minimax regret in the case where the parameter value is unknown. It is known^40^ that under some regularity condition for the model class, $C_{n}$ is asymptotically expanded as follows:

3 $$\begin{aligned} C_{n}=\frac{d}{2}\log \frac{n}{2\pi }+\log \int \sqrt{|I(\theta )|}d\theta +o(1), \end{aligned}$$

where $I(\theta )$ is the Fisher information matrix defined by $I(\theta )=\lim _{n\rightarrow \infty }(1/n)E_{\theta }[-\partial ^{2}\log p(X^{n}; \theta )/\partial \theta \partial \theta ^{\top }]$, *d* is the dimensionality of $\theta$, and $\lim _{n\rightarrow \infty }o(1)=0$.

According to the study^14^, the *MDL change statistics* at time point *t* is defined as follows:

4 $$\begin{aligned} \Psi _{t}^{(0)}&\mathop{=}^{ \rm{def}}&\frac{1}{n}\{L_{_{\rm{NML}}}(x^{n}_{1})- (L_{_{\rm{NML}}}(x^{t}_{1})+L_{_{\rm{NML}}}(x_{t+1}^{n})). \end{aligned}$$

The MDL change statistics is the difference between that the NML codelength of a given data sequence for non-change and that for change at time *t*. It is a generalization of the likelihood ratio test^1,41^.

Therefore, by extending the change degrees $\Phi _{t}^{(0)}, \Phi _{t}^{(1)}, \Phi _{t}^{(2)},\dots$ to the cases where the true parameters are unknown, we may consider the following statistics:

5 $$\begin{aligned} \Psi _{t}^{(1)}&\mathop{=}^{ \rm{def}}&\Psi _{t+1}^{(0)}-\Psi _{t}^{(0)}, \end{aligned}$$

6 $$\begin{aligned} \Psi _{t}^{(2)}&\mathop{=}^{\rm{def}}&\Psi _{t}^{(1)}-\Psi _{t-1}^{(1)}=\Psi _{t+1}^{(0)}-2\Psi _{t}^{(0)}+\Psi _{t-1}^{(0)}, \nonumber \\&\cdots \end{aligned}$$

$\Psi _{t}^{(\alpha )}$ corresponds to $\Phi _{t}^{(\alpha )}$. We call $\Psi _{t}^{(\alpha )}$ the $\alpha$th *differential MDL change statistics*, which we abbreviate as the $\alpha$th D-MDL ($\alpha =0,1,2,\dots )$. The 0th D-MDL is the original MDL change statistics as in the study^14^.

For example, let us consider the uni-variate Gaussian distribution:

7 $$\begin{aligned} p(x; \theta )=\frac{1}{\sqrt{2\pi }\sigma }\exp \left( -\frac{(x-\mu )^{2}}{2\sigma ^{2}}\right) , \end{aligned}$$

where $x\in {{\mathbb {R}}}$ and $\theta =(\mu , \sigma )$. We assume $|\mu |< \mu _{\max }$ and $\sigma _{\min }<\sigma <\sigma _{\max }$ where $\mu _{\max }<\infty$, $0<\sigma _{\min }, \sigma _{\max }<\infty$ are hyper parameters. The 0th D-MDL at time *t* is calculated as

8 $$\begin{aligned} \Psi _t^{(0)}=\frac{1}{n} \log \frac{{\hat{\sigma }}_{0}^{n}}{{\hat{\sigma }}_{1}^{t} {\hat{\sigma }}_{2}^{n-t}} + \frac{1}{n}\log \frac{C_{n}}{C_{t}C_{n-t}}, \end{aligned}$$

where ${\hat{\sigma }}_{0}, {\hat{\sigma }}_{1}$ and ${\hat{\sigma }}_{2}$ denote the maximum likelihood (ML) estimators of $\sigma$ calculated for $x_{1}^{n}, x_{1}^{t}$ and $x_{t+1}^{n}$, respectively. $C_n$ is the parametric complexity, which is calculated according to the study^14^, as

$$\begin{aligned} \log C_n= \frac{1}{2} \log \frac{16|\mu _{\rm {max}}|}{\pi \sigma ^2_{\rm {min}}} +\frac{n}{2}\log \frac{n}{2\mathrm {e}} -\log \Gamma \left( \frac{n-1}{2}\right) . \end{aligned}$$

The 1st and 2nd D-MDL are calculated according to Eqs. (5) and (6) on the basis of Eq. (8).

### Hypothesis testing for change detection

#### The 0th D-MDL test

We give rationale of D-MDL using the framework of hypothesis testing for change detection. First suppose that a change point exists at *t* or not. Let us consider the following hypothesis testing framework: The null hypothesis $H_{0}$ is that there is no change point while the alternative hypothesis $H_{1}$ is that *t* is an only change point.

$$\begin{aligned} {\left\{ \begin{array}{ll} H_{0}: &{}x^{n}_{1}\sim p(X^{n};\theta _{0}),\\ H_{1}: &{}x_{1}^{t}\sim p(X^{t};\theta _{1}),\ \ x_{t+1}^{n}\sim p(X^{n-t};\theta _{2}), \end{array}\right. } \end{aligned}$$

where $\theta _{0},\theta _{1},\theta _{2}\ (\theta _{1}\ne \theta _{2})$ are all unknown.

With the MDL principle, the test statistics is given as follows: For an accuracy parameter $\epsilon >0$,

9 $$\begin{aligned} h_{0}(x^{n}; t, \epsilon )\mathop{=}^{ \rm{def}} \frac{1}{n}\{L_{_{\rm{NML}}}(x^{n}_{1})-(L_{_{\rm{NML}}}(x^{t}_{1})+L_{_{\rm{NML}}}(x_{t+1}^{n}))\}-\epsilon =\Psi _{t}^{(0)}-\epsilon , \end{aligned}$$

where $\Psi _{t}^{(0)}$ is the 0th D-MDL as in equation (4). $H_{1}$ is accepted if $h_{0}(x^{n}; t, \epsilon )>0$, otherwise $H_{0}$ is accepted. We call this test the *0th D-MDL test*.

We define *Type I error probability* as the probability that the test accepts $H_1$ although $H_{0}$ is true (false alarm rate) while *Type II error probability* as the one that the test accepts $H_{0}$ although $H_{1}$ is true (overlooking rate). The following theorem justifies the use of the 0th D-MDL in change detection.

##### Theorem 2.1

^14^ Type I and II error probabilities for the 0th D-MDL test are upper bounded as follows:

10 $$\begin{aligned} \mathrm{Type\ I\ error\ prob.}< & {} \exp \left[ -n\left( \epsilon -\frac{\log C_{n}}{n}\right) \right] , \end{aligned}$$

11 $$\begin{aligned} \mathrm{Type\ II\ error\ prob.}\le & {} \exp \left[ -n\left( d(p_{_{\rm{NML}}},p_{_{\theta _{1}*\theta _{2}}})-\frac{\log C_{t}C_{n-t}}{2n}- \frac{\epsilon }{2} \right) \right] , \end{aligned}$$

where $C_{n}$ is the parametric complexity as in Eq. (2) and

12 $$\begin{aligned} &d(p,q)\mathop{=}^{\rm{def}}-\frac{1}{n}\log \left( \sum_{x^{n}}(p(x^{n})q(x^{n}))^{\frac{1}{2}}\right) ,\nonumber \\&p_{_{\rm{NML}}}(x^{n})=\frac{\max _{\theta }p(x^{n};\theta )}{\sum _{y^{n}}\max _{\theta }p(y^{n};\theta )},\ \ p_{_{\theta _{1}*\theta _{2}}}(x^{n})=p(x^{t}_{1};\theta _{1})p(x_{t+1}^{n};\theta _{2}). \end{aligned}$$

*d*(*p*, *q*) in Eq. (12) is the Bhattcharyya distance between *p* and *q*.

This theorem shows that Type I and II error probabilities in Eqs. (10) and (11) converge to zero exponentially in *n* as *n* increases for some appropriate $\epsilon$ when $d(p_{_{\rm{NML}}},p_{_{\theta _{1}*\theta _{2}}})$ is large. We see that the error exponents are governed by the parametric complexity (2) of the model class. In this sense the 0th MDL test is effective in change point detection.

#### The 1st D-MDL test

Next we give a hypothesis testing setting equivalent with the 1st D-MDL scoring. We consider the situation where a change point exists at time either *t* or $t+1$. Let us consider the following hypotheses: The null hypothesis $H_{0}$ is that the change point is *t* while the alternative one $H_{1}$ is that it is $t+1$.

$$\begin{aligned} {\left\{ \begin{array}{ll} H_{0}: &{}x^{t}_{1}\sim p(X^{t};\theta _{0}),\ \ x^{n}_{t+1}\sim p(X^{n-t};\theta _{1}), \\ H_{1}: &{}x_{1}^{t+1}\sim p(X^{t+1};\theta _{2}),\ \ x_{t+2}^{n}\sim p(X^{n-t-1};\theta _{3}), \end{array}\right. } \end{aligned}$$

where $\theta _{0},\theta _{1},\theta _{2},\theta _{3}\ (\theta _{0}\ne \theta _{1},\ \theta _{2}\ne \theta _{3})$ are all unknown.

We consider the following test statistics: For an accuracy parameter $\epsilon >0$,

13 $$\begin{aligned} h_{1}(x^{n}; t, \epsilon )\mathop{=}^{ \rm{def}}\frac{1}{n}\left\{ \left( L_{_{\rm{NML}}}(x^{t}_{1})+L_{_{\rm{NML}}}(x_{t+1}^{n})\right) -\left( L_{_{\rm{NML}}}(x_{1}^{t+1})+L_{_{\rm{NML}}}(x_{t+2}^{n})\right) \right\} -\epsilon , \end{aligned}$$

which compares the NML codelength for $H_{0}$ with that for $H_{1}.$ We accept $H_{1}$ if $h_{1}(x^{n}; t, \epsilon )>0$, otherwise we accept $H_{0}$. We call this test the *1st D-MDL test*. We easily see

14 $$\begin{aligned} h_{1}(x^{n}; t, \epsilon )=\Psi _{t}^{(1)}-\epsilon =\Psi _{t+1}^{(0)}-\Psi _{t}^{(0)}-\epsilon , \end{aligned}$$

where $\Psi _{t}^{(1)}$ is the 1st D-MDL. This implies that the 1st D-MDL test is equivalent with testing whether the 1st D-MDL is larger than $\epsilon$ or not. Hence this test is also equivalent with comparison of the degree of change at time $t+1$ and that at time *t*. Intuitively, if the degree of change increases significantly as time goes by, then $H_{1}$ is accepted. Thus the basic performance of discrimination of the 1st D-MDL can be reduced to that of the 1st D-MDL test.

The following theorem shows the basic property of the 1st D-MDL test.

##### Theorem 2.2

Type I and II error probabilities for the 1st D-MDL test are upper bounded as follows:

15 $$\begin{aligned} \mathrm{Type\ I\ error\ prob.}< & {} \exp \left[ -n\left( \epsilon -\frac{\log C_{t}C_{n-t}}{n}\right) \right] , \end{aligned}$$

16 $$\begin{aligned} \mathrm{Type\ II\ error\ prob.}\le & {} \exp \left[ -n\left( d(p_{_{\rm{NML}}(t)},p_{_{\theta _{2}*\theta _{3}}})-\frac{\log C_{t+1}C_{n-t-1}}{2n}- \frac{\epsilon }{2} \right) \right] , \end{aligned}$$

where $C_{n}$ is the parametric complexity as in Eq. (2), *d* is the Bhattacharyya distance as in Eq. (12) and

$$\begin{aligned} p_{_{\rm{NML}}(t)}(x^{n})= & {} \frac{\max _{\theta }p(x^{t}_{1};\theta )}{\sum _{y^{t}_{1}}\max _{\theta }p(y^{t}_{1};\theta )}\cdot \frac{\max _{\theta }p(x^{n}_{t+1};\theta )}{\sum _{y^{n}_{t+1}}\max _{\theta }p(y^{n}_{t+1};\theta )},\\ p_{_{\theta _{2}*\theta _{3}}}(x^{n})= & {} p(x^{t+1}_{1};\theta _{2})p(x_{t+2}^{n};\theta _{3}). \end{aligned}$$

(The proof is in Sec. 1 of the supplementary information.)

This theorem shows that for some appropriate $\epsilon$, Type I and II error probabilities in Eqs. (15) and (16) converge to zero exponentially in *n* as *n* increases where the error exponents are related to the parametric complexities for the hypotheses as well as the Bhattacharyya distance between the null and alternative hypotheses. In this sense the 1st MDL test is effective. Type I error probability in Eq. (15) will be used for determining a threshold of the alarm.

#### The 2nd D-MDL test

Next we consider a hypothesis testing setting equivalent with the 2nd D-MDL scoring. Suppose that change points exist either at time *t* or at $t-1$ and $t+1$.

$$\begin{aligned} {\left\{ \begin{array}{ll} H_{0}: &{}x^{t}_{1}\sim p(X^{t};\theta _{0}),\ \ x_{t+1}^{n}\sim p(X^{n-t};\theta _{1}),\\ H_{1}: &{}x^{t-1}_{1}\sim p(X^{t-1};\theta _{2}),\ \ x_{t}x_{t+1}\sim p(X^{2};\theta _{3}), \ x_{t+2}^{n}\sim p(X^{n-t-1};\theta _{4}). \end{array}\right. } \end{aligned}$$

where $\theta _{0},\theta _{1},\theta _{2},\theta _{3},\theta _{4},\ (\theta _{0}\ne \theta _{1}, \theta _{2}\ne \theta _{3}\ne \theta _{4})$ are all unknown. $H_{0}$ is the hypothesis that a change happens at time *t* while $H_{1}$ is the hypothesis that two changes happen at time $t-1$ and *t*. In $H_{0}$, *t* is a single change point while in $H_{1},$ *t* is a transition point between two close change points. Thus this hypothesis testing evaluates whether time *t* is a change point or a transition point of close changes.

The test statistics is: For an accuracy parameter $\epsilon >0$,

17 $$\begin{aligned} h_{2}(x^{n}; t, \epsilon )&\mathop{=}^{ \rm{def}}&\frac{1}{n}\left\{ \left( L_{_{\rm{NML}}}(x_{1}^{t})+L_{_{\rm{NML}}}(x_{t+1}^{n}) \right) -\left( L_{_{\rm{NML}}}(x_{1}^{t-1})+L_{_{\rm{NML}}}(x_{t}x_{t+1})+L_{_{\rm{NML}}}(x_{t+2}^{n}) \right) \right\} -\epsilon . \end{aligned}$$

We accept $H_{1}$ if $h_{2}(x^{n}; t, \epsilon )>0$, otherwise accept $H_{0}$. We call this test the 2*nd MDL test*.

Under the assumption $(1/n)L_{_{\rm{NML}}}(x^{t+1}_{1})\approx (1/n)( L_{_{\rm{NML}}}(x_{1}^{t-1})+ L_{_{\rm{NML}}}(x_{t}x_{t+1}))$ and $(1/n)L_{_{\rm{NML}}}(x^{n}_{t})\approx (1/n)(L_{_{\rm{NML}}}(x_{t}x_{t+1})+L_{_{\rm{NML}}}(x_{t+2}^{n})),$ we have

18 $$\begin{aligned} \Psi ^{(2)}_{t}\approx 2h_{2}(x^{n};t, \epsilon ) +2\epsilon . \end{aligned}$$

This implies that the 2nd D-MDL test is equivalent with testing whether the 2nd D-MDL is larger than $2\epsilon$ or not. Intuitively, if the degree of two-step change exceeds significantly that of one-step change as time increases, then $H_{1}$ is accepted. Thus the basic performance of discrimination of the 2nd D-MDL can be reduced to that of the 2nd D-MDL test.

The following theorem shows the basic property of the 2nd D-MDL test.

##### Theorem 2.3

Type I and II error probabilities for the 2nd D-MDL test are upper bounded as follows:

19 $$\begin{aligned} \mathrm{Type\ I\ error\ prob.}< & {} \exp \left[ -n\left( \epsilon -\frac{\log C_{t}C_{n-t}}{n}\right) \right] , \end{aligned}$$

20 $$\begin{aligned} \mathrm{Type\ II\ error\ prob.}\le & {} \exp \left[ -n\left( d(p_{_{\rm{NML(t)}}},p_{_{\theta _{2}*\theta _{3} *\theta _{4}}})-\frac{\log C_{t-1}C_{2}C_{n-t+1}}{2n}- \frac{\epsilon }{2} \right) \right] , \end{aligned}$$

where $C_{n}$ is the parametric complexity as in Eq. (2), *d* is the Bhattacharyya distance as in Eq. (12) and

$$\begin{aligned} p_{_{\rm{NML}}(t)}(x^{n})= & {} \frac{\max _{\theta }p(x^{t}_1;\theta )}{\sum _{y^{t}_1}\max _{\theta }p(y^{t}_1;\theta )}\cdot \frac{\max _{\theta }p(x^{n}_{t+1};\theta )}{\sum _{y^{n}_{t+1}}\max _{\theta }p(y^{n}_{t+1};\theta )},\\ p_{_{\theta _{2}*\theta _{3}*\theta _{4}}}(x^{n})= & {} p(x^{t-1}_{1};\theta _{2})p(x_{t}x_{t+1};\theta _{3})p(x_{t+2}^{n};\theta _{4}). \end{aligned}$$

This theorem can be proven similarly with Theorem 2.2 Type I probability in Eq. (19) will be used for determining the threshold in “Sequential change sign detection with D-MDL”.

### Sequential change sign detection with D-MDL

In previous sections, we considered how to measure the change sign score at a specific time point *t*. In order to detect change signs sequentially for the case where there exist multiple change points, we can conduct sequential change sign detection using D-MDL in a similar manner with the study^14^. We give two variants of the sequential algorithms. One is the sequential D-MDL algorithm with *fixed windowing* while the other is that with *adaptive windowing*. In the former, we prepare a local window of fixed size to calculate D-MDL at the center of the window. We then slide the window to obtain a sequence of D-MDL change scores as with the study^14^ (see also the study^42^ for local windowing). We raise an alarm when the score exceeds the predetermined threshold $\beta$. The algorithm is summarized as follows:

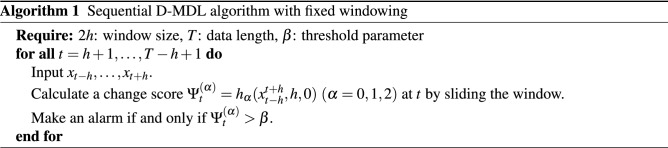



In the study^43^, the sequential algorithm with adaptive windowing (SCAW) was proposed by combining the 0th D-MDL with ADWIN algorithm^9^ (see also the study^44^ for adaptive windowing) where the window grows until the maximum of the MDL change statistics in the window exceeds a threshold. Once it exceeds the threshold, we drop the data earlier than the time point where the maximum is achieved and the window shrinks. Then the process restarts. It outputs the size of window whenever a change point is detected.

According to the study^43^, for the window size *w*, the threshold $\epsilon _{w}$ for $w \Psi ^{(0 )}$ is set so that the total number of false alarms is finite. This is set as follows: For some parameter $\delta >0$, when the parameter is *d*-dimensional,

21 $$\begin{aligned} \epsilon _{w}=(2+d/2+\delta )\log w +\log (1/\delta ). \end{aligned}$$

### Hierarchical sequential D-MDL algorithm

Practically, we combine the algorithm with adaptive windowing for the 0th D-MDL and the algorithms with fixed windowing for the 1st and 2nd D-MDL. We call this algorithm the *hierarchical sequential D-MDL algorithm*. It is designed as follows. We first output not only the 0th D-MDL score but also a window size with the 0th D-MDL with adaptive windowing and raise an alarm when the window shrinks, i.e., Eq. (21) is satisfied for some time in the window. We then output the 1st and 2nd D-MDL scores using the window produced by the 0th D-MDL and raise alarms when for some time in the window, the 1st or 2nd D-MDL exceeds the threshold so as to expect the 1st and 2nd D-MDL to detect change signs before the window shrinkage. Note that the window shrinks only with the 0th D-MDL, but neither with the 1st nor 2nd D-MDL.

In this algorithm, for the window size *w*, the threshold for the 1st D-MDL score $w\Psi ^{(1)}_{t}$ is determined so that Type I error probability in Eq. (15) is less than the confidence parameter $\delta _{1}$. That is, from Eqs. (15) and (3), letting the threshold be $\epsilon _{w}^{(1)}=\epsilon w ,$ we use Eq. (3) ignoring *O*(1) term to obtain

$$\begin{aligned} \mathrm{Type\ I\ prob.}< & {} \exp (-\epsilon _{w}^{(1)}+\log C_{t}C_{n-t})\\\approx & {} \exp (-\epsilon _{w}^{(1)}+(d/2)\log (w/2)\times 2)\le \delta _{1}. \end{aligned}$$

This yields

22 $$\begin{aligned} \epsilon _{w}^{(1)}\ge d\log (w/2)+\log (1/\delta _{1}). \end{aligned}$$

We employ the righthand side of Eq. (22) as the threshold of an alert of the 1st D-MDL.

The threshold $\epsilon ^{(2)}_{w}$ for the 2nd D-MDL score $w\Psi ^{(2)}_{t}$ can also be derived similarly with the 1st one. Note that by Eq. (18), the threshold is 2 times the accuracy parameter for the hypothesis testing. Letting $\delta _{2}$ be the confidence parameter, we have

23 $$\begin{aligned} \epsilon _{w}^{(2)}\ge 2(d\log (w/2)+\log (1/\delta _{2})). \end{aligned}$$

We employ the righthand side of Eq. (23) as the threshold of an alert of the 2nd D-MDL. In practice, $\delta _{1}$ and $\delta _{2}$ are estimated from data (see “Data modeling”).

The hierarchical sequential D-MDL algorithm is summarized as follows:

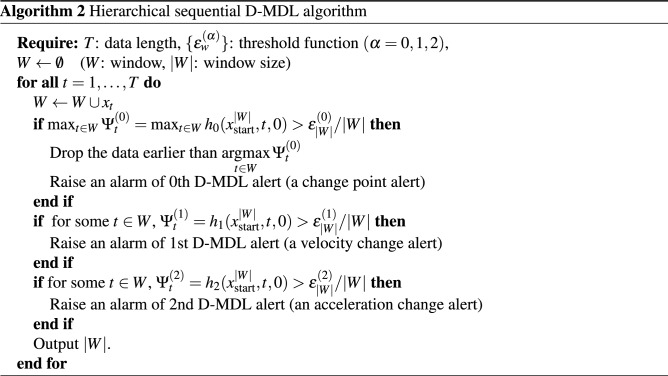



## Result I: experiments with synthetic data

### Datasets

To evaluate how well D-MDL performs for abrupt/gradual change detection, we consider two cases; multiple mean change detection and multiple variance one.

In the case of multiple mean change detection, we constructed synthetic datasets as follows: Each datum was independently drawn from the Gaussian distribution $\mathcal {N}(\mu _{t}, 1)$ where the mean $\mu _{t}$ abruptly/gradually changed over time according to the following rule: In the case of abrupt changes,

$$\begin{aligned} \mu _{t}&= 0.3 \sum _{i=1}^{9} (10-i) H(n-1000i), \end{aligned}$$

where *H*(*x*) is the Heaviside step function that takes 1 if $x> 0$ otherwise 0. In the case of gradual changes, *H* is replaced with the following continuous function:

$$\begin{aligned} S(x) = {\left\{ \begin{array}{ll} 0 &{} (x< 0), \\ x/300 &{} ( 0 \le x < 300), \\ 1 &{} (x \ge 300). \end{array}\right. } \end{aligned}$$

In the case of multiple variance change detection, each datum was independently drawn from the Gaussian distribution $\mathcal {N}(0, \sigma _{t}^{2})$ where the variance $\sigma _{t}^{2}$ abruptly/gradually changed over time according to the following rule: In the case of abrupt changes,

$$\begin{aligned} \log { \sigma _{t} } = 0.1 \sum _{i=1}^{9} (10-i) H(n-1000i). \end{aligned}$$

In the case of gradual changes, *H* is replaced with *S* as with the multiple mean changes.

We define a sign of a gradual change as the starting point of that change. In all the datasets, change points for abrupt changes and change signs for gradual changes were set at nine points: $t=1000$, 2000, $\dots$, 9000.

### Evaluation metric

For any change detection algorithm that outputs change scores for all time points, letting $\beta$ be a threshold parameter, we convert change-point scores $\{ s_{t} \}$ into binary alarms $\{ a_{t} \}$ as follows:

$$\begin{aligned} a_{t} = {\left\{ \begin{array}{ll} 1 &{} (s_{t} > \beta ), \\ 0 &{} (\mathrm {otherwise}). \end{array}\right. } \end{aligned}$$

By varying $\beta$, we evaluate the change detection algorithms in terms of benefit and false alarm rate defined as follows: Let *T* be a maximum tolerant delay of change detection. When the change truly starts from $t^{*}$, we define *benefit* of an alarm at time *t* as

$$\begin{aligned} b(t; t^{*}) = {\left\{ \begin{array}{ll} 1 - \frac{ | t-t^{*} | }{T} &{} ( 0 \le |t - t^{*} | < T), \\ 0 &{} (\mathrm {otherwise}), \end{array}\right. } \end{aligned}$$

where $t^{*}$ is a change point for abrupt change, while it is a sign for gradual change.

The total benefit of alarm sequence $a_{0}^{n-1}$ is calculated as

$$\begin{aligned} B(a_{0}^{n-1}) = \sum _{k=0}^{n-1} a_{k} b(k; t^{*}). \end{aligned}$$

The number of *false alarms* is calculated as

$$\begin{aligned} N(a_{0}^{n-1}) = \sum _{k=0}^{n-1} a_{k} \Theta (b(k; t^{*}) = 0). \end{aligned}$$

where $\Theta (t)$ takes 1 if and only if *t* is true, otherwise 0. We evaluate the performance of any algorithm in terms of AUC (Area under curve) of the graph of the total benefit $B / \sup _{\beta } B$, against the false alarm rate (FAR) $N / \sup _{\beta } N$, with $\beta$ varying.

### Methods for comparison

In order to conduct the sequential D-MDL algorithm, we employed the univariate Gaussian distribution whose probability density function is given by Eq. (7).

We employed three sequential change detection methods for comparison:

1. *Bayesian online change point detection* (BOCPD)^11^: A retrospective Bayesian online change detection method. It originally calculates the posterior of run length. We modified it to compute a change score by taking the expectation of the reciprocal of run length with respect to the posterior.
2. *ChangeFinder* (CF)^4^: A state-of-the-art method of abrupt change detection.
3. *ADWIN2*^9^: A change detection method with adaptive windowing.

We conducted the sequential D-MDL algorithms with fixed window size in order to investigate their most basic performance in terms of the AUC metric. The sequential D-MDL algorithm with adaptive windowing outputs the window size rather than the D-MDL values themselves, hence in order to evaluate the effectiveness of the magnitude of D-MDL, the sequential D-MDL with fixed windowing is a better target for the comparison. All of CF, BOCPD, and ADWIN2 had some parameters, which we determined from five training sequences drawn from the data generation mechanism so that the AUC scores were made the largest.

### Results

The performance comparison is summarized in Table 1. We see that both for the datasets, in the case of abrupt changes, the 0th D-MDL performs best, while in the case of gradual changes, the 1st D-MDL performs best and the 2nd D-MDL performs worse than the 1st but better than the 0th. That matches our intuition. Because the 0th D-MDL was designed so that it could detect abrupt changes while the 1st one was designed so that it could detect starting points of gradual changes.

**Table 1:** Average AUC scores ± standard deviation on the synthetic datasets.

	Multiple-mean-changing datasets		Multiple-variance-changing datasets	
	Abrupt	Gradual	Abrupt	Gradual
BOCPD	$0.546 \pm 0.059$	$0.416 \pm 0.038$	$0.574 \pm 0.022$	$0.354 \pm 0.029$
CF	$0.591 \pm 0.031$	$0.505 \pm 0.029$	$0.608 \pm 0.023$	$0.506 \pm 0.018$
ADWIN2	$0.500 \pm 0.000$	$0.542 \pm 0.016$	$0.500 \pm 0.000$	$0.458 \pm 0.024$
D-MDL (0th)	$\mathbf{0.918 \pm 0.016}$	$0.614 \pm 0.041$	$\mathbf{0.825 \pm 0.031}$	$0.521 \pm 0.050$
D-MDL (1st)	$0.480 \pm 0.006$	$\mathbf{0.623 \pm 0.020}$	$0.272 \pm 0.016$	$\mathbf{0.533 \pm 0.023}$
D-MDL (2nd)	$0.494 \pm 0.006$	$0.620 \pm 0.003$	$0.486 \pm 0.004$	$0.526 \pm 0.003$

## Result II: applications to COVID-19 pandemic analysis

Since the beginning of 2020, many regions/countries have suffered from the epidemic of COVID-19. The purpose of our analysis is to demonstrate the importance of monitoring the dynamics of the epidemic through detecting the occurrence of drastic outbreaks and their signs. We define *outbreak* as a significant increase in the number of cases in a region/country. We note that to contain the spread of COVID-19, many countries have enacted social distancing policies, e.g., stay-at-home order, closing non-essential services, and limiting travel. We thus also relate the results of our analysis to social distancing events.

In particular, we are mainly concerned with the following two problems:

1. How early are the outbreak signs detected prior to outbreaks?
2. How are the outbreaks/outbreak signs related to the social distancing events?

As a byproduct, the analysis of the dynamics of the basic reproduction number *R*0^37^ is conducted, which can serve as supplementary information to the particular value estimated from the SIR model^45^.

### Data source

We studied the data provided by European Centre for Disease Prevention and Control (ECDC) which can be accessed through the link https://www.ecdc.europa.eu/en/publications-data/download-todays-data-geographic-distribution-covid-19-cases-worldwide. In this paper, we focused on the first wave because various factors made the situations very complicated in later waves, e.g., virus mutations^31–33^, people being tired of social distancing and the mixture of two waves in the transition period. In particular, we studied 37 countries with no less than 10,000 cumulative cases by Apr. 30, 2020 since some countries started to ease the social distancing around the date. More details about these countries can be found in Sec. 2 of the supplementary information. It is worth mentioning that the proposed method can be applied to any region/country where there is a COVID-19 epidemic because the input to the method is only the number of cases. In practice, we suggest starting to run our algorithm when the spread of the virus into the region of concern through local infections begins but not when the cases are just imported.

### Data modeling

We studied two data models by considering the value of *R*0, which by definition is the product of transmissibility, the average contact rate between susceptible and infected individuals, and the duration of infectiousness^45^. At the initial phase of an epidemic, *R*0 is larger than one^37^. And the cumulative cases may grow exponentially^46–49^. We thus employed the Malthusian growth model^50^ because it is widely used for characterizing the early phase of an epidemic^48,49^. In particular, the cumulative cases at time *t*, *C*(*t*), grows according to the following equation:

24 $$\begin{aligned} C(t) = C(0)\exp (rt), \end{aligned}$$

where *C*(0) is the number of cases at the start of an epidemic, and *r* is the growth rate of daily new cases. In the experiments, we took the logarithm of *C*(*t*) to obtain the linear regression of the logarithm growth with respect to time as follows:

25 $$\begin{aligned} \log C(t) = rt + \log C(0). \end{aligned}$$

We modeled the residual error of the linear regression using the univariate Gaussian. See Sec. 3 in the supplementary file for the detail of calculation of the MDL change statistics for this model. When a change is detected in the modeling of the residual error, we examine the increase/decrease in the coefficient of the linear regression, i.e., *r*. We expect to detect changes in the parameter of the *exponential modeling* to monitor the increase/decrease of *R*0 because $R0-1$ is proportional to *r*^47^.

In later phases, the exponential growth pattern may not hold. For instance, when $R0 < 1$, daily new cases would continue to decline and cease to exist^37^. Considering the complicated real scenarios, epidemic models with certain assumptions on the growth rate or *R*0 may not fit an epidemic at a given time. Therefore, we employed the univariate Gaussian distribution as in Eq. (7) to directly model the number of daily new cases, without assuming any patterns of the growth. The change in the parameter of the *Gaussian modeling* may reveal the relation between one and *R*0, i.e., $R0 > 1$ when daily new cases increase significantly or $R0 < 1$ when daily new cases decrease significantly.

We conducted the hierarchical sequential D-MDL algorithm as in “Hierarchical sequential D-MDL algorithm”. The confidence parameter $\delta$ for the 0th D-MDL as in Eq. (21) was set to be 0.05. Those for the 1st and 2nd D-MDL, i.e. $\delta _{1},\delta _{2}$ as in Eqs. (22), (23) were determined as follows: We calculated the D-MDL scores around the time when the initial warning was announced by an authority; we determined $\delta _{1},\delta _{2}$ so that the score was the threshold. For example, the initial warning for Japan was set on Feb. 27, when the government required closing elementary, junior high and high schools. If the resulting $\delta _{1},\delta _{2}$ was larger than 1, it was set to be 0.99 because of the concept of confidence parameter. More details about the implementation are provided in Sec. 4 of the supplementary information.

### Case study

**Figure 1:**
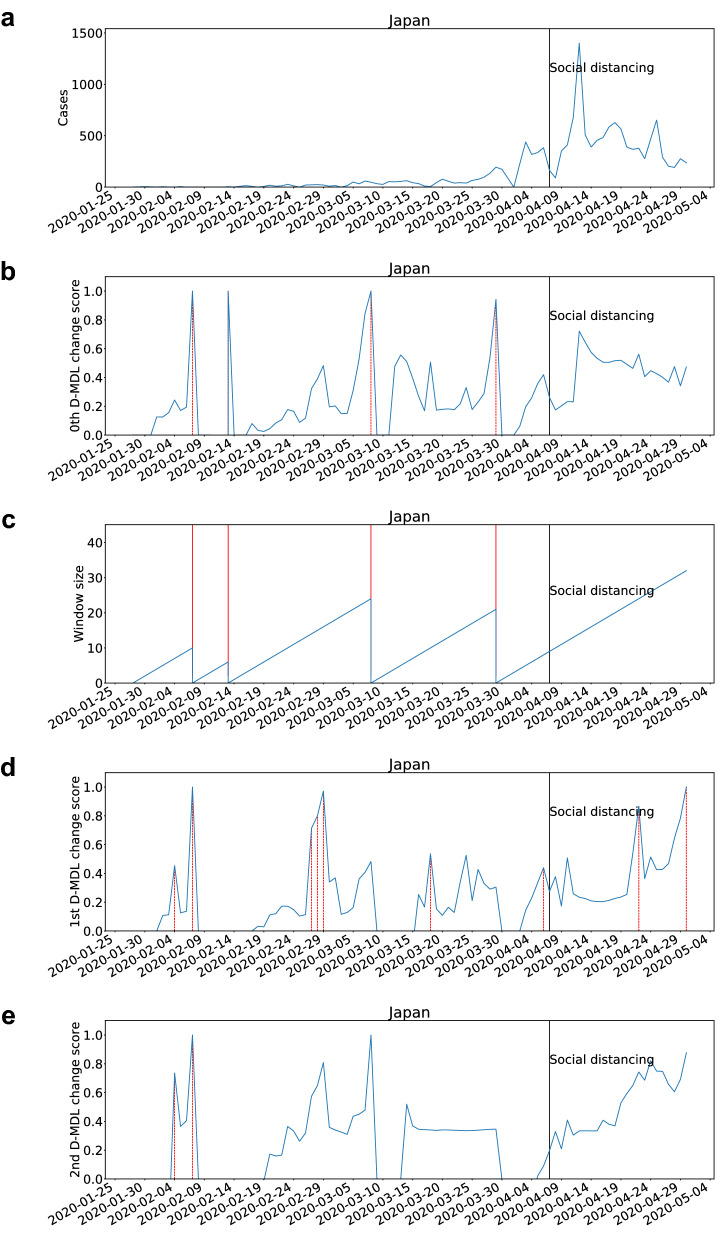
The results for Japan with the Gaussian modeling. The date on which the social distancing was implemented is marked by a solid line in black. (**a**) The number of daily new cases. (**b**) The change scores produced by the 0th D-MDL where the line in blue denotes values of scores and dashed lines in red mark alarms. (**c**) The window sized for the sequential D-MDL algorithm with adaptive windowing where lines in red mark the shrinkage of windows. (**d**) The change scores produced by the 1st D-MDL. (**e**) The change scores by the 2nd D-MDL. In all figures the negative scores are omitted.

**Figure 2:**
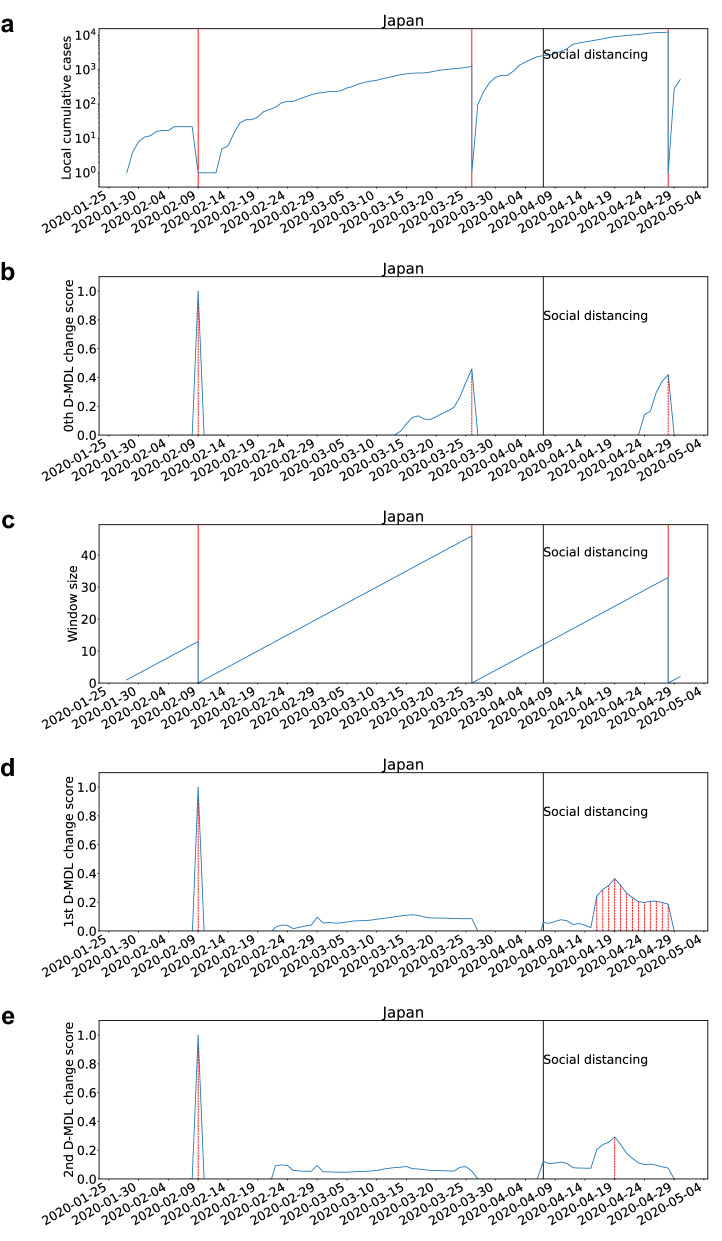
The results for Japan with the exponential modeling. The label “Local cumulative cases” in subfigure **(a)** means that the cumulative cases only accumulate daily cases from each starting date of change detection and would be set to zero after each change detected by the 0th D-MDL. The date on which the social distancing was implemented is marked by a solid line in black. (**a**) The number of cumulative cases. (**b**) The change scores produced by the 0th D-MDL where the line in blue denotes values of scores and dashed lines in red mark alarms. (**c**) The window sized for the sequential D-MDL algorithm with adaptive windowing where lines in red mark the shrinkage of windows. (**d**) The change scores produced by the 1st D-MDL. (**e**) The change scores produced by the 2nd D-MDL.

We present a representative case study of Japan due to space consideration. For results of all the studied countries, please refer to Sec. 5 of the supplementary information. In Japan, state of emergency as the social distancing event was issued on Apr. 7. The results are presented in Fig. 1 and Fig. 2 for the Gaussian modeling and the exponential modeling, respectively. Change scores were normalized into the range [0, 1]. The data of Japan did not include the confirmed cases from ‘Diamond Princess’.

With the Gaussian modeling, there were several alarms raised before the social distancing event. For each alarm raised by the 0th D-MDL, the interpretation can be a statistically significant increase in cases, with reference to Fig. 1a. Hereafter, a change that was detected by the 0th D-MDL and that corresponded to the increase of cases was regarded as an outbreak, which instantiates our definition of outbreak. The outbreak detection is the classic change detection. We further relate it to *R*0. Around the dates of the alarms, $R0 > 1$ was considered since we can confirm that the new infections resulted from community transmission. Correspondingly, *R*0 was estimated around 2.5 in early March by an epidemiological study^51^. When the 0th D-MDL raised an alarm, the window size shrank to zero. Before that, both the 1st and the 2nd D-MDL raised alarms, which are interpreted as the changes in the velocity and the acceleration of the increase of cases, respectively. We can conclude that the 1st and the 2nd D-MDL were able to detect the signs of the outbreak by examining the velocity and the acceleration of the spread. The sign detection is the new concept with which we propose to supplement the classic change detection. The 0th D-MDL raised no alarms about outbreaks after the event. We think the social distancing played a critical role in containing the spread because it can significantly suppress *R*0 through reducing the contact rate. The 1st D-MDL still raised alarms, which were signs of decreases in the cases.

As for the exponential modeling, there were alarms raised by the 0th D-MDL both before and after the social distancing event. By looking at the growth pattern of local cumulative cases in Fig. 2a, we can see that all the alarms were about the cessations of the exponential growth. Moreover, we checked that the alarms were associated with decreases in the coefficient of the linear regression. Therefore, we concluded that all the alarms indicated significant decreases in *R*0. Although the last two alarms were raised on Mar. 26 and Apr. 28, the dates as the change points were within the windows as of Mar. 26 and Apr. 28, and were identified as Mar. 12 and Apr. 18, respectively. There was an epidemiological study^51^ which showed the effectiveness of the initial warning announced on Feb. 27 at reducing *R*0. As a result, it demonstrated that our method can effectively identify the decrease in *R*0 around Mar. 12. According to the result, our method identified another decrease in *R*0 around Apr. 18, which we think was mainly due to the social distancing event on Apr. 7. Therefore, our method based on the exponential modeling also confirmed that social distancing was very effective at containing the spread. The alarms raised by the 1st and 2nd D-MDL demonstrated the capability of the sign detection.

As a comparison, the Gaussian modeling was effective at estimating the relation between one and *R*0 while the exponential modeling was able to monitor the change in the value of *R*0. The two models form a complementary relation on monitoring the dynamics of *R*0. For instance, for Japan, the Gaussian modeling showed that the value of *R*0 reminded at a value larger than one, and the exponential modeling showed that its value decreased during the studied period. Due to the difference in the modeling, the changes detected by the 0th D-MDL were at different dates between the Gaussian modeling and the exponential modeling. In terms of the sign detection, both the Gaussian modeling and the exponential modeling were effective.

### Summarization on individual countries

This section summarizes several statistics about the change detection results in Table 2 and presents two interesting observations. The first is about how early the signs can be detected prior to changes. For the countries studied, there were 106 and 54 changes in total detected by the Gaussian modeling and the exponential modeling, respectively. There were more changes detected by the Gaussian modeling because daily cases would significantly change with either $R0>1$ or $R0<1$ while it may take relatively longer time for significant changes in *R*0. The number of changes whose signs were detected by either the 1st or the 2nd D-MDL was 68 and 26 for the Gaussian modeling and the exponential modeling, respectively, representing high detection rates. For each change whose signs were detected, we measured the time difference between the earliest sign alarm and the change alarm. For the Gaussian modeling which can detect outbreaks, the time difference in terms of the number of days is 6.25 (mean) ± 6.04 (standard deviation). Considering the fast spread, six days can buy us considerably long time to prepare for an outbreak, and even to avoid a potential outbreak.

In particular, with the Gaussian modeling, the 1st D-MDL detected signs for 65 changes and the 2nd D-MDL detected signs for 27 changes. The smaller number by the 2nd D-MDL might be because the 1st D-MDL is better at detecting starting points of gradual changes, and is consistent with results on the synthetic datasets as in Table 1. The number of days before which the 1st D-MDL detected signs was 6.35 ± 5.91, and the number for the 2nd D-MDL was 5.56 ± 6.50. Note that not all the changes allowed for sign detection since the 1st D-MDL and the 2nd D-MDL sign detection require one more and two more data points in the window than the 0th D-MDL, respectively. The number of changes allowing for a 1st D-DML sign was 88 while the number for a 2nd D-DML sign was 81. Hence, it turned out that some changes occurred too quickly before signs can be detected. The analysis of the results obtained by the exponential modeling is similar and omitted for space consideration.

**Table 2:** Summarization of statistics where changes represent the alarms raised by the 0th D-MDL and signs were alarms raised by either the 1st or the 2nd D-MDL.

Measurement	Gaussian	Exponential
Total number of changes	106	54
Number/percentage of changes whose signs were detected by either the 1st or the 2nd D-MDL	68/$64\%$	26/$48\%$
Number of days before which the first sign was detected by either the 1st or the 2nd D-MDL for a change	$6.25\pm 6.04$	$11.27\pm 7.72$
Total number of changes that allowed for the 1st/2nd D-MDL sign detection	88/81	53/53
Number of changes whose signs were detected by the 1st/2nd D-MDL	65/27	26/6
Number of days before which the first 1st D-MDL sign was detected for a change	$6.35\pm 5.91$	$11.27\pm 7.72$
Number of days before which the first 2nd D-MDL sign was detected for a change	$5.56\pm 6.50$	$5.17\pm 5.67$
Number of changes and signs before the event for the downward countries	$4.30\pm 2.79$	-
Number of changes and signs before the event for the non-downward countries	$5.96\pm 4.22$	-
Number of days from event’s date to the first downward change’s date for downward countries	$30.00\pm 8.28$	-
Number of days from event’s date to Apr. 30 for non-downward countries	$36.54\pm 7.28$	-
Number of decreasing changes and signs for the downward countries	-	$10.60\pm 6.67$
Number of decreasing changes and signs the non-downward countries	-	$9.96\pm 9.65$

Second, we observed that on average, countries responding faster in terms of a smaller number of alarms raised by the Gaussian modeling before the social distancing event saw a quicker contraction of daily cases. As of Apr. 30, the curve of daily cases in many countries had been flatten, and even started to be downward. Therefore, alarms for declines in the number of daily cases from the global peak number were raised for ten countries including Austria, China, Germany, Iran, Italy, Netherlands, South Korea, Spain, Switzerland, and Turkey. These countries are referred to as *downward countries*. In total, the number of all kinds of alarms raised before the event for downward countries was 4.30 ± 2.79 while it was 5.96 ± 4.22 for other countries. Therefore, if the social distancing is a viable option, it is suggested that the action should better be taken before it is late, e.g., later than four alarms. We further measured that it took an average of 30 days to suppress the spread if prompt social distancing policies were enacted. By contrast, the average number of days from the date of social distancing event to Apr. 30 was nearly 37 for non-downward countries, which was considerably more than the time used for suppressing the spread in downward countries. The results of the exponential modeling confirmed the above observation. In particular, changes and their signs which corresponded to decreases in *R*0 for the downward countries were more than those for the non-downward countries.

### Limitations and challenges of the COVID-19 analysis

Since the proposed method only examines the number of COVID-19 cases, the analysis can only give an overall estimation of the dynamics of the pandemic which are the results of the joint effects of various kinds of physical factors including the characteristics of the virus, human mobility patterns, mask usage, vaccine coverage, environmental factors, and etc. When changes happen to any one of the physical factors, e.g., virus mutations or the entry of the virus into sewage^52^, the number of cases may change. Accordingly, the major limitation of the proposed method is that itself cannot associate the detected changes, either outbreaks or their signs, with a particular physical factor.

We were concerned with detecting signs of the first wave of COVID-19. Although we employed the Gaussian model and the exponential growth model in computing D-MDL, such models might not be necessarily most appropriate for dealing with later waves, since a number of waves are mixed in the transition periods. One of challenges is to consider more sophisticated models such as latent variable models in dealing with later waves.

## Conclusion

This paper has proposed a novel methodology for detecting signs of changes from a data stream. The key idea is to use the differential MDL change statistics (D-MDL) as a sign score. This score can be thought of as a natural extension of the differentials of the Kullback–Leibler divergence for measuring the degree of changes to the case where the true mechanism for generating data is unknown. We have theoretically justified D-MDL using the hypothesis testing framework and have empirically justified the sequential D-MDL algorithm using the synthetic data. On the basis of the theory of D-MDL, we have applied it to the COVID-19 pandemic analysis. We have observed that the 0th D-MDL found change points related to outbreaks and that the 1st and 2nd D-MDL were able to detect their signs several days earlier than them. We have further related the change points to the dynamics of the basic reproduction number *R*0. We have also found that the countries with no more than five changes/change signs before the implementation of social distancing tended to experience the decrease in the number of cases considerably earlier. This analysis is a new promising approach to the pandemic analysis from the view of data science.

Change detection, which aims to detect points in a sequence of random variables at which the probability distribution change, has been studied for decades and has wide applications, such as event detection, failure detection, malware detection, etc^4,14,43^. Change sign detection proposed in this paper aims to detect early warning signals of such changes by identifying the speed and acceleration of changes in the probability distribution, and therefore has the same applicability as the change detection.

Future work includes studying how we can integrate the change analysis such as our methodology with the conventional simulation studies such as SIR model. It is expected that our data science approach has a complementary relation with the simulation approach and gives new insights into epidemiology. Moreover, we plan to study later waves which are more complicated situations than the first wave.

## Supplementary Information

Supplementary Information.
