# Supplementary material for: Change sign detection with differential MDL change statistics and its applications to COVID-19 pandemic analysis
Source: Sci Rep. 2021 Oct 5;11:19795. doi: 10.1038/s41598-021-98781-4 (PMC8492813; doi:10.1038/s41598-021-98781-4)
Supplement: Supplementary file 1 — Supplementary Information. [file 41598_2021_98781_MOESM1_ESM.pdf]

Supplementary Information

Change Sign Detection with Differential MDL Change Statistics and Its Applications to COVID-19 Pandemic Analysis

Kenji Yamanishi[1,*], Linchuan Xu[2,*], Ryo Yuki[1], Shintaro Fukushima[1], and Chuan-hao Lin[1]

[1]Graduate School of Information Science and Technology, The University of Tokyo, Tokyo, 113-8656, Japan
[2]Department of Computing, The Hong Kong Polytechnic University, Hung Hom, Kowloon, Hong Kong
[*]yamanishi@mist.i.u-tokyo.ac.jp, linch.xu@polyu.edu.hk

The supplementary materials contain the following information:

- Section 1 presents the proof of Theorem 2.2.
- Section 2 presents the information of studied data.
- Section 3 gives the details of calculation of the MDL change statistics for the linear regression model.
- Section 4 presents some details about the implementation of our Hierarchical Sequential D-MDL Algorithm presented in Algorithm 2.
- Section 5 introduces additional experiment results.

1 Proof of Theorem 2.2

Let the maximum likelihood estimator of θ_i be $\hat{\theta}_i$ $(i = 0, 1, 2, 3)$. Let us define the event as

$$\begin{aligned} h_1(x_1^n; t, \epsilon) \quad = \quad & -\log p(x_1^t; \hat{\theta}_0(x_1^t)) - \log p(x_{t+1}^n; \hat{\theta}_1(x_{t+1}^n)) + \log C_t + \log C_{n-t} \\ & + \log p(x_1^{t+1}; \hat{\theta}_2(x_1^{t+1})) + \log p(x_{t+2}^n; \hat{\theta}_3(x_{t+2}^n)) - \log C_{t+1} - \log C_{n-t-1} - n\epsilon > 0. \end{aligned}$$

Equivalently,

$$\begin{aligned} & p(x_1^t; \hat{\theta}_0(x_1^t)) p(x_{t+1}^n; \hat{\theta}_1(x_{t+1}^n)) \\ & < \frac{p(x_1^{t+1}; \hat{\theta}_2(x_1^{t+1}))}{C_{t+1}} \cdot \frac{p(x_{t+2}^n; \hat{\theta}_3(x_{t+2}^n))}{C_{n-t-1}} \cdot \exp\left(-n\left(\epsilon - \frac{\log C_t C_{n-t}}{n}\right)\right). \end{aligned} \quad (1)$$

Type I error probability is evaluated as follows: Let the true parameter be θ_0^* and θ_1^*.

$$\sum_{x_1^n \cdots (1)} p(x_1^t; \theta_0^*) p(x_{t+1}^n; \theta_1^*)$$
$$< \sum_{x^n \cdots (1)} \frac{p(x_1^{t+1}; \hat{\theta}_2(x_1^{t+1}))}{C_{t+1}} \cdot \frac{p(x_{t+2}^n; \hat{\theta}_3(x_{t+2}^n))}{C_{n-t-1}} \cdot \exp\left(-n\left(\epsilon - \frac{\log C_t C_{n-t}}{n}\right)\right)$$
$$\leq \left(\sum_{x_1^{t+1}} \frac{p(x_1^{t+1}; \hat{\theta}_2(x_1^{t+1}))}{C_{t+1}}\right)\left(\sum_{x_{t+2}^n} \frac{p(x_{t+2}^n; \hat{\theta}_3(x_{t+2}^n))}{C_{n-t-1}}\right) \cdot \exp\left(-n\left(\epsilon - \frac{\log C_t C_{n-t}}{n}\right)\right)$$
$$= \exp\left(-n\left(\epsilon - \frac{\log C_t C_{n-t}}{n}\right)\right),$$

where we have used the following relations:

$$\sum_{x_1^{t+1}} \frac{p(x_1^{t+1}; \hat{\theta}_2(x_1^{t+1}))}{C_{t+1}} = 1, \quad \sum_{x_{t+2}^n} \frac{p(x_{t+2}^n; \hat{\theta}_3(x_{t+2}^n))}{C_{n-t-1}} = 1.$$

Next we evaluate Type II error probability. Let us define the event as

$$h_1(x_1^n, t, \epsilon) \leq 0 \qquad (2)$$

and let $p_{\mathrm{NML}(t)}(x_1^n) \stackrel{\mathrm{def}}{=} (p(x_1^t; \hat{\theta}_0(x_1^t)/C_t)(p(x_{t+2}^n; \hat{\theta}_1(x_{t+1}^n))/C_{n-t})$. Let the true parameter be θ_2^* and θ_3^*. Then under the event (2),

$$-\log p_{\mathrm{NML}(t)}(x_1^n) \leq -\log p(x_1^{t+1}; \hat{\theta}_2(x_1^{t+1})) - \log p(x_{t+2}^n; \hat{\theta}_3(x_{t+2}^n)) + \log C_{t+1} + \log C_{n-t-1} + n\epsilon$$
$$\leq -\log p(x_1^{t+1}; \theta_2^*) - \log p(x_{t+2}^n; \theta_3^*) + \log C_{t+1} C_{n-t-1} + n\epsilon$$

This implies

$$1 \leq \left(\frac{p_{\mathrm{NML}}(x_1^n)}{p(x_1^{t+1}; \theta_2^*) p(x_{t+2}^n; \theta_3^*)}\right)^{\frac{1}{2}} \exp\left(\frac{1}{2}(\log C_{t+1} C_{n-t-1} + n\epsilon)\right)$$

The Type II error probability is upper-bounded as follows:

$$\sum_{x_1^n \cdots (2)} p(x_1^{t+1}; \theta_2^*) p(x_{t+2}^n; \theta_3^*)$$
$$\leq \sum_{x_1^n \cdots (2)} (p(x_1^{t+1}; \theta_2^*) p(x_{t+2}^n; \theta_3^*)) \left(\frac{p_{\mathrm{NML}}(x_1^n)}{p(x_1^{t+1}; \theta_2^*) p(x_{t+2}^n; \theta_3^*)}\right)^{\frac{1}{2}} \exp\left(\frac{1}{2}(\log C_{t+1} C_{n-t-1} + n\epsilon)\right)$$
$$\leq \sum_{x_1^n} \left(p_{\mathrm{NML}(t)}(x_1^n)(p(x_1^{t+1}; \theta_2^*) p(x_{t+2}^n; \theta_3^*)\right)^{\frac{1}{2}} \exp\left(\frac{1}{2}(\log C_{t+1} C_{n-t-1} + n\epsilon)\right)$$
$$= \exp\left(-n\left(d(p_{\mathrm{NML}(t)}, p_{\theta_2^*} * p_{\theta_3^*}) - \frac{\log C_{t+1} C_{n-t-1}}{2n} - \frac{\epsilon}{2}\right)\right),$$

where d and $p_{\theta_2^*} * p_{\theta_3^*}$ are defined as in Theorem 2.1 and 2.2. This completes the proof. □

2 Data Information

We employed the data provided by European Centre for Disease Prevention and Control (ECDC) via https://www.ecdc.europa.eu/en/publications-data/download-todays-data-geographic-distribution-covid-19-cases-worldwide. For information, there are 37 countries that had no less than 10,000 cases in total by Apr. 30, including Austria, Belarus, Belgium, Brazil, Canada, Chile, China, Ecuador, France, Germany, India, Indonesia, Iran, Ireland, Israel, Italy, Japan, Mexico, Netherlands, Pakistan, Peru, Poland, Portugal, Qatar, Romania, Russia, Saudi Arabia, Singapore, South Korea, Spain, Sweden, Switzerland, Turkey, Ukraine, United Arab Emirates, United Kingdom, United States of America in alphabetic order.

We collected the date on which the social distancing was implemented from the information listed in the IHME COVID-19 predictions via `https://covid19.healthdata.org/united-kingdom`. If a certain country is not listed in the website, we referred to the Wikipedia page for the COVID-19 pandemic of the country, e.g., the COVID-19 pandemic in South Korea `https://en.wikipedia.org/wiki/COVID-19_pandemic_in_South_Korea`. For information, the dates are Austria: 2020-3-16, Belarus: 2020-4-9, Belgium: 2020-3-18, Brazil: 2020-3-24, Canada: 2020-3-17, Chile: 2020-3-26, China: 2020-1-23, France: 2020-3-17, Germany: 2020-3-16, India: 2020-3-25, Indonesia: 2020-4-6, Iran: 2020-3-24, Ireland: 2020-3-12, Israel: 2020-3-15, Italy: 2020-3-9, Japan: 2020-4-7, Mexico: 2020-3-23, Netherlands: 2020-3-15, Pakistan: 2020-3-24, Peru: 2020-3-16, Poland: 2020-3-24, Portugal: 2020-3-19, Qatar: 2020-3-23, Romania: 2020-3-23, Russia: 2020-3-30, Saudi Arabia: 2020-3-24, Singapore: 2020-4-7, South Korea: 2020-2-25, Spain: 2020-3-13, Sweden: 2020-3-24, Switzerland: 2020-3-16, Turkey: 2020-3-21, Ukraine: 2020-3-25, United Arab Emirates: 2020-3-31, United Kingdom: 2020-3-24, United States of America: 2020-3-19. Ecuador was excluded from the list above because the social distancing is introduced to be related to changes incurred by declines in the number of cases and there was a very large number of cases in the initial phase of the epidemic in Ecuador. The large number might be an outlier due to the data collection procedure, and would make any changes after that date downward changes. But we still studied the change/change sign detection for Ecuador.

3 MDL change statistics for linear regression

We consider the linear regression model defined as follows:

$$X^n = (x_1, \dots x_n)^\top = W_n^\top \beta + \epsilon, \;\; \epsilon \sim \mathcal{N}(0, \sigma^2 I_n),$$

$$W_n = \begin{pmatrix} 1 & 1 & 1 & \dots & 1 \\ 1 & 2 & 3 & \dots & n \end{pmatrix}^\top \in \mathbb{R}^{n \times 2},\; \beta \in \mathbb{R}^2,$$

Let us define the class of linear regression by

$$\mathcal{P} = \Big\{ p(X^n; \theta) = \frac{1}{(\sqrt{2\pi}\sigma)^d} \exp\left(-\frac{||X - W_n^\top \beta||^2}{2\sigma^2} \right) :$$
$$\theta = (\beta, \sigma^2) \in \mathbb{R}^3,\; n = 1, 2, \dots \Big\}.$$

Then according to [1], the NML codelength for x^n relative to this class is

$$L_{\text{NML}}(x^n) = \frac{n}{2}\log\hat{\sigma}^2 + \log\frac{R}{\sigma_{min}^2} - \log\Gamma\left(\frac{n}{2}-1\right) + \frac{n}{2}\log(n\pi),$$

where $\sigma_{\min}$ and R are hyper-parameters determined so that $\hat{\sigma}_i^2 \geq \sigma_{\min}^2$ and $\|\hat{\beta}\| \leq nR$. $\hat{\sigma}_t^2$ is a maximum likelihood estimator of σ^2 from x_1^t and so on. Thus the 0th MDL change statistics at time t is

$$\begin{aligned}\Psi_t^{(0)} &= \log\frac{\sigma_n^n}{\sigma_t^t\sigma_{n-t}^{n-t}} - \log\frac{R}{\sigma_{min}^2} \\ &\quad -\log\frac{\Gamma(n/2-1)}{\Gamma(t/2-1)\Gamma((n-t)/2-1)} \\ &\quad +\frac{1}{2}(n\log n - t\log t - (n-t)\log(n-t)).\end{aligned}$$

We used this formula for the exponential growth model in Section 4.2 letting $x_t = \log C(t)$ for the number of cumulative cases $C(t)$.

4 Implementation Details

There are three hyper-parameters in our Hierarchical Sequential D-MDL Algorithm, which are δ_0, δ_1 and δ_2 for specifying the threshold of changes in the 0th D-MDL, the 1st D-MDL, and the 2nd D-MDL, respectively. δ_0 was set to be 0.05. δ_1 and δ_2 were determined as follows: we calculated the D-MDL scores around the time when the initial warning was announced by an authority; we determined δ_1, δ_2 so that the score was the threshold. If the resulting δ_1, δ_2 were larger than 1, they were set to be 0.99 because of the concept of confidence parameter. In particular, we calculated δ_1 and δ_2 by using the initial warning in Japan, and applied them to all the other countries. The initial warning for Japan was set on Feb. 27, when the government called for voluntary event cancellation.

We determined the starting date of change detection for each country as the date whose past ten days saw an average of at least one case. This is because few cases in a country may be imported from other countries, and may have nothing to do with local community transmission. We implemented our algorithm in Python 3. Detailed instructions about how to conduct the experiments presented in this paper with our implementation are available at `https://github.com/IbarakikenYukishi/differential-mdl-change-statistics`. Moreover, we developed an online detection system based on our methodology which can perform change/sign detection everyday and raise corresponding alarms. The system can be accessed through `https://ibarakikenyukishi.github.io/d-mdl-html/index.html`.

5 Additional Experiment Results

5.1 Case Study of South Korea

The date of the implementation of social distancing was considered as Feb. 25 from which many non-essential services were closed. We present results in Fig. 1 and Fig. 2 for the Gaussian modeling and the exponential modeling, respectively.

With the Gaussian modeling, there were several alarms raised before the social distancing event. Around the dates of the alarms, $R0 > 1$ was considered since we can confirm that the new infections resulted from community transmission. Correspondingly, $R0$ was estimated at 1.5 by an epidemiological study [2]. When the 0th D-MDL raised an alarm, the window size shrank to zero. Before that, both the 1st and the 2nd D-MDL raised alarms, which are interpreted as the changes in the velocity and the acceleration of the increase of cases, respectively. We can conclude that the 1st and the 2nd D-MDL were able to detect the signs of the outbreak by examining the velocity and the acceleration of the spread.

The 0th D-MDL raised several alarms after the event, and the latest ones corresponded to decreases of cases. It is not difficult to tell that the corresponding $R0$ was less than one. We think that the social distancing played a critical role in containing the spread because it can suppress $R0$ through reducing the contact rate, which was supported by studies [2, 3, 4, 5]. Both the 1st and the 2nd D-MDL again demonstrated the capability of early sign detection.

As for the exponential modeling, there were alarms raised by the 0th D-MDL both before and after the social distancing event. By looking at the growth pattern of local cumulative cases in Fig. 2(a), we can see that all the alarms were about the cessations of the exponential growth. Moreover, we checked that the alarms were associated with decreases in the coefficient of the linear regression. Therefore, we concluded that all the alarms indicated the significantly decreases in $R0$. The alarms raised by the 1st and 2nd D-MDL demonstrate the capability of the sign detection.

a

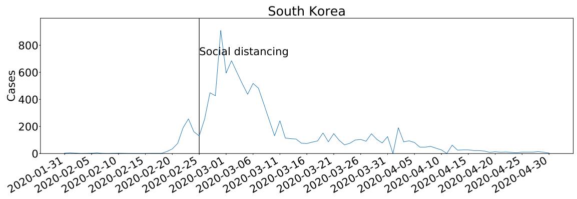

b

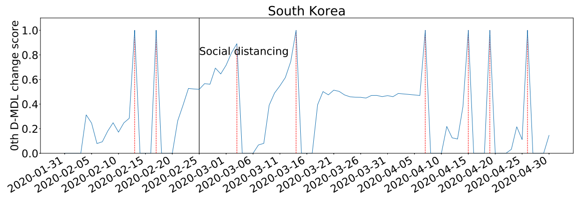

c

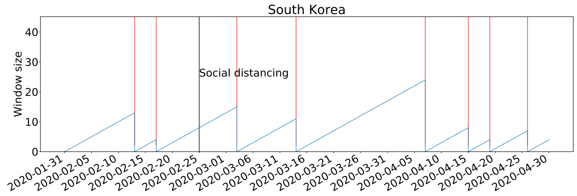

d

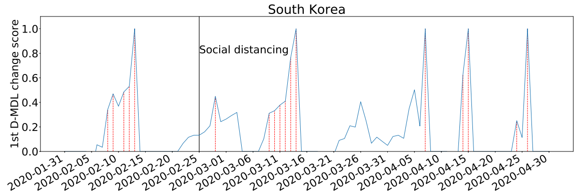

e

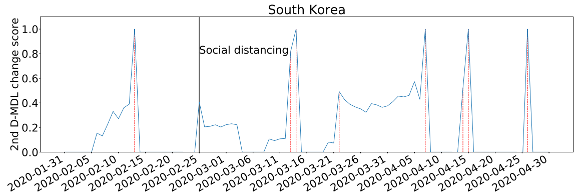

Fig. 1: The results for South Korea with Gaussian modeling. The date on which the social distancing was implemented is marked by a solid line in black. **a,** the number of daily new cases. **b,** the change scores produced by the 0th D-MDL where the line in blue denotes values of scores and dashed lines in red mark alarms. **c,** the window sized for the sequential D-MDL algorithm with adaptive windowing where lines in red mark the shrinkage of windows. **d,** the change scores produced by the 1st D-MDL. **e,** the change scores produced by the 2nd D-MDL.

a

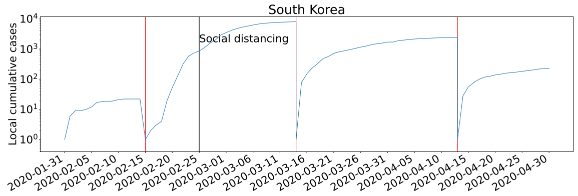

b

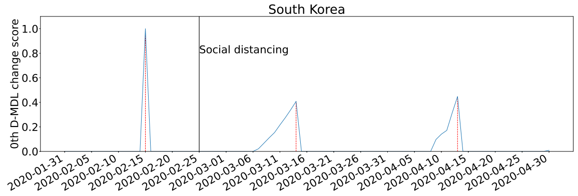

c

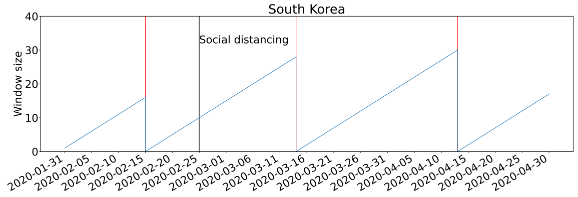

d

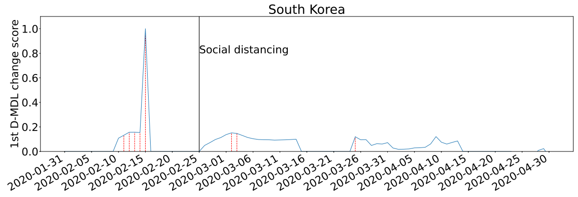

e

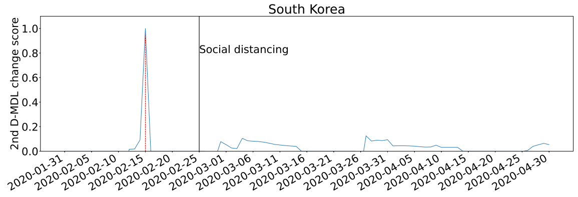

Fig. 2: The results for South Korea with exponential modeling. The date on which the social distancing was implemented is marked by a solid line in black. **a,** the number of cumulative cases. **b,** the change scores produced by the 0th D-MDL where the line in blue denotes values of scores and dashed lines in red mark alarms. **c,** the window sized for the sequential D-MDL algorithm with adaptive windowing where lines in red mark the shrinkage of windows. **d,** the change scores produced by the 1st D-MDL. **e,** the change scores produced by the 2nd D-MDL.

5.2 Results for All the Studied Countries

This section presents the results for all the studied countries with both the Gaussian modeling and the exponential modeling. Since the interpretation for each country is similar and there are many countries,

we omit the analyses. Please refer to the cases studies of Japan and South Korea for more information. It is worth mentioning that there were only two alarms raised by the 0th D-MDL about increases of $R0$, which were in Germany and Singapore, respectively. Note that $R0$ is an inherent property of COVID-19 [6], and its value would not increase unless there are virus mutations which lead to increases in the transmissibility of COVID-19. Accordingly, $R0$ would decrease at most time as the susceptible individuals decrease and social distancing events increase. After checking the figure of daily cases, we conclude that the increase of $R0$ in Germany may be because of a significant increase in testing, and the increase in Singapore may be because daily cases before the alarm were mostly imported from other countries.

a

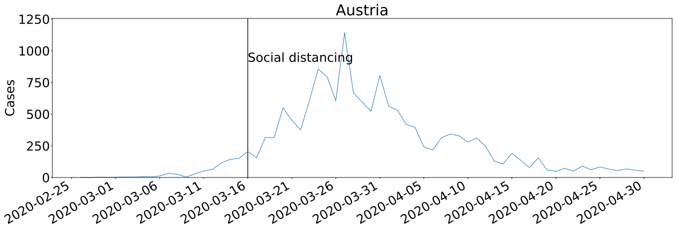

b

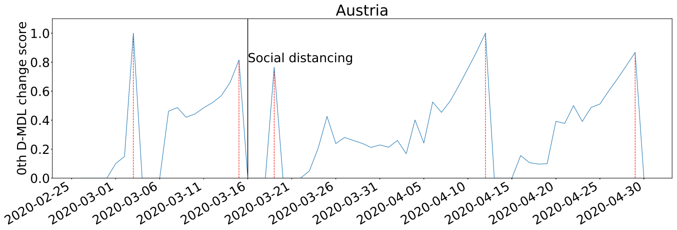

c

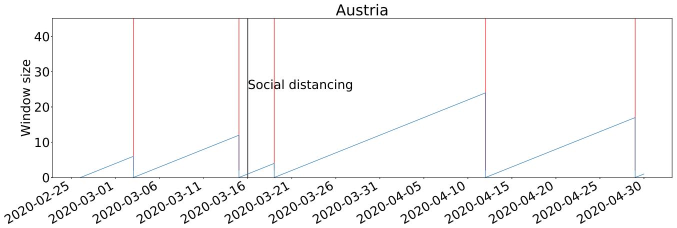

d

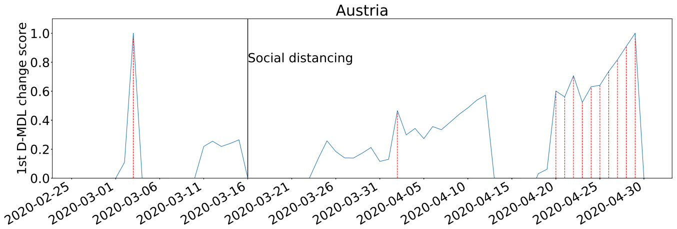

e

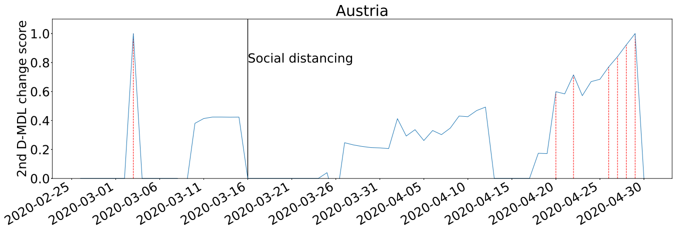

Fig. 3: The results for Austria with Gaussian modeling. The date on which the social distancing was implemented is marked by a solid line in black. **a,** the number of daily new cases. **b,** the change scores produced by the 0th M-DML where the line in blue denotes values of scores and dashed lines in red mark alarms. **c,** the window sized for the sequential D-DML algorithm with adaptive window where lines in red mark the shrinkage of windows. **d,** the change scores produced by the 1st D-MDL. **e,** the change scores produced by the 2nd D-MDL.

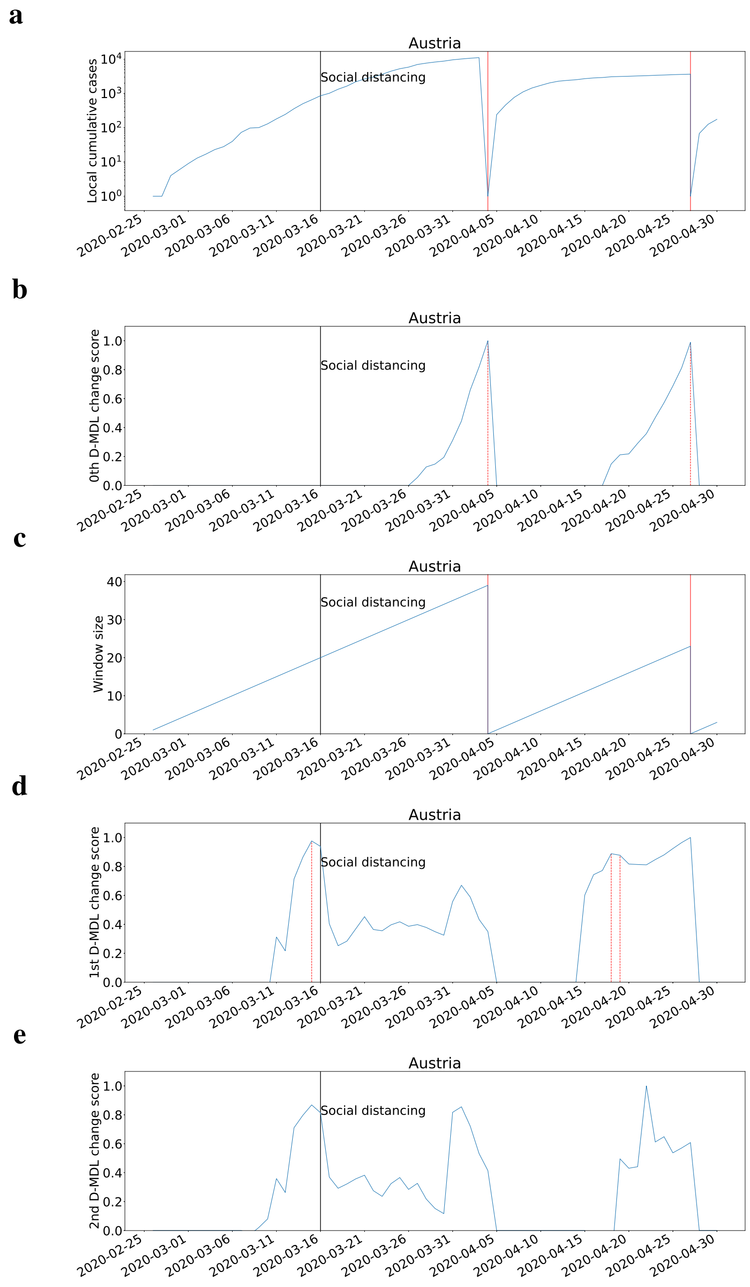

Fig. 4: The results for Austria with exponential modeling. The date on which the social distancing was implemented is marked by a solid line in black. **a,** the number of cumulative cases. **b,** the change scores produced by the 0th M-DML where the line in blue denotes values of scores and dashed lines in red mark alarms. **c,** the window sized for the sequential D-DML algorithm with adaptive window where lines in red mark the shrinkage of windows. **d,** the change scores produced by the 1st D-MDL. **e,** the change scores produced by the 2nd D-MDL.

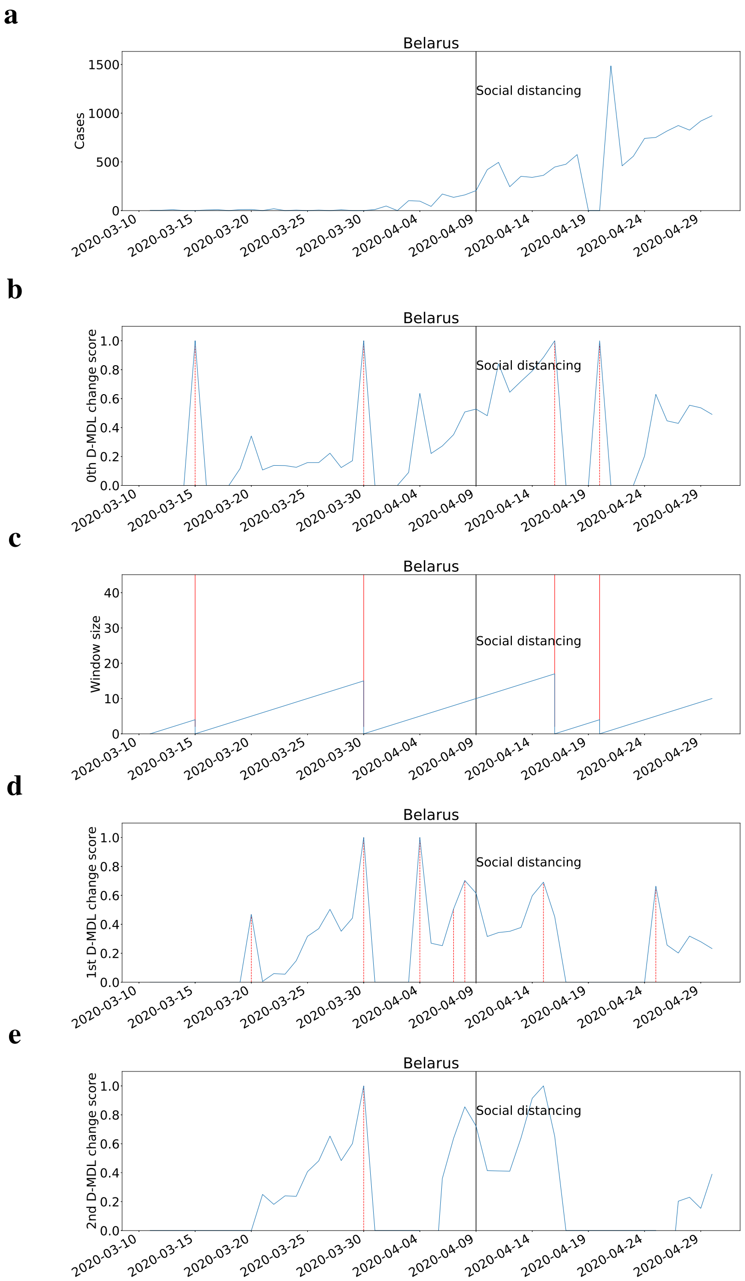

Fig. 5: The results for Belarus with Gaussian modeling. The date on which the social distancing was implemented is marked by a solid line in black. **a,** the number of daily new cases. **b,** the change scores produced by the 0th M-DML where the line in blue denotes values of scores and dashed lines in red mark alarms. **c,** the window sized for the sequential D-DML algorithm with adaptive window where lines in red mark the shrinkage of windows. **d,** the change scores produced by the 1st D-MDL. **e,** the change scores produced by the 2nd D-MDL.

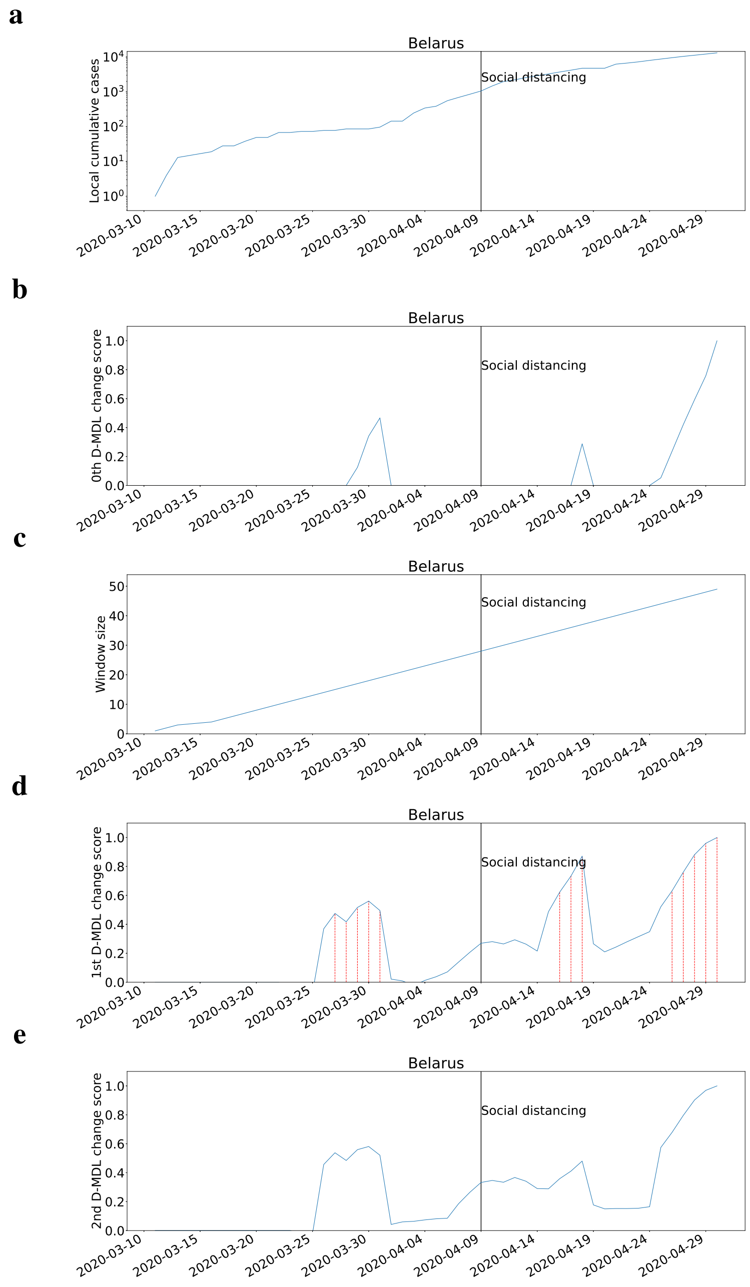

Fig. 6: The results for Belarus with exponential modeling. The date on which the social distancing was implemented is marked by a solid line in black. **a,** the number of cumulative cases. **b,** the change scores produced by the 0th M-DML where the line in blue denotes values of scores and dashed lines in red mark alarms. **c,** the window sized for the sequential D-DML algorithm with adaptive window where lines in red mark the shrinkage of windows. **d,** the change scores produced by the 1st D-MDL. **e,** the change scores produced by the 2nd D-MDL.

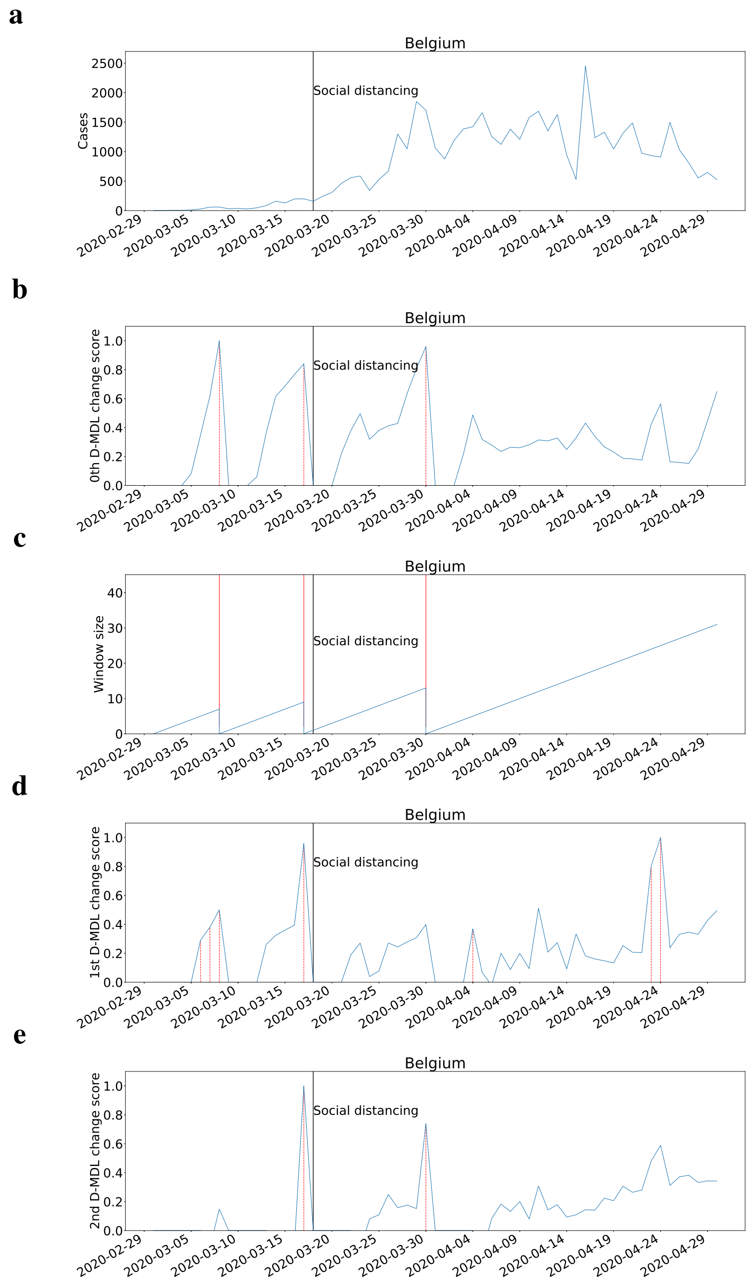

Fig. 7: The results for Belgium with Gaussian modeling. The date on which the social distancing was implemented is marked by a solid line in black. **a,** the number of daily new cases. **b,** the change scores produced by the 0th M-DML where the line in blue denotes values of scores and dashed lines in red mark alarms. **c,** the window sized for the sequential D-DML algorithm with adaptive window where lines in red mark the shrinkage of windows. **d,** the change scores produced by the 1st D-MDL. **e,** the change scores produced by the 2nd D-MDL.

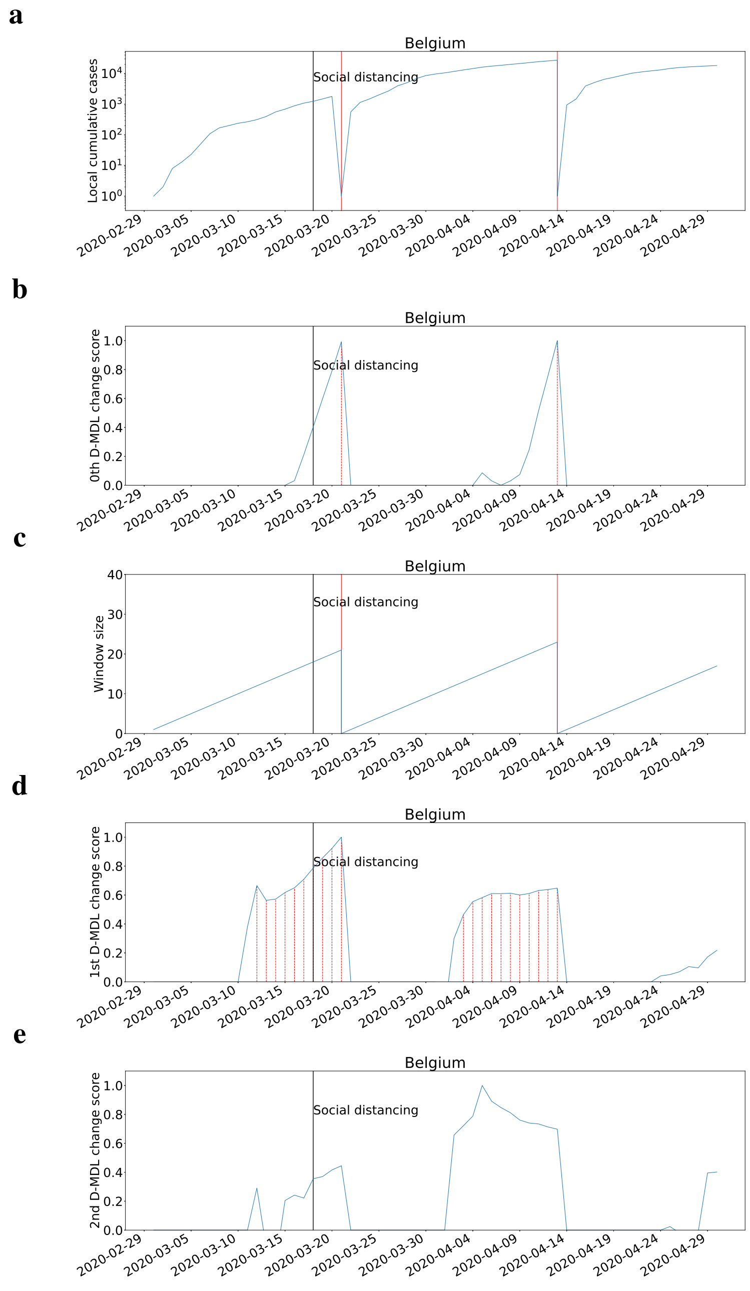

Fig. 8: The results for Belgium with exponential modeling. The date on which the social distancing was implemented is marked by a solid line in black. **a,** the number of cumulative cases. **b,** the change scores produced by the 0th M-DML where the line in blue denotes values of scores and dashed lines in red mark alarms. **c,** the window sized for the sequential D-DML algorithm with adaptive window where lines in red mark the shrinkage of windows. **d,** the change scores produced by the 1st D-MDL. **e,** the change scores produced by the 2nd D-MDL.

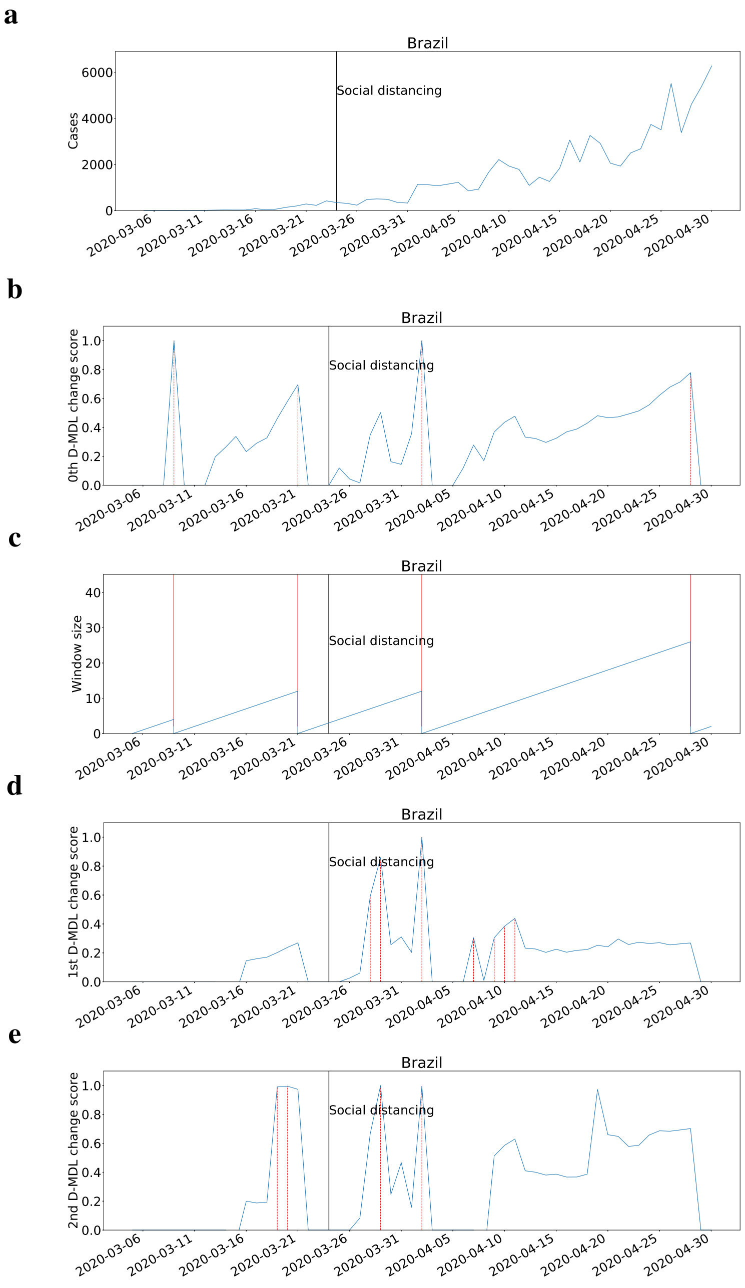

Fig. 9: The results for Brazil with Gaussian modeling. The date on which the social distancing was implemented is marked by a solid line in black. **a,** the number of daily new cases. **b,** the change scores produced by the 0th M-DML where the line in blue denotes values of scores and dashed lines in red mark alarms. **c,** the window sized for the sequential D-DML algorithm with adaptive window where lines in red mark the shrinkage of windows. **d,** the change scores produced by the 1st D-MDL. **e,** the change scores produced by the 2nd D-MDL.

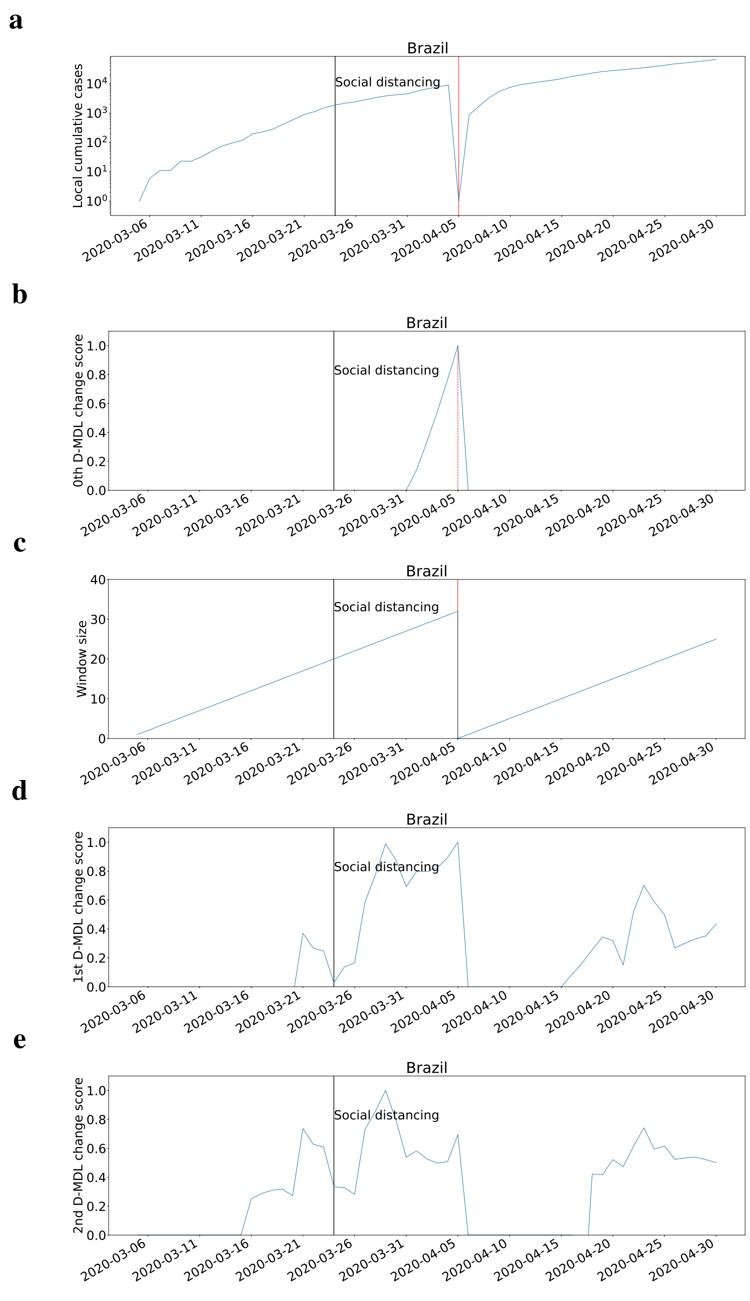

Fig. 10: The results for Brazil with exponential modeling. The date on which the social distancing was implemented is marked by a solid line in black. **a,** the number of cumulative cases. **b,** the change scores produced by the 0th M-DML where the line in blue denotes values of scores and dashed lines in red mark alarms. **c,** the window sized for the sequential D-DML algorithm with adaptive window where lines in red mark the shrinkage of windows. **d,** the change scores produced by the 1st D-MDL. **e,** the change scores produced by the 2nd D-MDL.

a

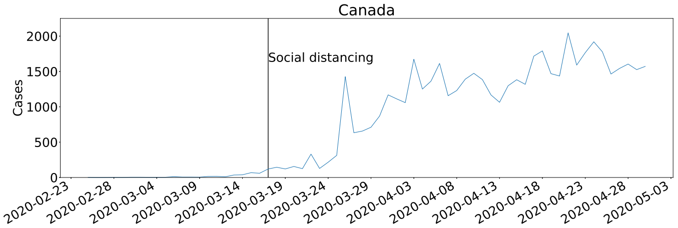

b

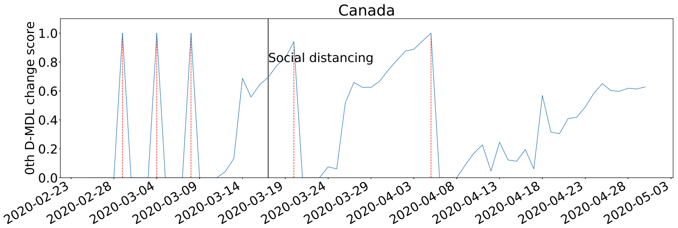

c

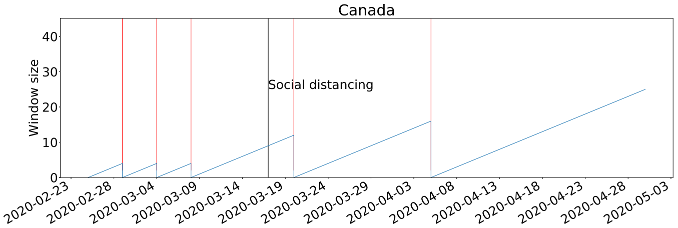

d

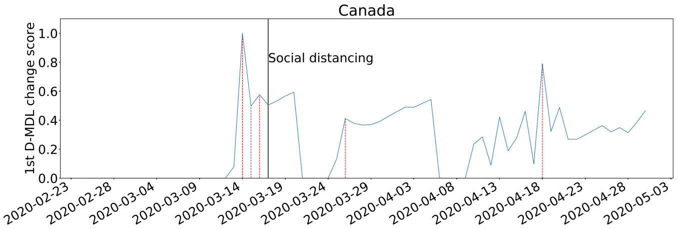

e

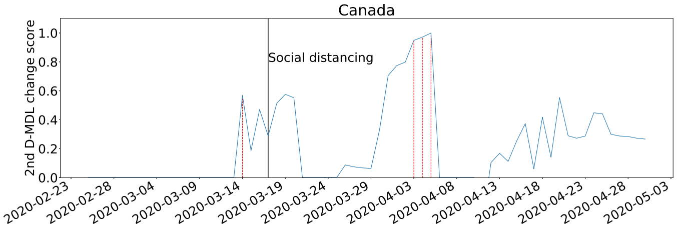

Fig. 11: The results for Canada with Gaussian modeling. The date on which the social distancing was implemented is marked by a solid line in black. **a,** the number of daily new cases. **b,** the change scores produced by the 0th M-DML where the line in blue denotes values of scores and dashed lines in red mark alarms. **c,** the window sized for the sequential D-DML algorithm with adaptive window where lines in red mark the shrinkage of windows. **d,** the change scores produced by the 1st D-MDL. **e,** the change scores produced by the 2nd D-MDL.

a

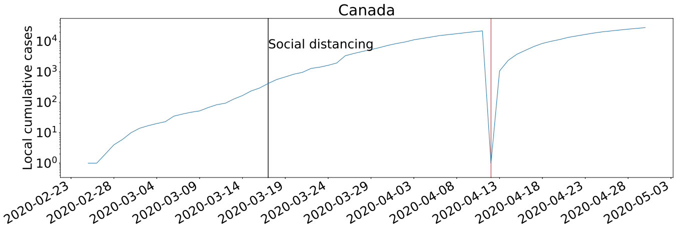

b

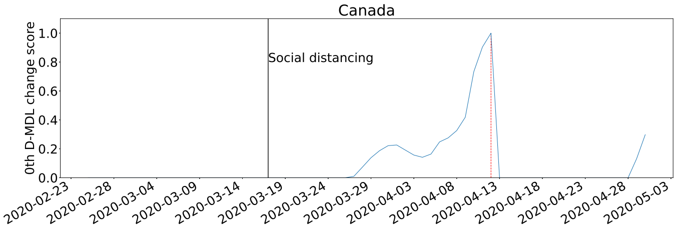

c

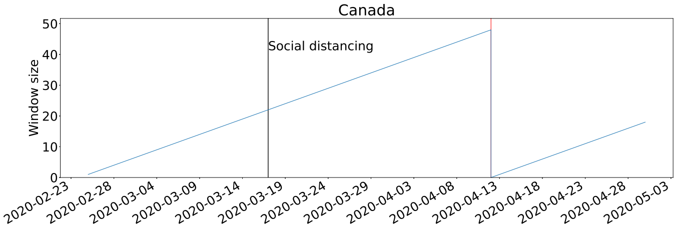

d

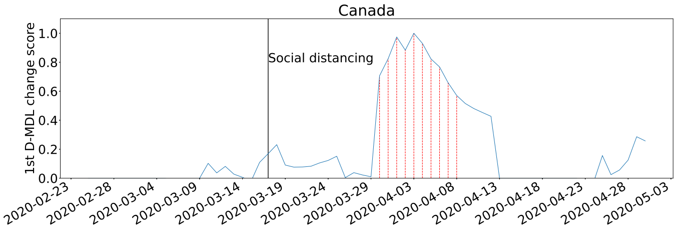

e

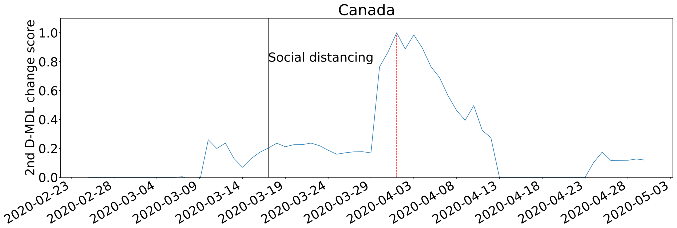

Fig. 12: The results for Canada with exponential modeling. The date on which the social distancing was implemented is marked by a solid line in black. **a,** the number of cumulative cases. **b,** the change scores produced by the 0th M-DML where the line in blue denotes values of scores and dashed lines in red mark alarms. **c,** the window sized for the sequential D-DML algorithm with adaptive window where lines in red mark the shrinkage of windows. **d,** the change scores produced by the 1st D-MDL. **e,** the change scores produced by the 2nd D-MDL.

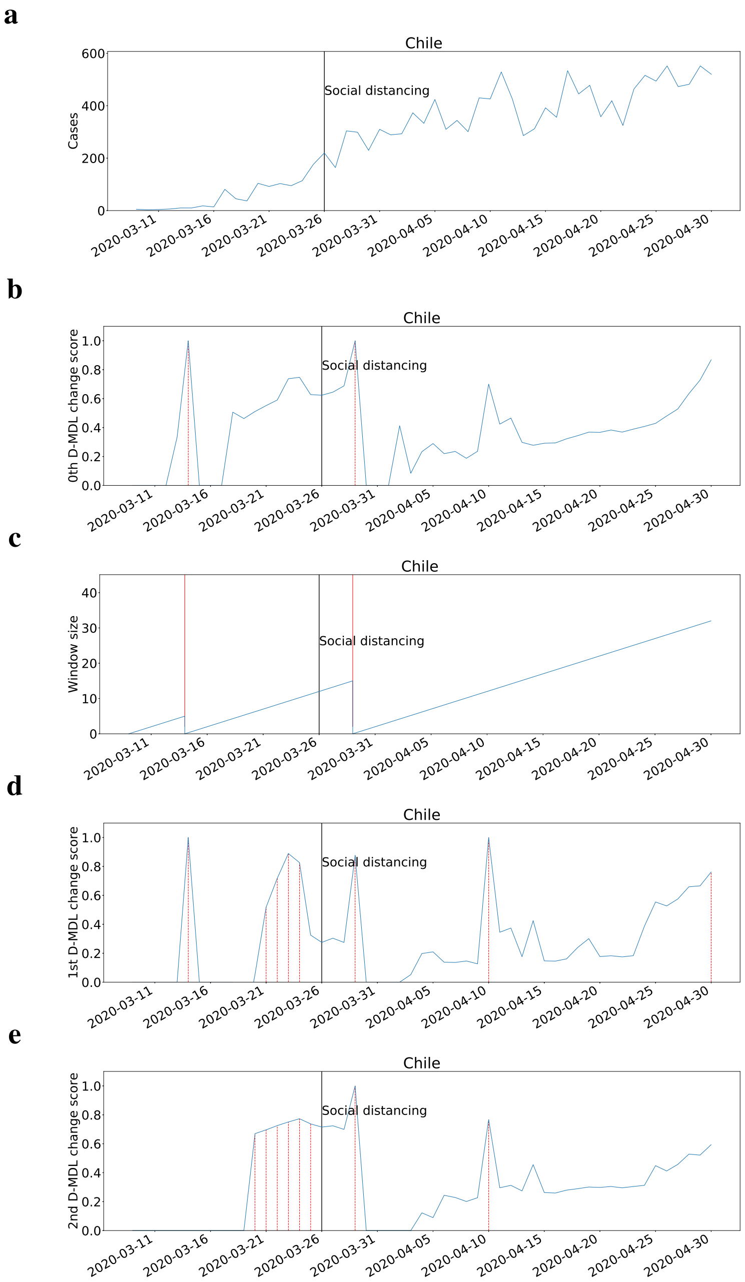

Fig. 13: The results for Chile with Gaussian modeling. The date on which the social distancing was implemented is marked by a solid line in black. **a,** the number of daily new cases. **b,** the change scores produced by the 0th M-DML where the line in blue denotes values of scores and dashed lines in red mark alarms. **c,** the window sized for the sequential D-DML algorithm with adaptive window where lines in red mark the shrinkage of windows. **d,** the change scores produced by the 1st D-MDL. **e,** the change scores produced by the 2nd D-MDL.

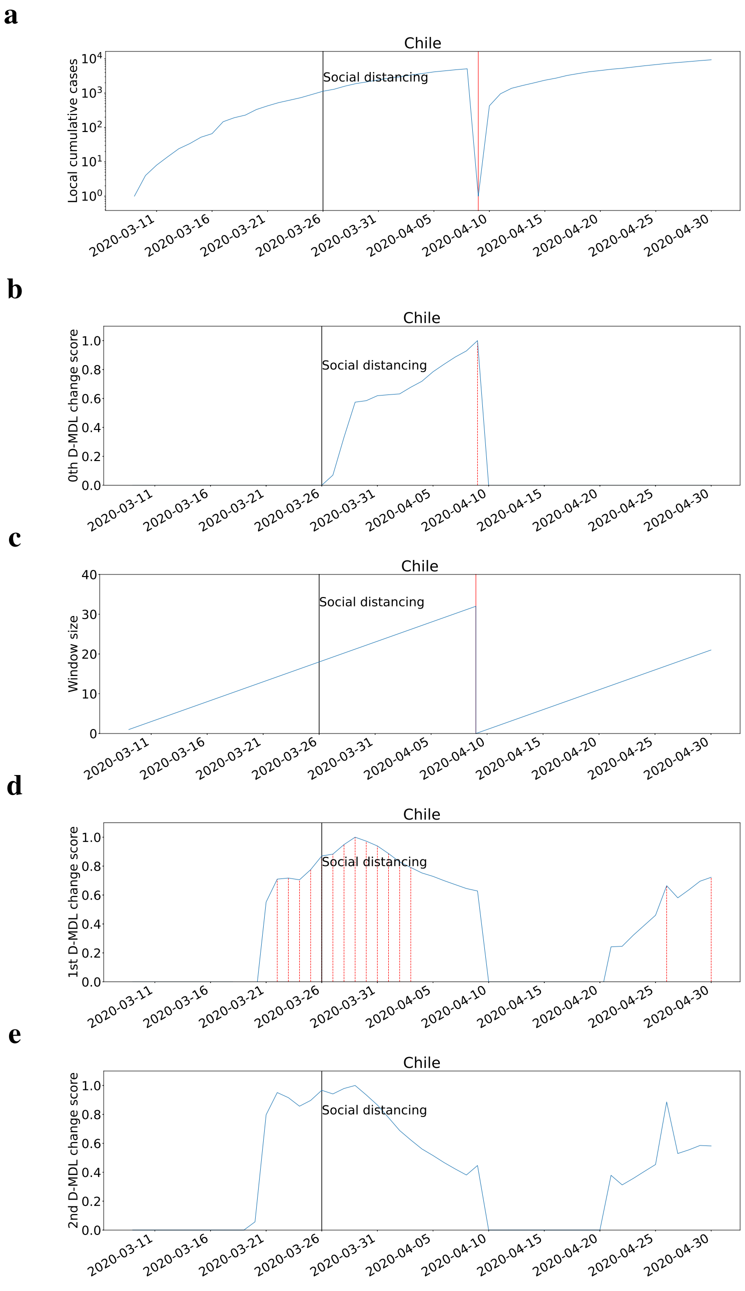

Fig. 14: The results for Chile with exponential modeling. The date on which the social distancing was implemented is marked by a solid line in black. **a,** the number of cumulative cases. **b,** the change scores produced by the 0th M-DML where the line in blue denotes values of scores and dashed lines in red mark alarms. **c,** the window sized for the sequential D-DML algorithm with adaptive window where lines in red mark the shrinkage of windows. **d,** the change scores produced by the 1st D-MDL. **e,** the change scores produced by the 2nd D-MDL.

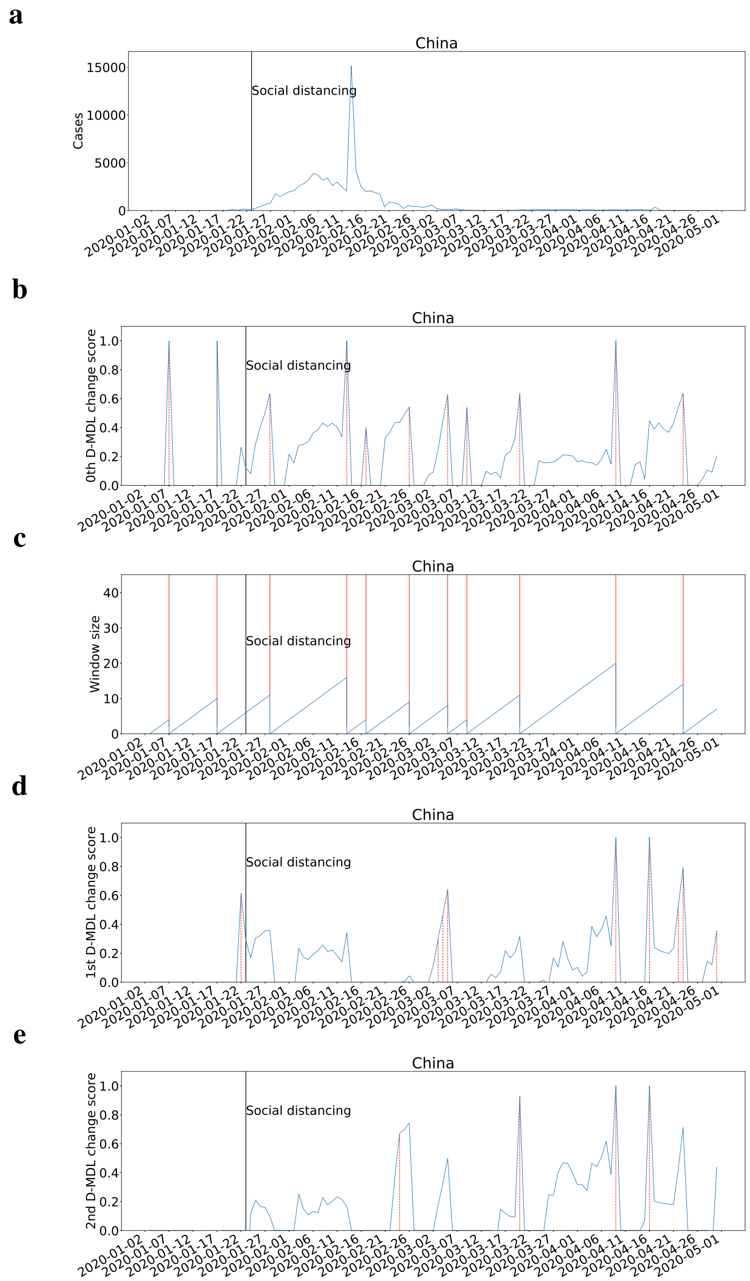

Fig. 15: The results for China with Gaussian modeling. The date on which the social distancing was implemented is marked by a solid line in black. **a,** the number of daily new cases. **b,** the change scores produced by the 0th M-DML where the line in blue denotes values of scores and dashed lines in red mark alarms. **c,** the window sized for the sequential D-DML algorithm with adaptive window where lines in red mark the shrinkage of windows. **d,** the change scores produced by the 1st D-MDL. **e,** the change scores produced by the 2nd D-MDL.

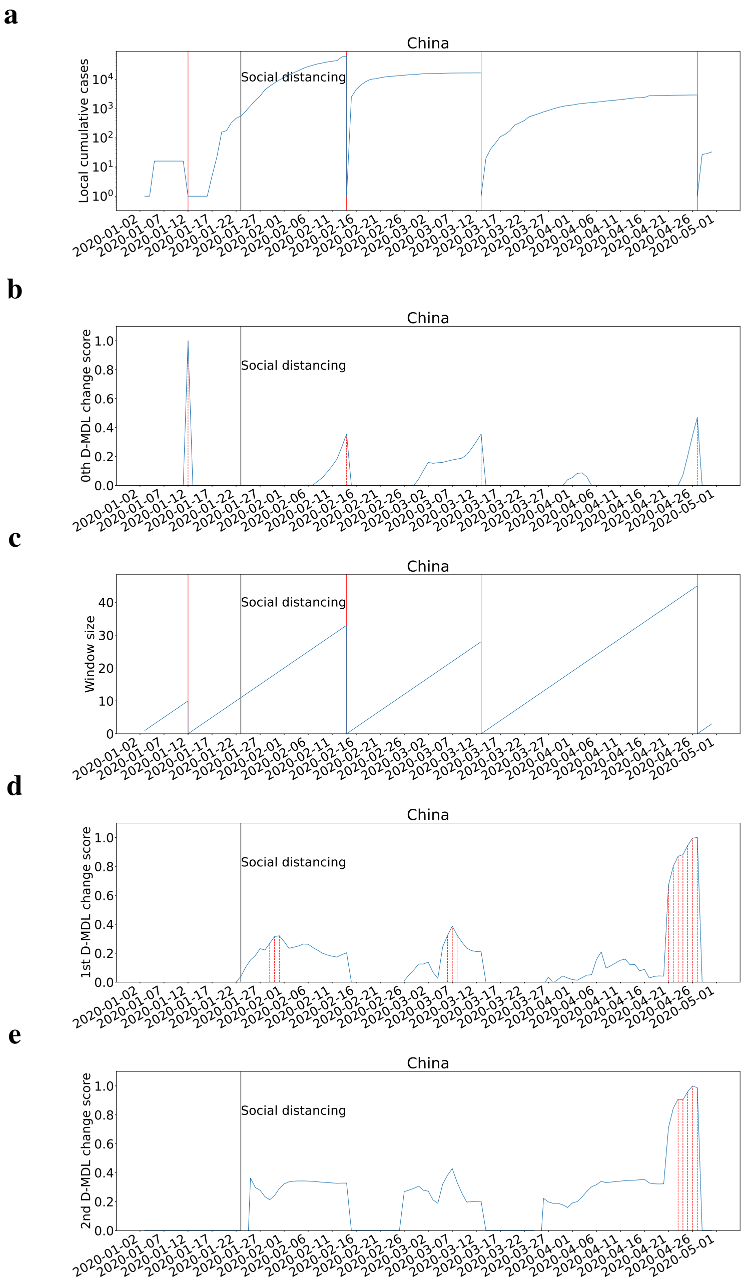

Fig. 16: The results for China with exponential modeling. The date on which the social distancing was implemented is marked by a solid line in black. **a,** the number of cumulative cases. **b,** the change scores produced by the 0th M-DML where the line in blue denotes values of scores and dashed lines in red mark alarms. **c,** the window sized for the sequential D-DML algorithm with adaptive window where lines in red mark the shrinkage of windows. **d,** the change scores produced by the 1st D-MDL. **e,** the change scores produced by the 2nd D-MDL.

a

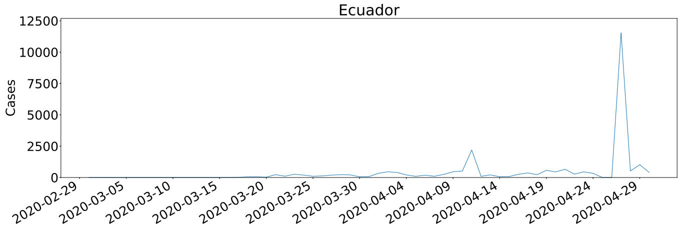

b

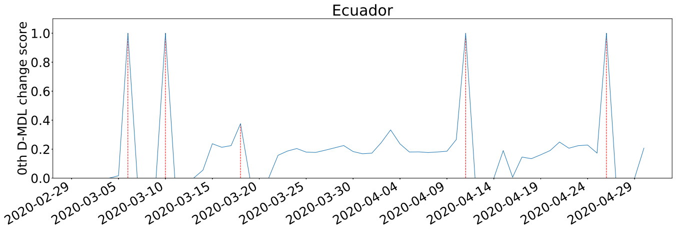

c

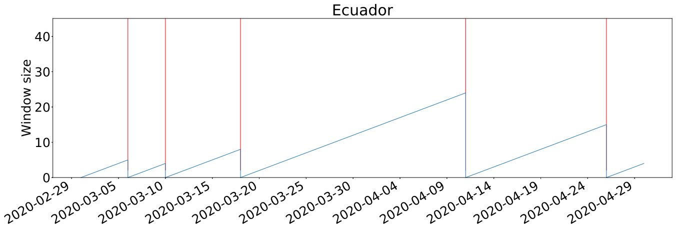

d

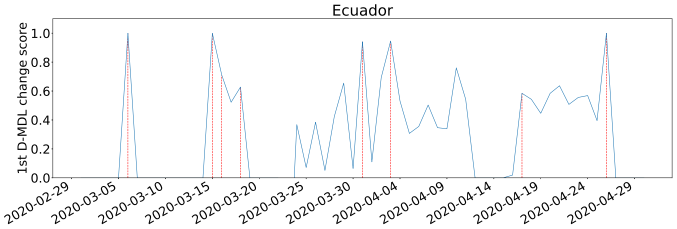

e

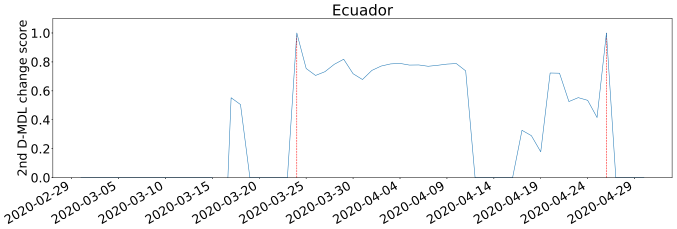

Fig. 17: The results for Ecuador with Gaussian modeling. The date on which the social distancing was implemented is marked by a solid line in black. **a,** the number of daily new cases. **b,** the change scores produced by the 0th M-DML where the line in blue denotes values of scores and dashed lines in red mark alarms. **c,** the window sized for the sequential D-DML algorithm with adaptive window where lines in red mark the shrinkage of windows. **d,** the change scores produced by the 1st D-MDL. **e,** the change scores produced by the 2nd D-MDL.

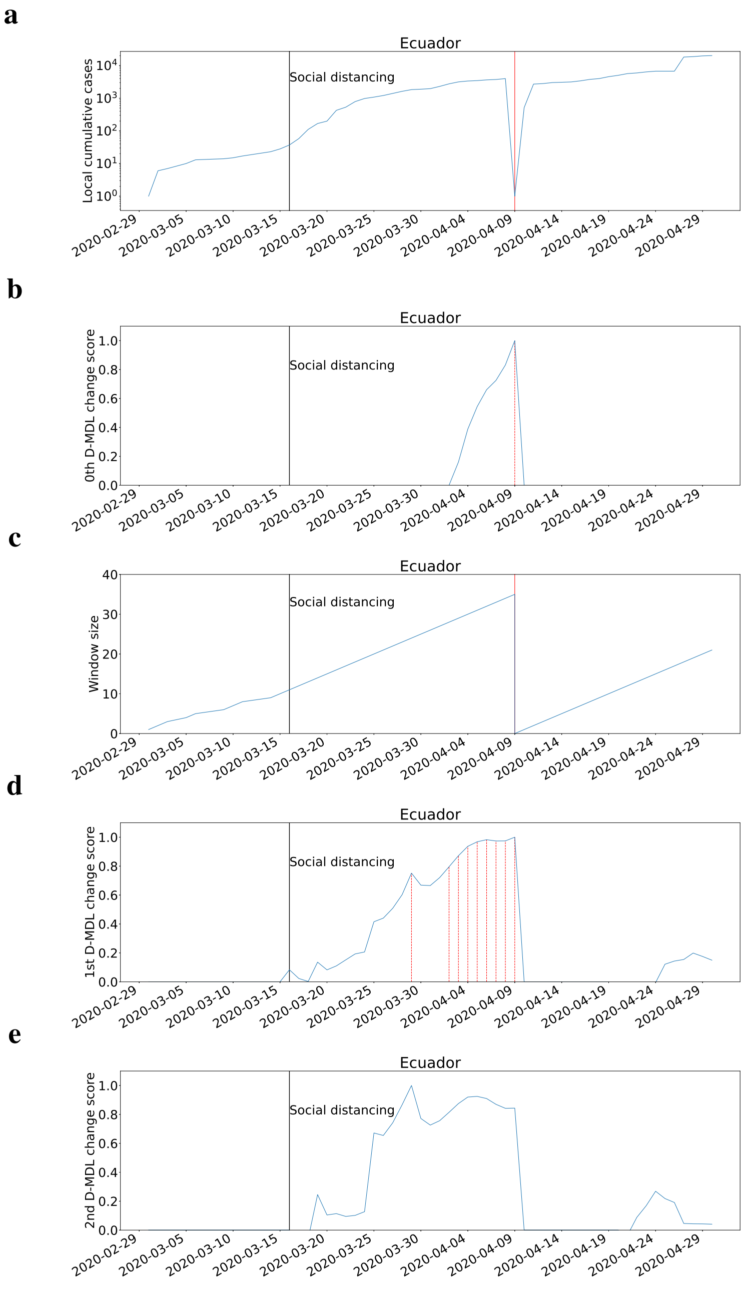

Fig. 18: The results for Ecuador with exponential modeling. The date on which the social distancing was implemented is marked by a solid line in black. **a,** the number of cumulative cases. **b,** the change scores produced by the 0th M-DML where the line in blue denotes values of scores and dashed lines in red mark alarms. **c,** the window sized for the sequential D-DML algorithm with adaptive window where lines in red mark the shrinkage of windows. **d,** the change scores produced by the 1st D-MDL. **e,** the change scores produced by the 2nd D-MDL.

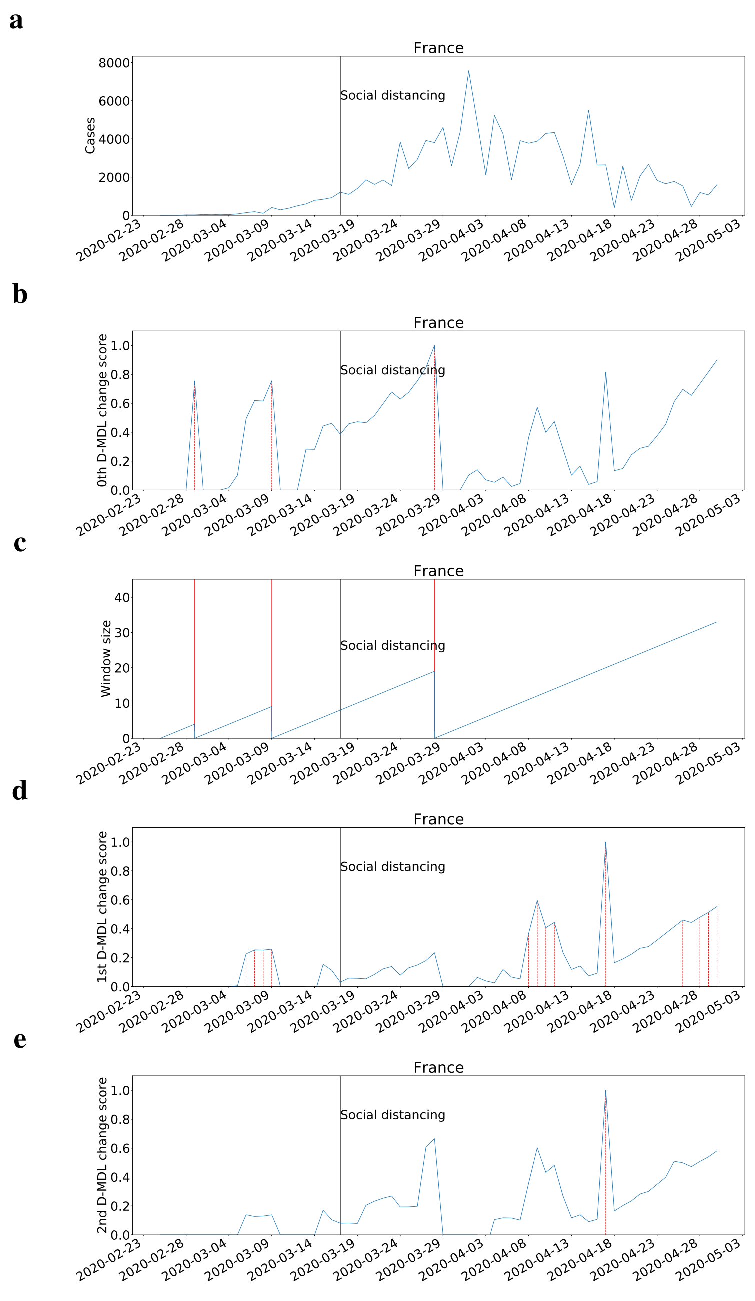

Fig. 19: The results for France with Gaussian modeling. The date on which the social distancing was implemented is marked by a solid line in black. **a,** the number of daily new cases. **b,** the change scores produced by the 0th M-DML where the line in blue denotes values of scores and dashed lines in red mark alarms. **c,** the window sized for the sequential D-DML algorithm with adaptive window where lines in red mark the shrinkage of windows. **d,** the change scores produced by the 1st D-MDL. **e,** the change scores produced by the 2nd D-MDL.

a

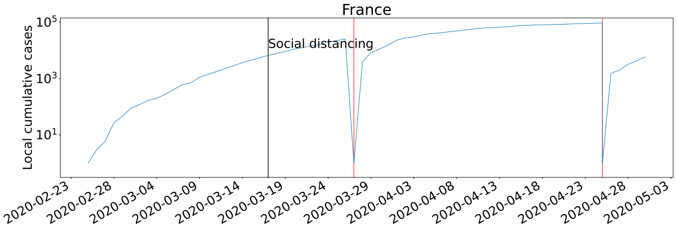

b

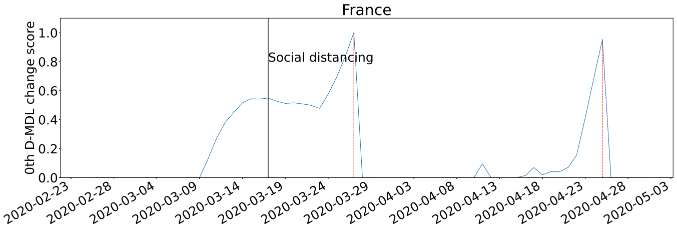

c

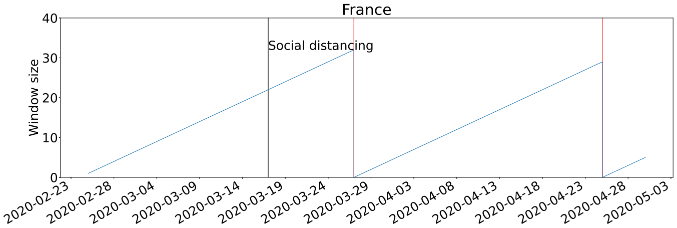

d

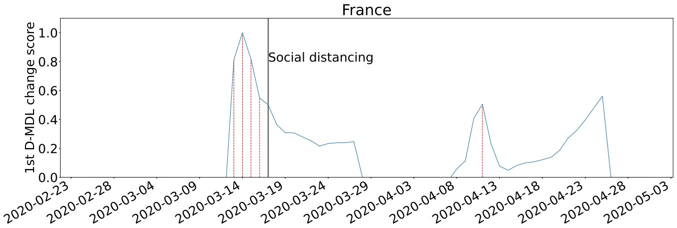

e

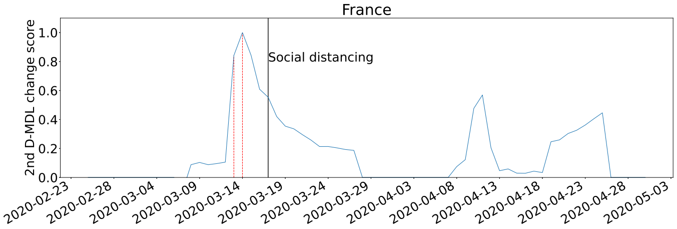

Fig. 20: The results for France with exponential modeling. The date on which the social distancing was implemented is marked by a solid line in black. **a,** the number of cumulative cases. **b,** the change scores produced by the 0th M-DML where the line in blue denotes values of scores and dashed lines in red mark alarms. **c,** the window sized for the sequential D-DML algorithm with adaptive window where lines in red mark the shrinkage of windows. **d,** the change scores produced by the 1st D-MDL. **e,** the change scores produced by the 2nd D-MDL.

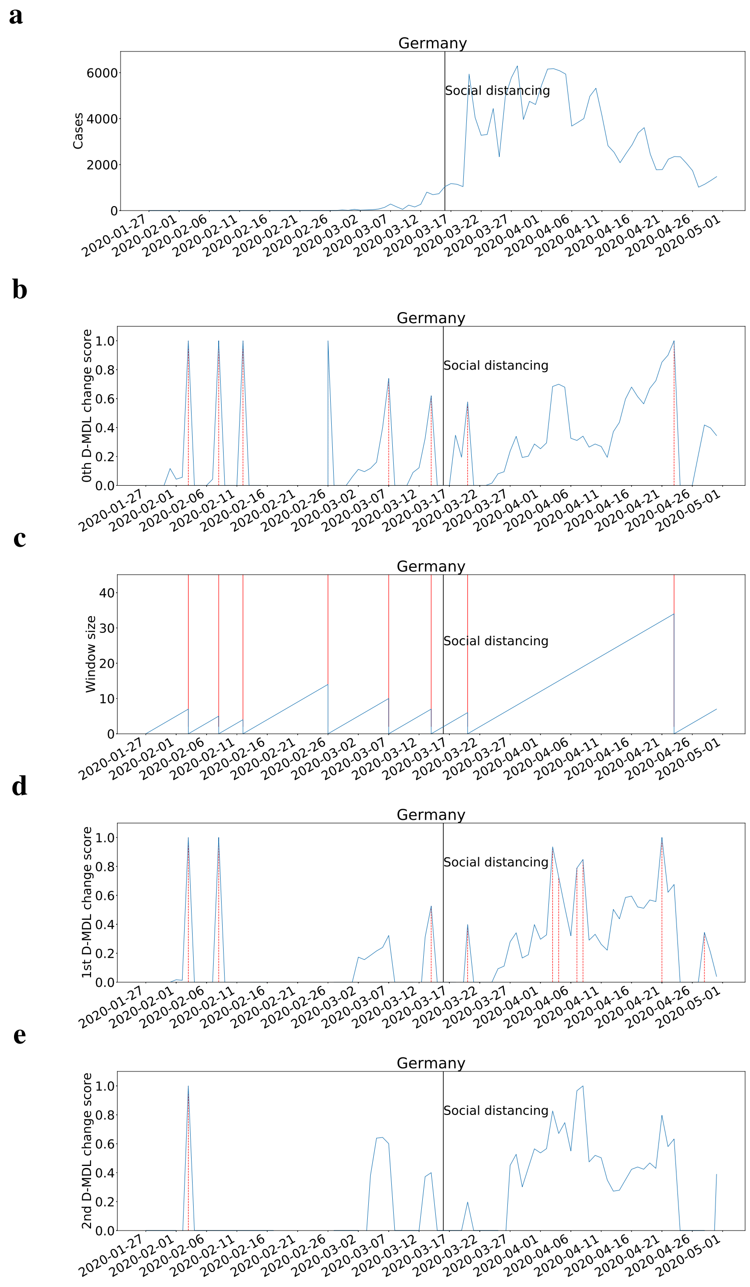

Fig. 21: The results for Germany with Gaussian modeling. The date on which the social distancing was implemented is marked by a solid line in black. **a,** the number of daily new cases. **b,** the change scores produced by the 0th M-DML where the line in blue denotes values of scores and dashed lines in red mark alarms. **c,** the window sized for the sequential D-DML algorithm with adaptive window where lines in red mark the shrinkage of windows. **d,** the change scores produced by the 1st D-MDL. **e,** the change scores produced by the 2nd D-MDL.

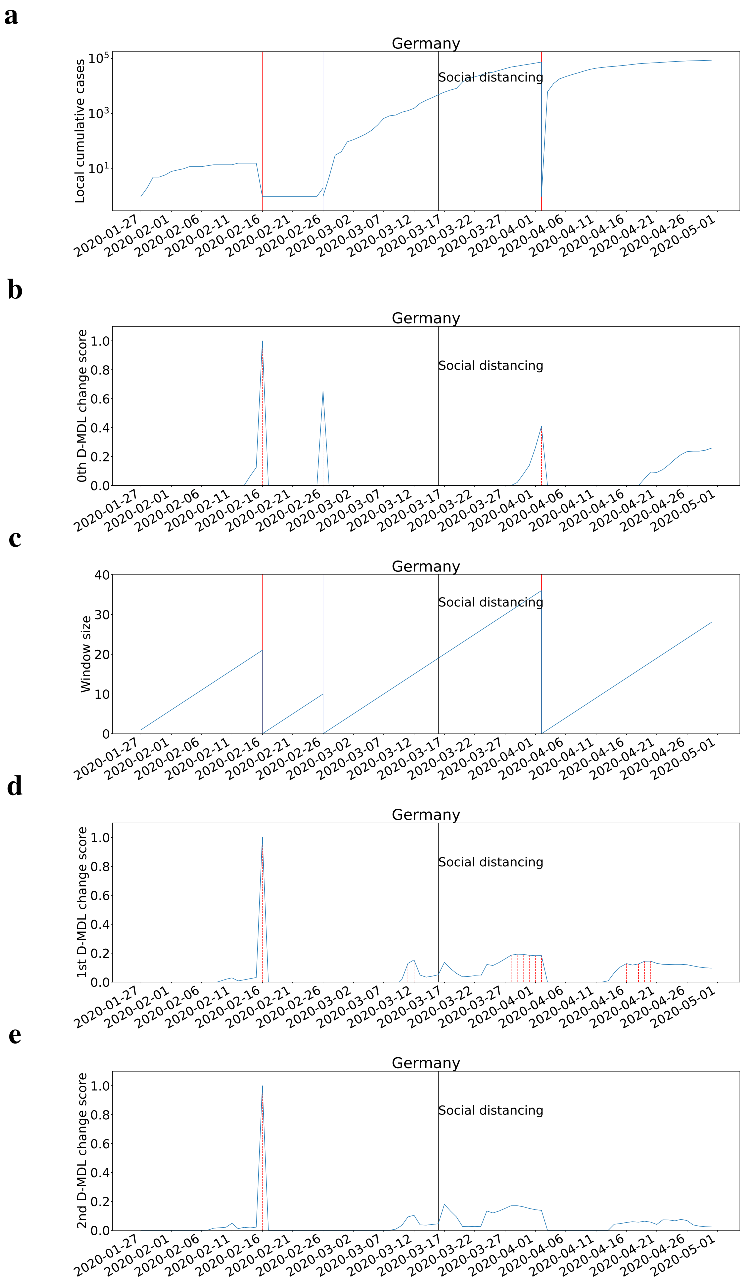

Fig. 22: The results for Germany with exponential modeling. The date on which the social distancing was implemented is marked by a solid line in black. **a,** the number of cumulative cases. **b,** the change scores produced by the 0th M-DML where the line in blue denotes values of scores and dashed lines in red mark alarms. **c,** the window sized for the sequential D-DML algorithm with adaptive window where lines in red mark the shrinkage of windows. **d,** the change scores produced by the 1st D-MDL. **e,** the change scores produced by the 2nd D-MDL.

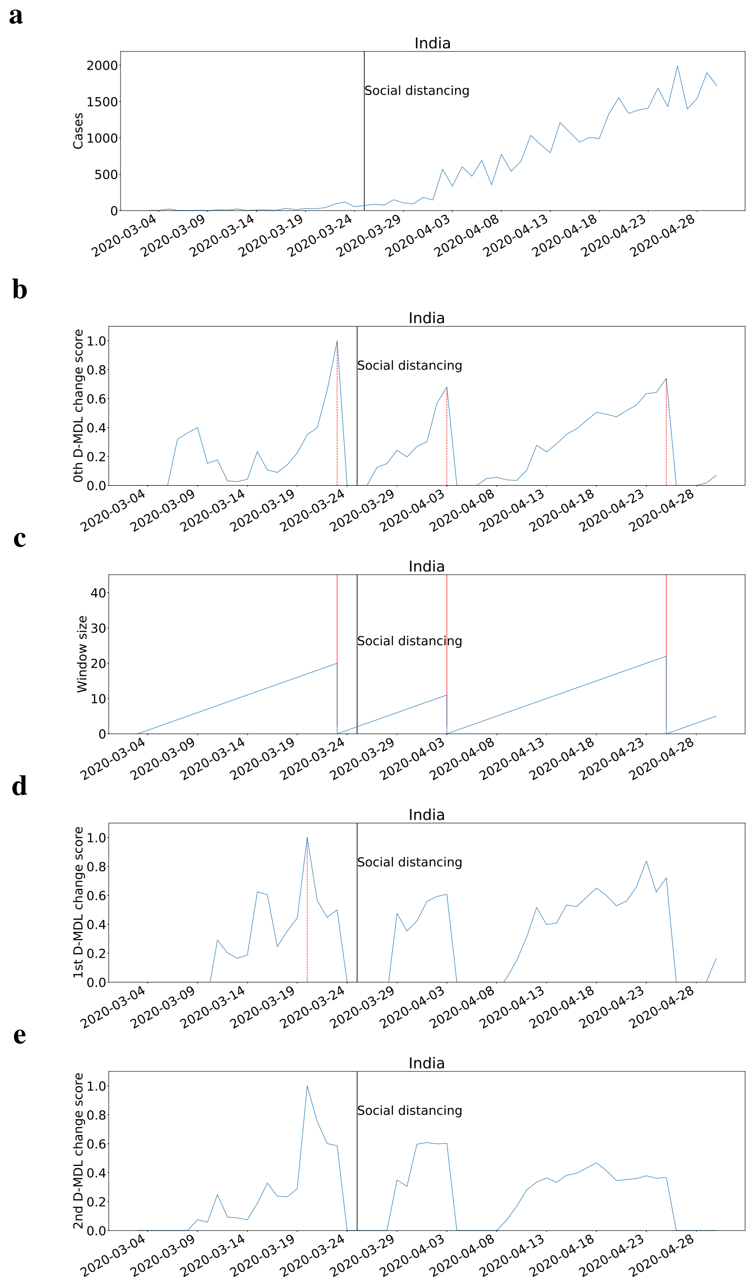

Fig. 23: The results for India with Gaussian modeling. The date on which the social distancing was implemented is marked by a solid line in black. **a,** the number of daily new cases. **b,** the change scores produced by the 0th M-DML where the line in blue denotes values of scores and dashed lines in red mark alarms. **c,** the window sized for the sequential D-DML algorithm with adaptive window where lines in red mark the shrinkage of windows. **d,** the change scores produced by the 1st D-MDL. **e,** the change scores produced by the 2nd D-MDL.

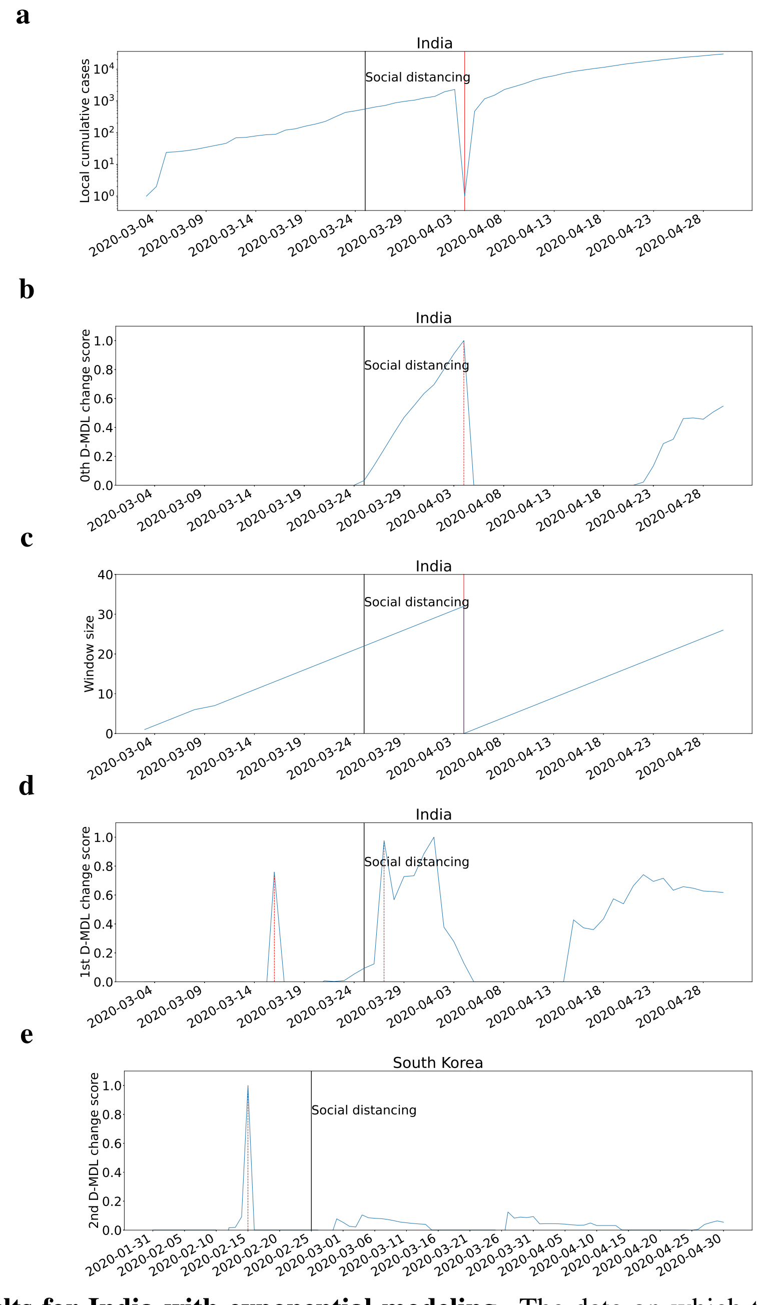

Fig. 24: The results for India with exponential modeling. The date on which the social distancing was implemented is marked by a solid line in black. **a,** the number of cumulative cases. **b,** the change scores produced by the 0th M-DML where the line in blue denotes values of scores and dashed lines in red mark alarms. **c,** the window sized for the sequential D-DML algorithm with adaptive window where lines in red mark the shrinkage of windows. **d,** the change scores produced by the 1st D-MDL. **e,** the change scores produced by the 2nd D-MDL.

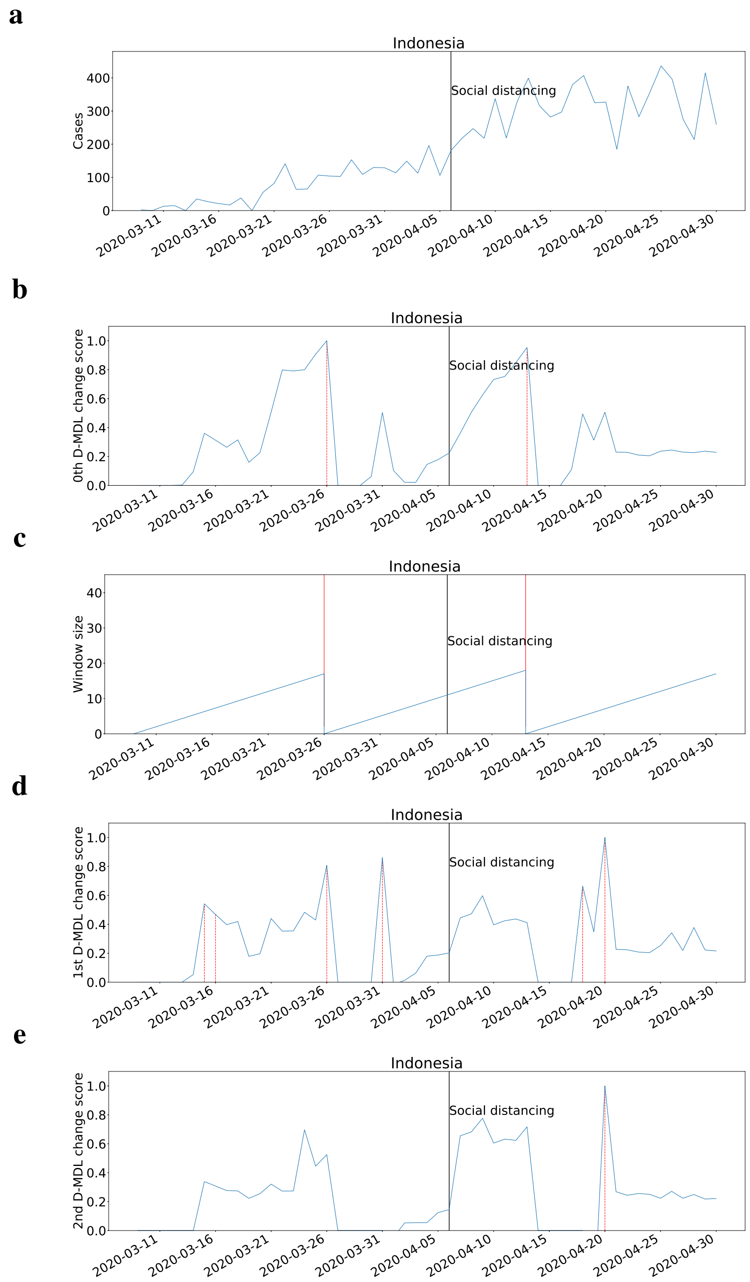

Fig. 25: The results for Indonesia with Gaussian modeling. The date on which the social distancing was implemented is marked by a solid line in black. **a,** the number of daily new cases. **b,** the change scores produced by the 0th M-DML where the line in blue denotes values of scores and dashed lines in red mark alarms. **c,** the window sized for the sequential D-DML algorithm with adaptive window where lines in red mark the shrinkage of windows. **d,** the change scores produced by the 1st D-MDL. **e,** the change scores produced by the 2nd D-MDL.

a

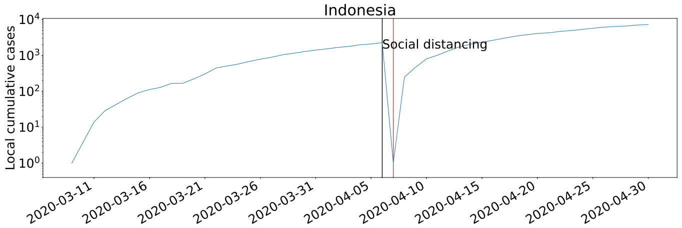

b

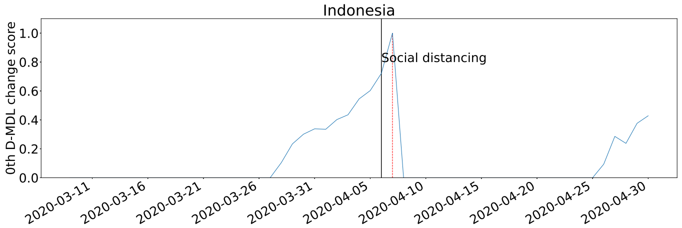

c

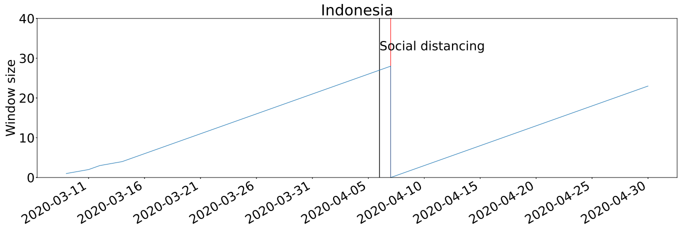

d

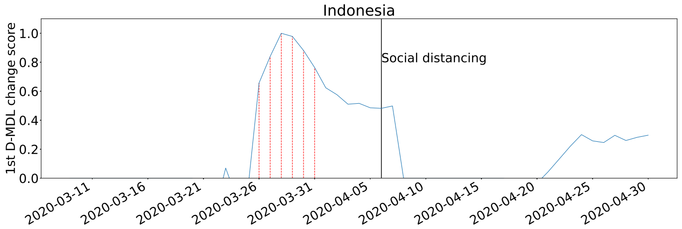

e

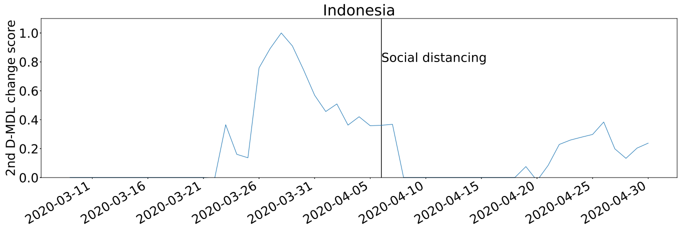

Fig. 26: The results for Indonesia with exponential modeling. The date on which the social distancing was implemented is marked by a solid line in black. **a,** the number of cumulative cases. **b,** the change scores produced by the 0th M-DML where the line in blue denotes values of scores and dashed lines in red mark alarms. **c,** the window sized for the sequential D-DML algorithm with adaptive window where lines in red mark the shrinkage of windows. **d,** the change scores produced by the 1st D-MDL. **e,** the change scores produced by the 2nd D-MDL.

a

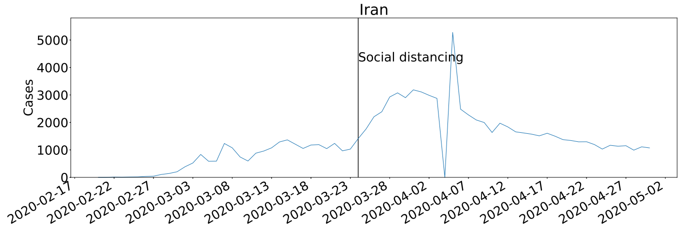

b

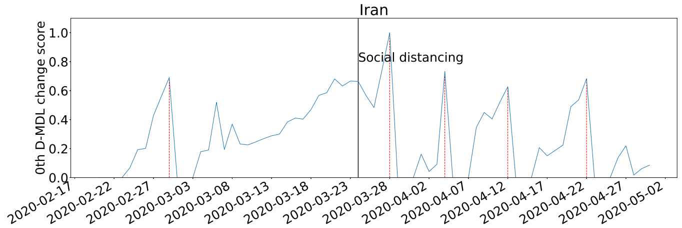

c

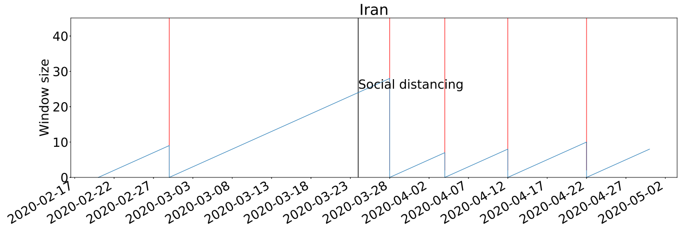

d

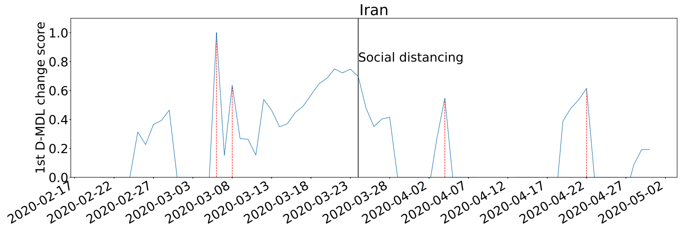

e

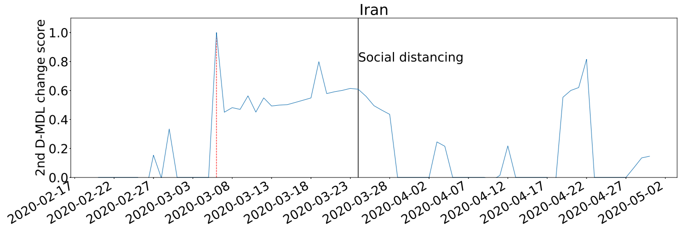

Fig. 27: The results for Iran with Gaussian modeling. The date on which the social distancing was implemented is marked by a solid line in black. **a,** the number of daily new cases. **b,** the change scores produced by the 0th M-DML where the line in blue denotes values of scores and dashed lines in red mark alarms. **c,** the window sized for the sequential D-DML algorithm with adaptive window where lines in red mark the shrinkage of windows. **d,** the change scores produced by the 1st D-MDL. **e,** the change scores produced by the 2nd D-MDL.

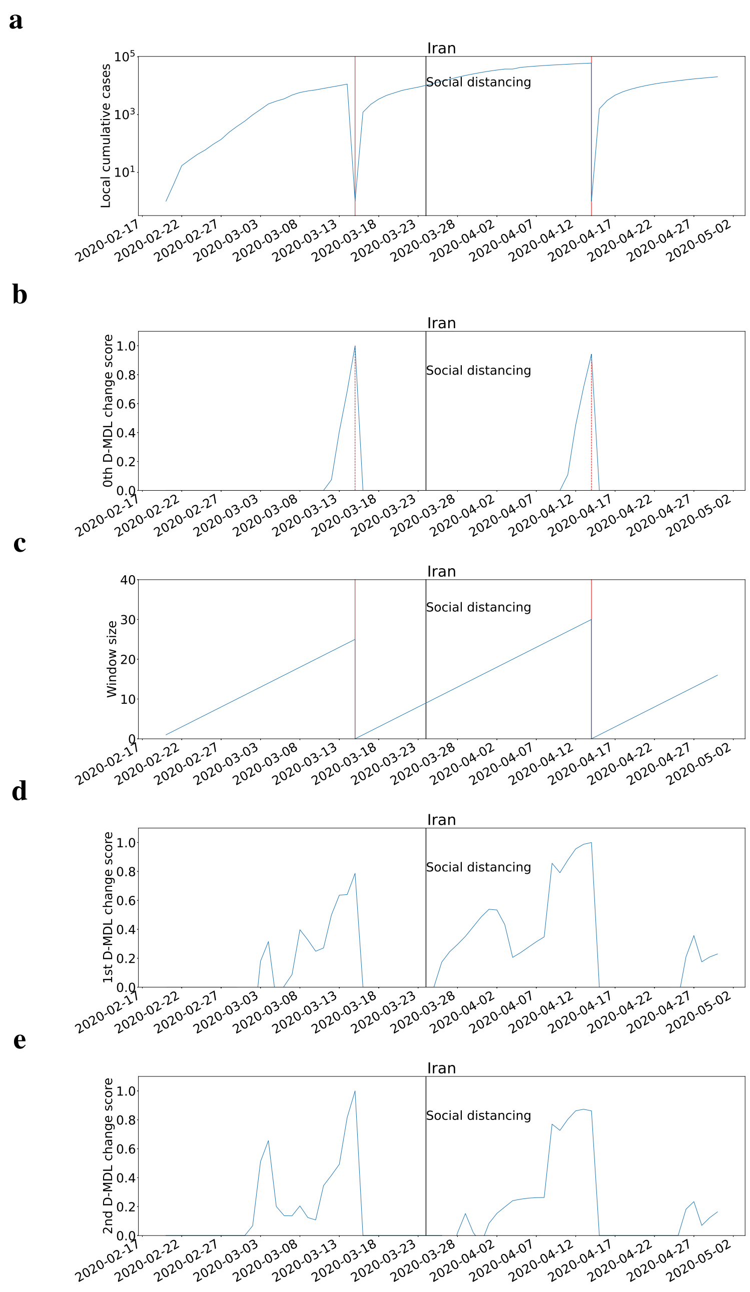

Fig. 28: The results for Iran with exponential modeling. The date on which the social distancing was implemented is marked by a solid line in black. **a,** the number of cumulative cases. **b,** the change scores produced by the 0th M-DML where the line in blue denotes values of scores and dashed lines in red mark alarms. **c,** the window sized for the sequential D-DML algorithm with adaptive window where lines in red mark the shrinkage of windows. **d,** the change scores produced by the 1st D-MDL. **e,** the change scores produced by the 2nd D-MDL.

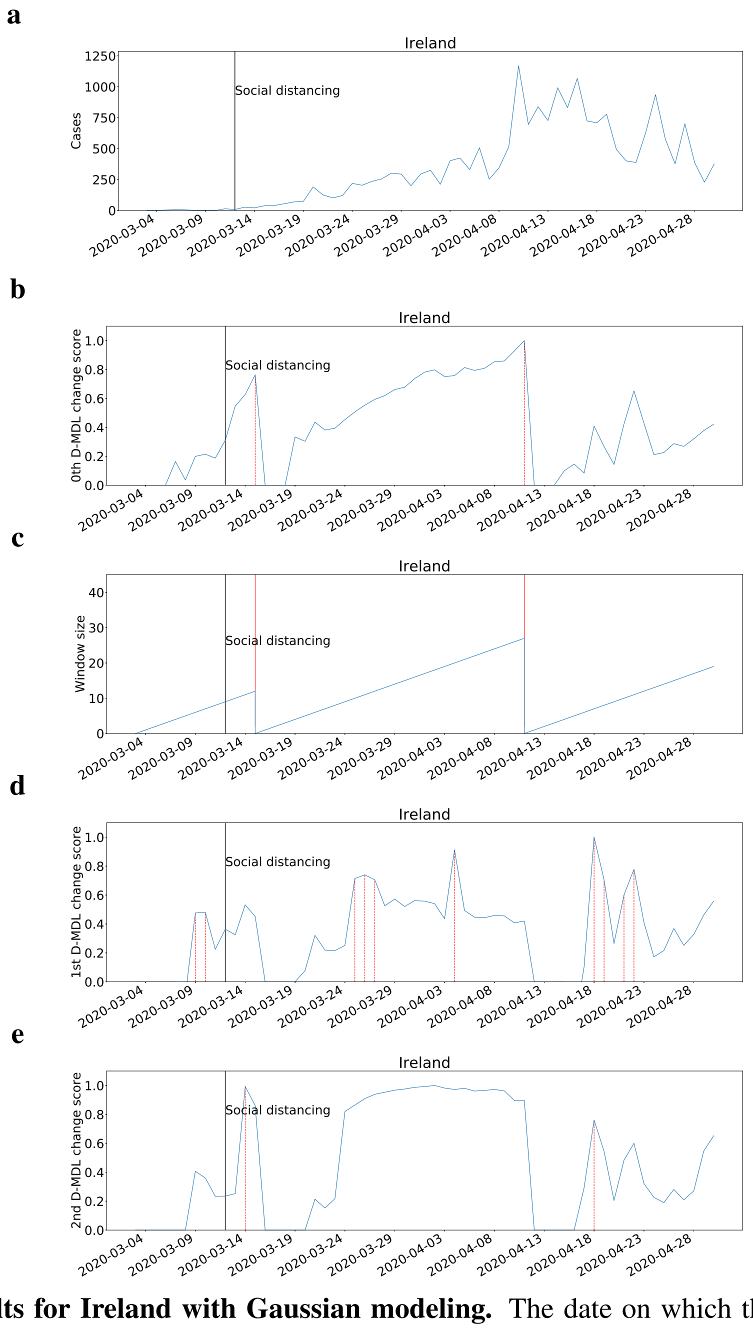

Fig. 29: The results for Ireland with Gaussian modeling. The date on which the social distancing was implemented is marked by a solid line in black. **a,** the number of daily new cases. **b,** the change scores produced by the 0th M-DML where the line in blue denotes values of scores and dashed lines in red mark alarms. **c,** the window sized for the sequential D-DML algorithm with adaptive window where lines in red mark the shrinkage of windows. **d,** the change scores produced by the 1st D-MDL. **e,** the change scores produced by the 2nd D-MDL.

a

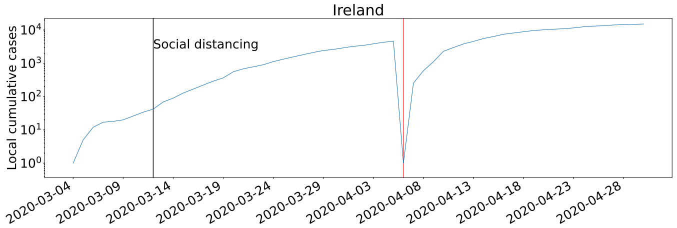

b

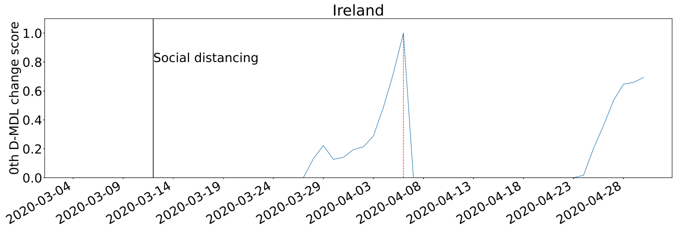

c

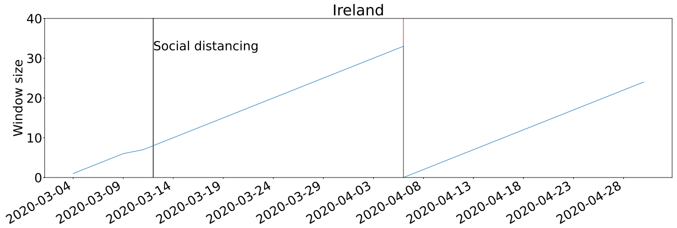

d

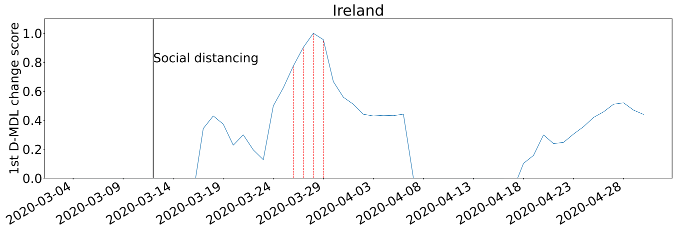

e

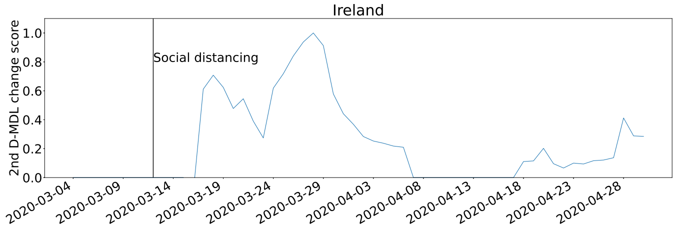

Fig. 30: The results for Ireland with exponential modeling. The date on which the social distancing was implemented is marked by a solid line in black. **a,** the number of cumulative cases. **b,** the change scores produced by the 0th M-DML where the line in blue denotes values of scores and dashed lines in red mark alarms. **c,** the window sized for the sequential D-DML algorithm with adaptive window where lines in red mark the shrinkage of windows. **d,** the change scores produced by the 1st D-MDL. **e,** the change scores produced by the 2nd D-MDL.

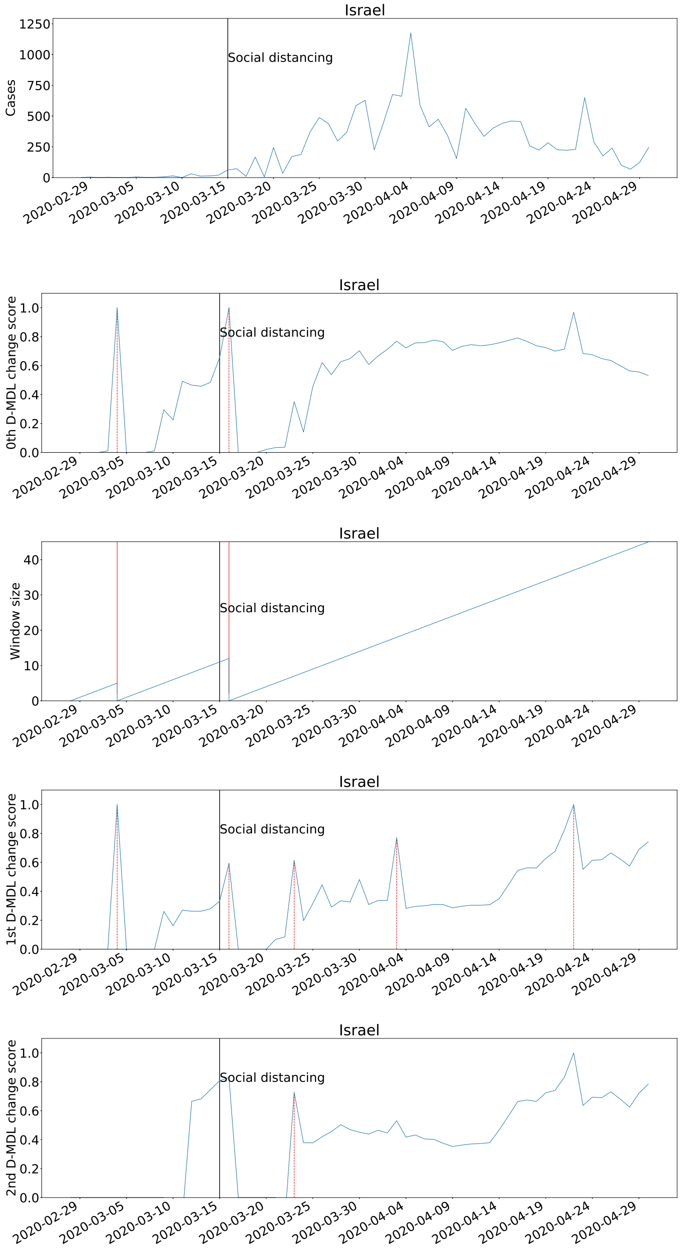

Fig. 31: The results for Israel with Gaussian modeling. The date on which the social distancing was implemented is marked by a solid line in black. **a,** the number of daily new cases. **b,** the change scores produced by the 0th M-DML where the line in blue denotes values of scores and dashed lines in red mark alarms. **c,** the window sized for the sequential D-DML algorithm with adaptive window where lines in red mark the shrinkage of windows. **d,** the change scores produced by the 1st D-MDL. **e,** the change scores produced by the 2nd D-MDL.

a

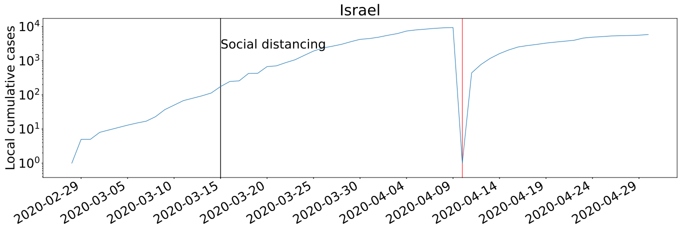

b

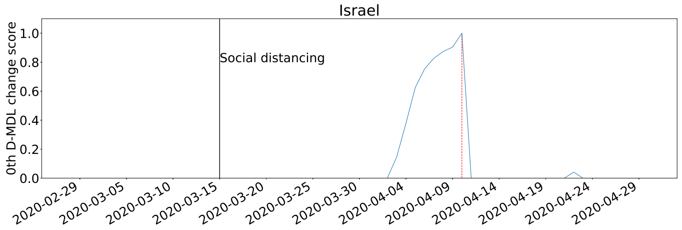

c

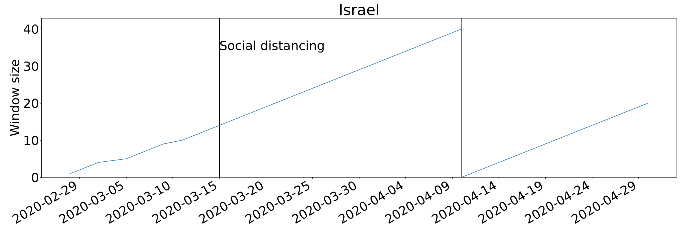

d

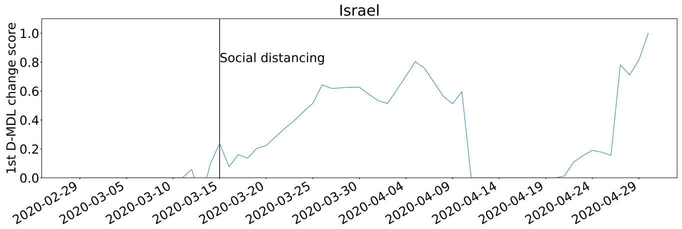

e

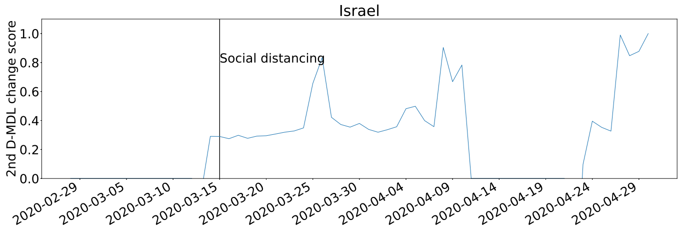

Fig. 32: The results for Israel with exponential modeling. The date on which the social distancing was implemented is marked by a solid line in black. **a,** the number of cumulative cases. **b,** the change scores produced by the 0th M-DML where the line in blue denotes values of scores and dashed lines in red mark alarms. **c,** the window sized for the sequential D-DML algorithm with adaptive window where lines in red mark the shrinkage of windows. **d,** the change scores produced by the 1st D-MDL. **e,** the change scores produced by the 2nd D-MDL.

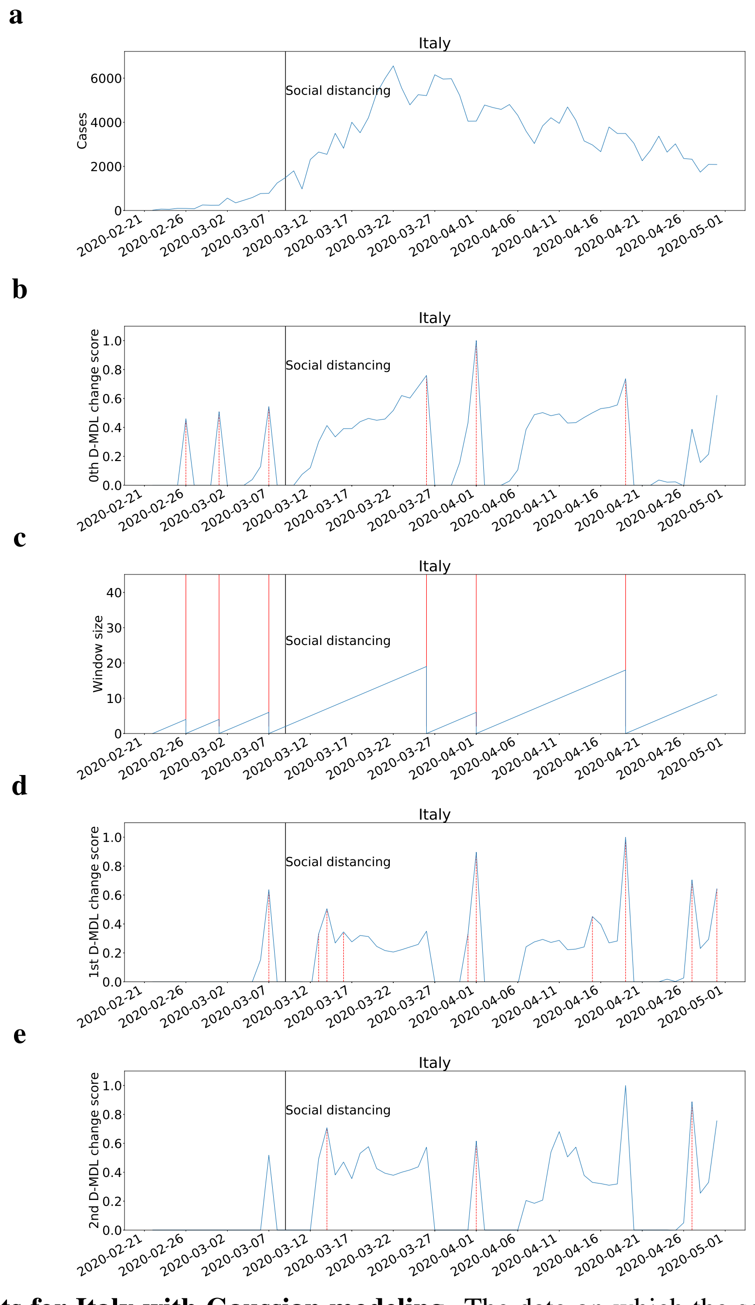

Fig. 33: The results for Italy with Gaussian modeling. The date on which the social distancing was implemented is marked by a solid line in black. **a,** the number of daily new cases. **b,** the change scores produced by the 0th M-DML where the line in blue denotes values of scores and dashed lines in red mark alarms. **c,** the window sized for the sequential D-DML algorithm with adaptive window where lines in red mark the shrinkage of windows. **d,** the change scores produced by the 1st D-MDL. **e,** the change scores produced by the 2nd D-MDL.

a

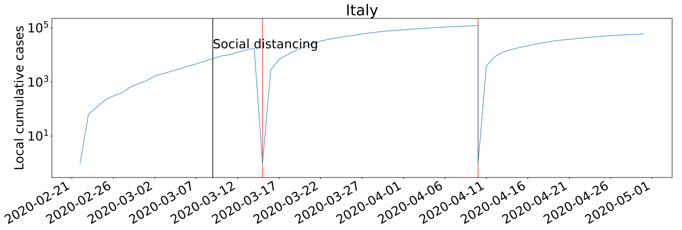

b

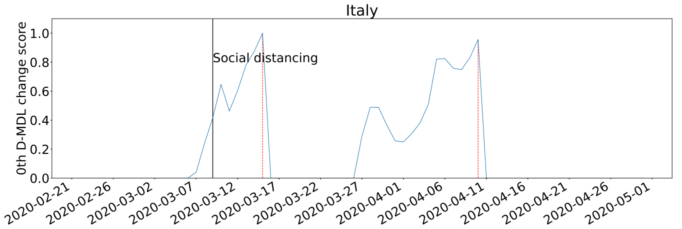

c

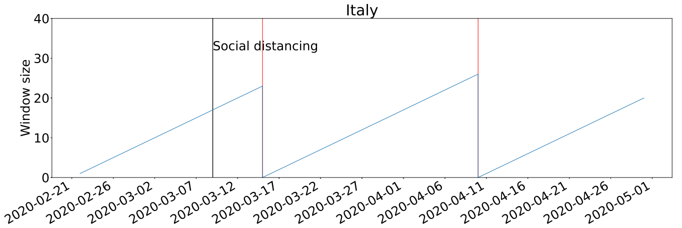

d

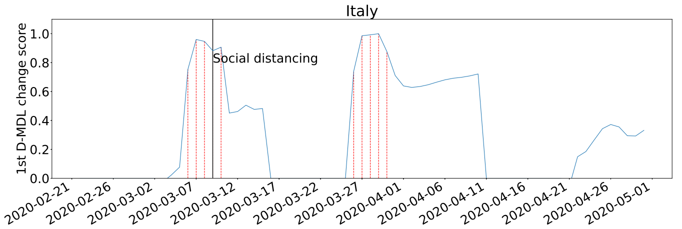

e

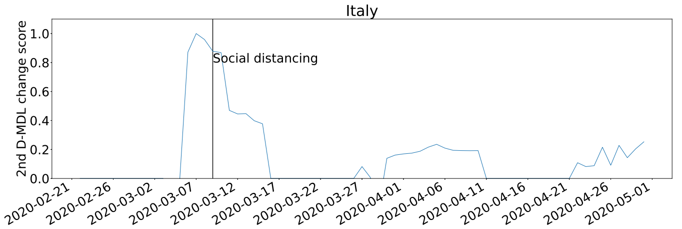

Fig. 34: The results for Italy with exponential modeling. The date on which the social distancing was implemented is marked by a solid line in black. **a,** the number of cumulative cases. **b,** the change scores produced by the 0th M-DML where the line in blue denotes values of scores and dashed lines in red mark alarms. **c,** the window sized for the sequential D-DML algorithm with adaptive window where lines in red mark the shrinkage of windows. **d,** the change scores produced by the 1st D-MDL. **e,** the change scores produced by the 2nd D-MDL.

a

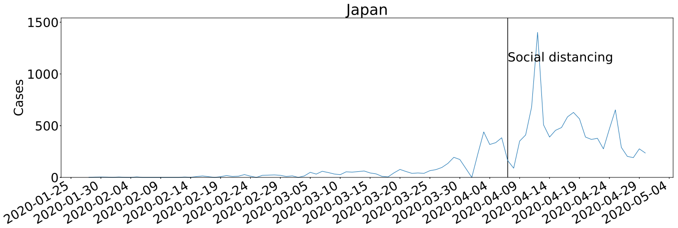

b

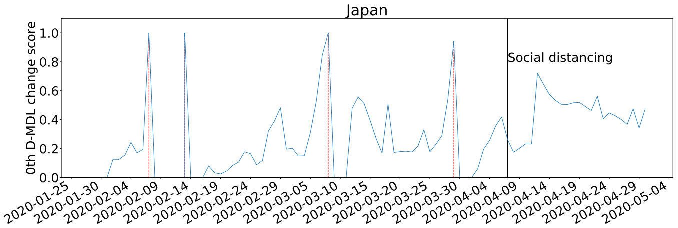

c

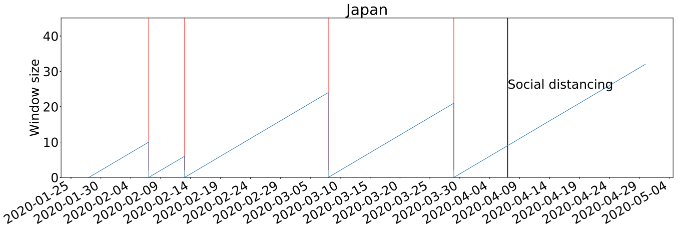

d

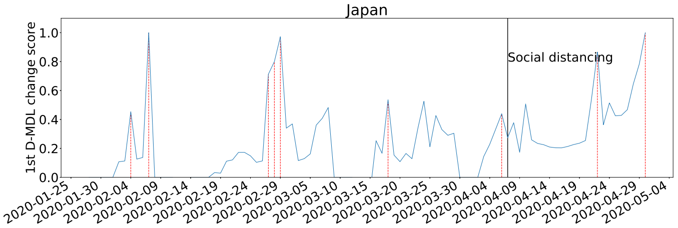

e

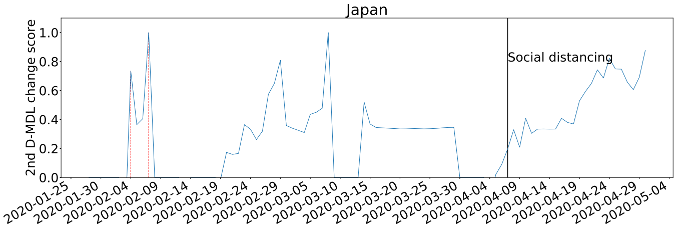

Fig. 35: The results for Japan with Gaussian modeling. The date on which the social distancing was implemented is marked by a solid line in black. **a,** the number of daily new cases. **b,** the change scores produced by the 0th M-DML where the line in blue denotes values of scores and dashed lines in red mark alarms. **c,** the window sized for the sequential D-DML algorithm with adaptive window where lines in red mark the shrinkage of windows. **d,** the change scores produced by the 1st D-MDL. **e,** the change scores produced by the 2nd D-MDL.

a

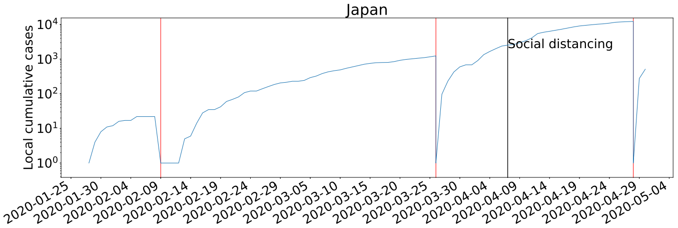

b

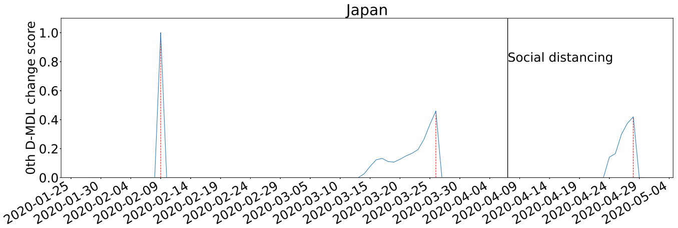

c

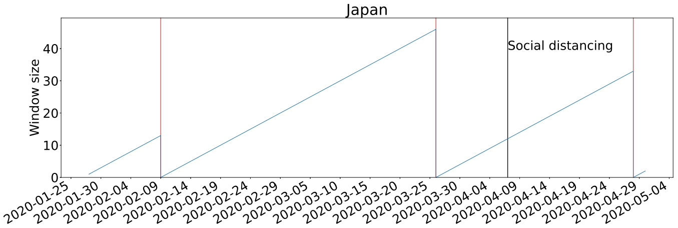

d

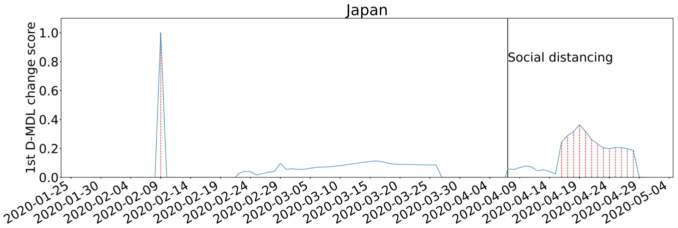

e

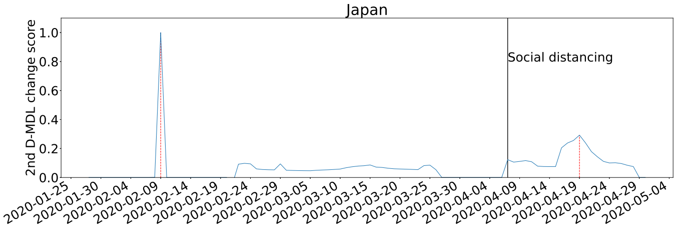

Fig. 36: The results for Japan with exponential modeling. The date on which the social distancing was implemented is marked by a solid line in black. **a,** the number of cumulative cases. **b,** the change scores produced by the 0th M-DML where the line in blue denotes values of scores and dashed lines in red mark alarms. **c,** the window sized for the sequential D-DML algorithm with adaptive window where lines in red mark the shrinkage of windows. **d,** the change scores produced by the 1st D-MDL. **e,** the change scores produced by the 2nd D-MDL.

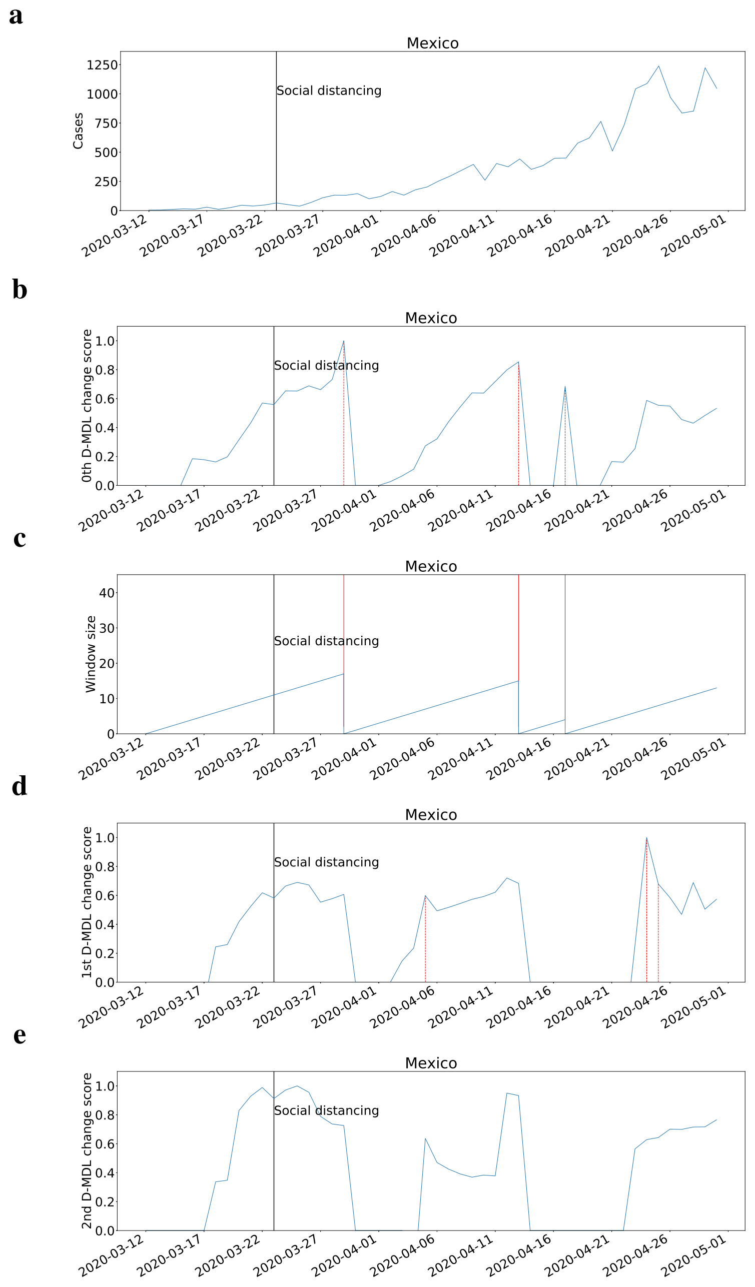

Fig. 37: The results for Mexico with Gaussian modeling. The date on which the social distancing was implemented is marked by a solid line in black. **a,** the number of daily new cases. **b,** the change scores produced by the 0th M-DML where the line in blue denotes values of scores and dashed lines in red mark alarms. **c,** the window sized for the sequential D-DML algorithm with adaptive window where lines in red mark the shrinkage of windows. **d,** the change scores produced by the 1st D-MDL. **e,** the change scores produced by the 2nd D-MDL.

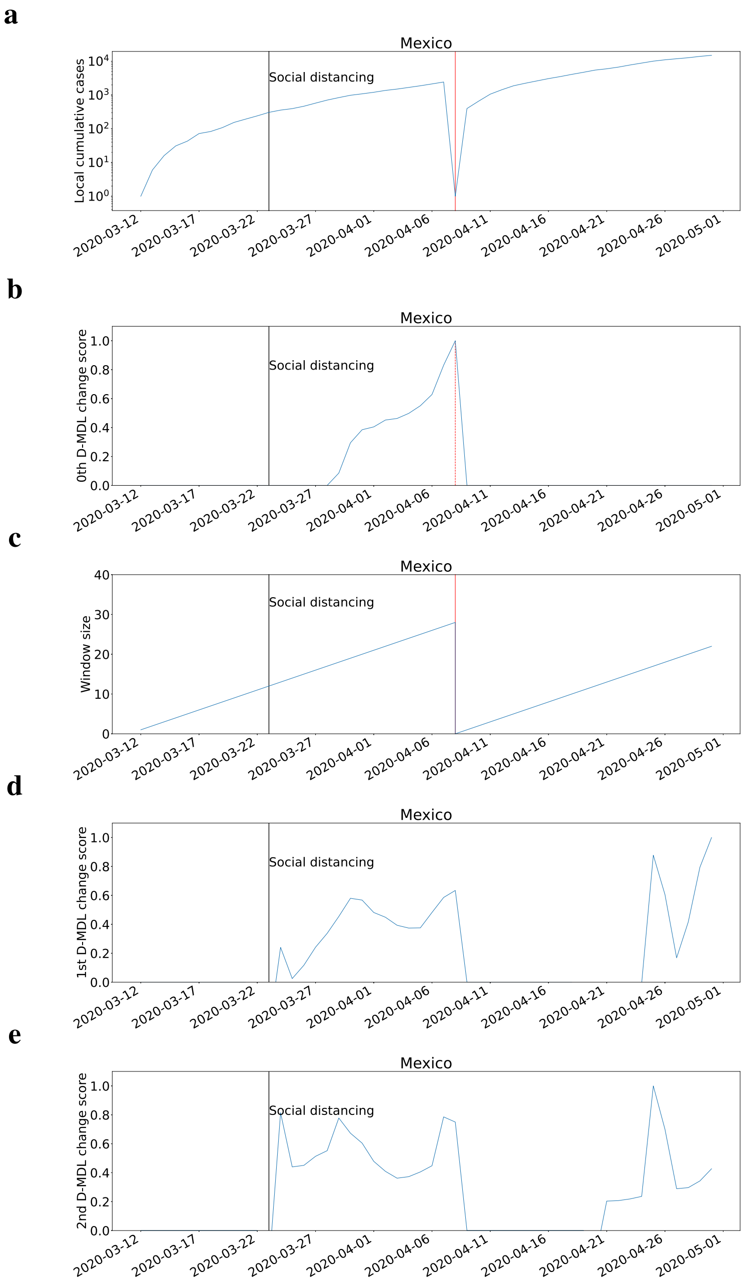

Fig. 38: The results for Mexico with exponential modeling. The date on which the social distancing was implemented is marked by a solid line in black. **a,** the number of cumulative cases. **b,** the change scores produced by the 0th M-DML where the line in blue denotes values of scores and dashed lines in red mark alarms. **c,** the window sized for the sequential D-DML algorithm with adaptive window where lines in red mark the shrinkage of windows. **d,** the change scores produced by the 1st D-MDL. **e,** the change scores produced by the 2nd D-MDL.

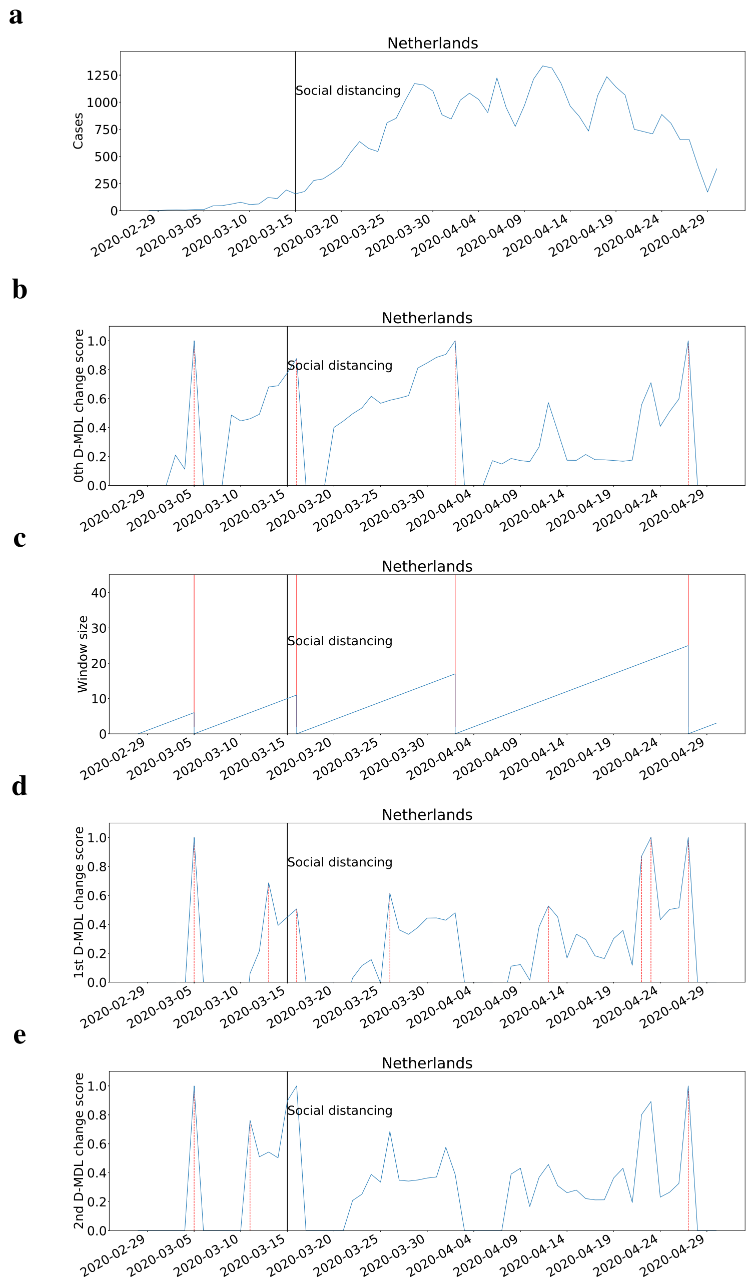

Fig. 39: The results for Netherlands with Gaussian modeling. The date on which the social distancing was implemented is marked by a solid line in black. **a,** the number of daily new cases. **b,** the change scores produced by the 0th M-DML where the line in blue denotes values of scores and dashed lines in red mark alarms. **c,** the window sized for the sequential D-DML algorithm with adaptive window where lines in red mark the shrinkage of windows. **d,** the change scores produced by the 1st D-MDL. **e,** the change scores produced by the 2nd D-MDL.

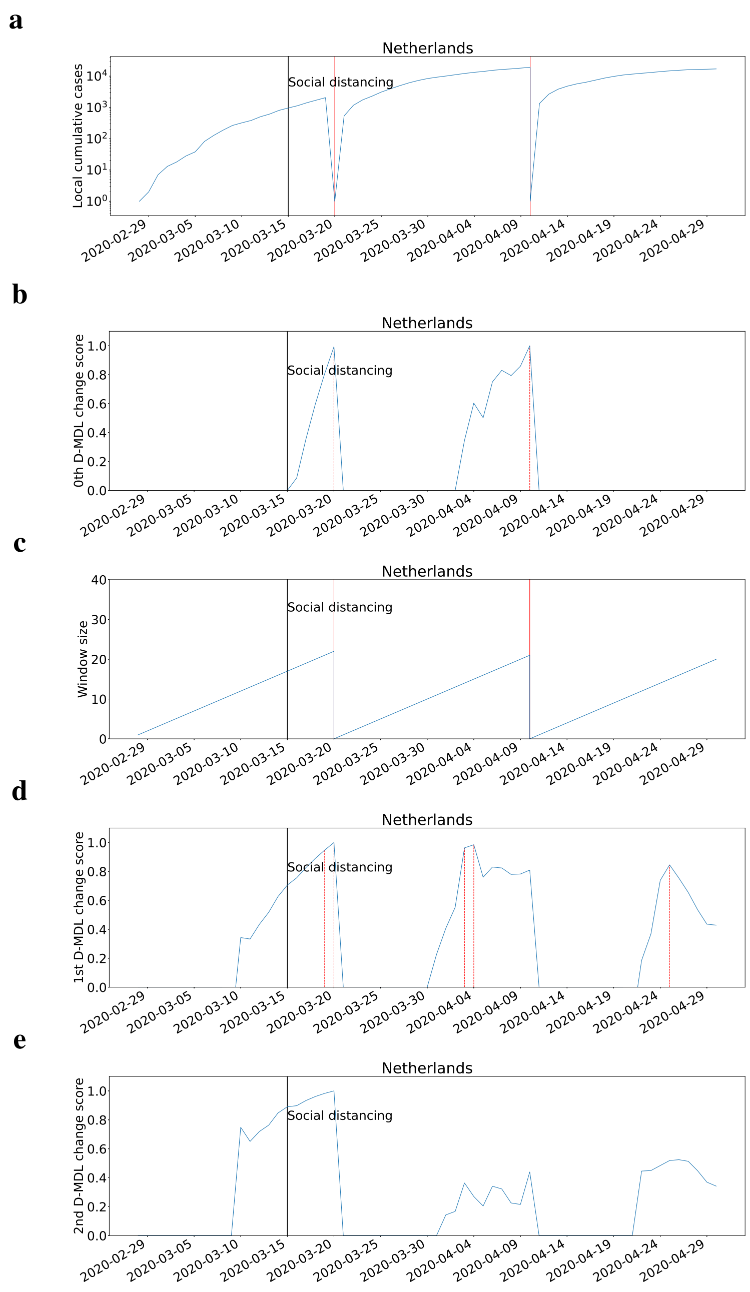

Fig. 40: The results for Netherlands with exponential modeling. The date on which the social distancing was implemented is marked by a solid line in black. **a,** the number of cumulative cases. **b,** the change scores produced by the 0th M-DML where the line in blue denotes values of scores and dashed lines in red mark alarms. **c,** the window sized for the sequential D-DML algorithm with adaptive window where lines in red mark the shrinkage of windows. **d,** the change scores produced by the 1st D-MDL. **e,** the change scores produced by the 2nd D-MDL.

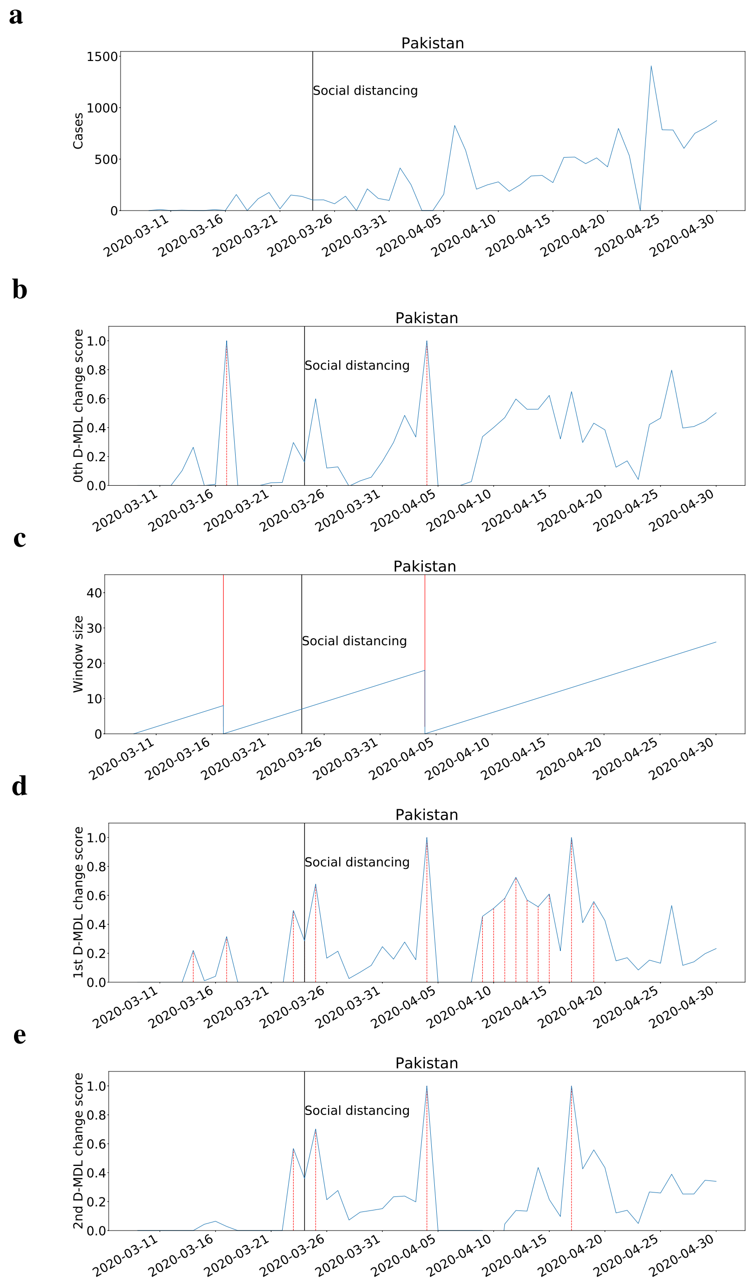

Fig. 41: The results for Pakistan with Gaussian modeling. The date on which the social distancing was implemented is marked by a solid line in black. **a,** the number of daily new cases. **b,** the change scores produced by the 0th M-DML where the line in blue denotes values of scores and dashed lines in red mark alarms. **c,** the window sized for the sequential D-DML algorithm with adaptive window where lines in red mark the shrinkage of windows. **d,** the change scores produced by the 1st D-MDL. **e,** the change scores produced by the 2nd D-MDL.

a

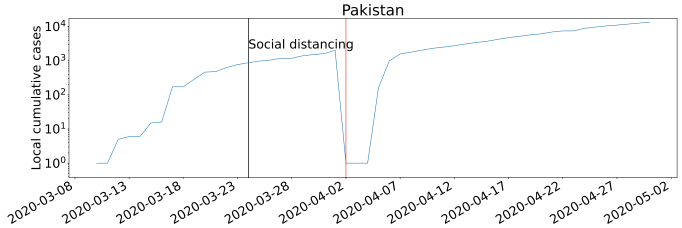

b

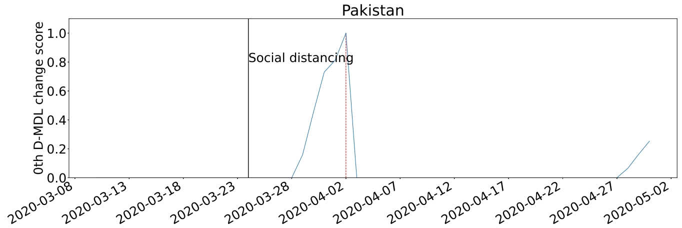

c

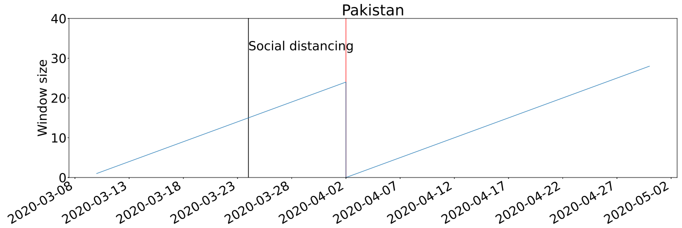

d

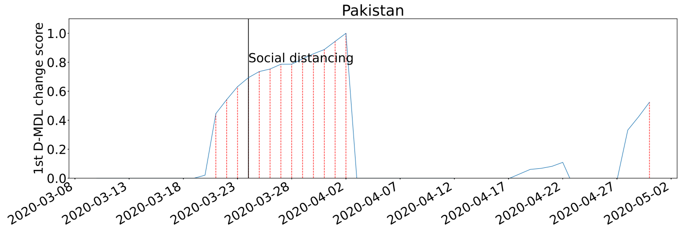

e

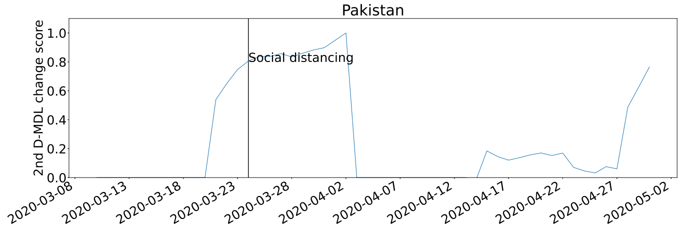

Fig. 42: The results for Pakistan with exponential modeling. The date on which the social distancing was implemented is marked by a solid line in black. **a,** the number of cumulative cases. **b,** the change scores produced by the 0th M-DML where the line in blue denotes values of scores and dashed lines in red mark alarms. **c,** the window sized for the sequential D-DML algorithm with adaptive window where lines in red mark the shrinkage of windows. **d,** the change scores produced by the 1st D-MDL. **e,** the change scores produced by the 2nd D-MDL.

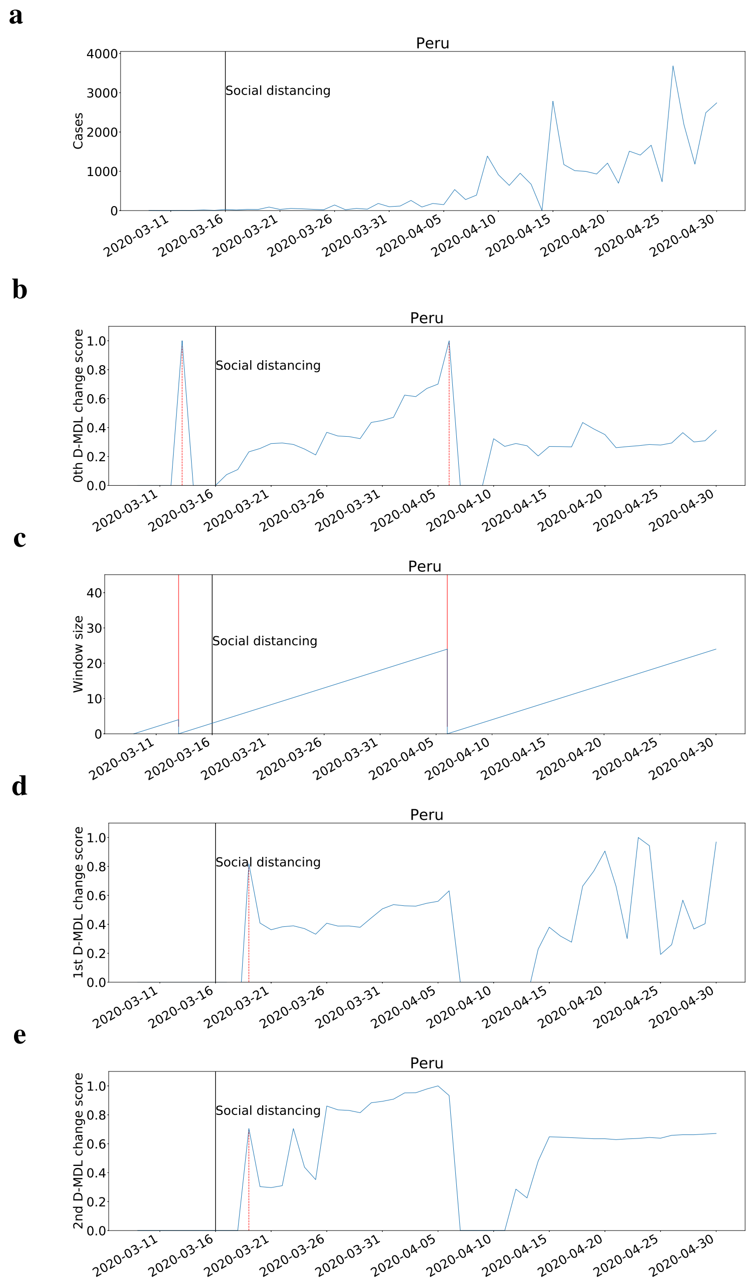

Fig. 43: The results for Peru with Gaussian modeling. The date on which the social distancing was implemented is marked by a solid line in black. **a,** the number of daily new cases. **b,** the change scores produced by the 0th M-DML where the line in blue denotes values of scores and dashed lines in red mark alarms. **c,** the window sized for the sequential D-DML algorithm with adaptive window where lines in red mark the shrinkage of windows. **d,** the change scores produced by the 1st D-MDL. **e,** the change scores produced by the 2nd D-MDL.

a

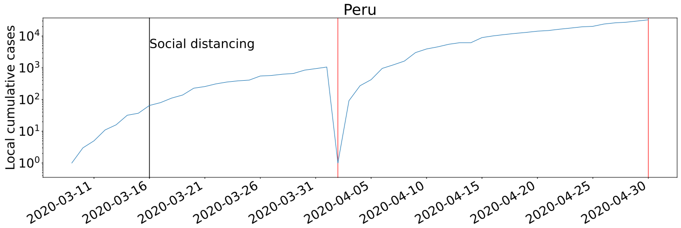

b

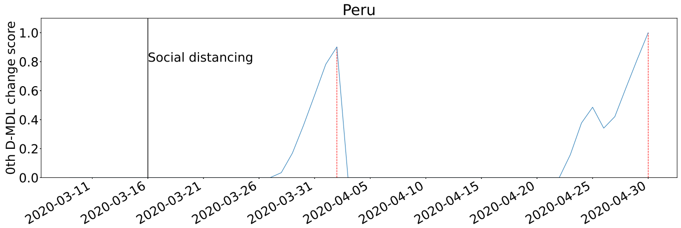

c

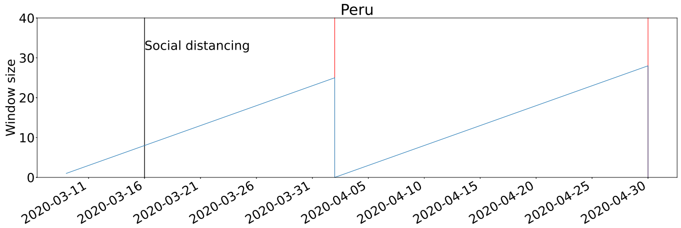

d

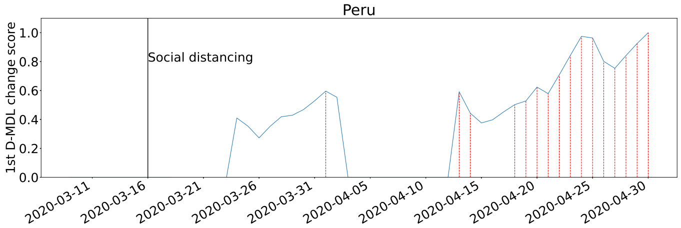

e

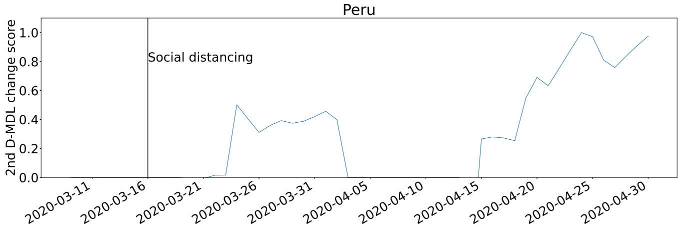

Fig. 44: The results for Peru with exponential modeling. The date on which the social distancing was implemented is marked by a solid line in black. **a,** the number of cumulative cases. **b,** the change scores produced by the 0th M-DML where the line in blue denotes values of scores and dashed lines in red mark alarms. **c,** the window sized for the sequential D-DML algorithm with adaptive window where lines in red mark the shrinkage of windows. **d,** the change scores produced by the 1st D-MDL. **e,** the change scores produced by the 2nd D-MDL.

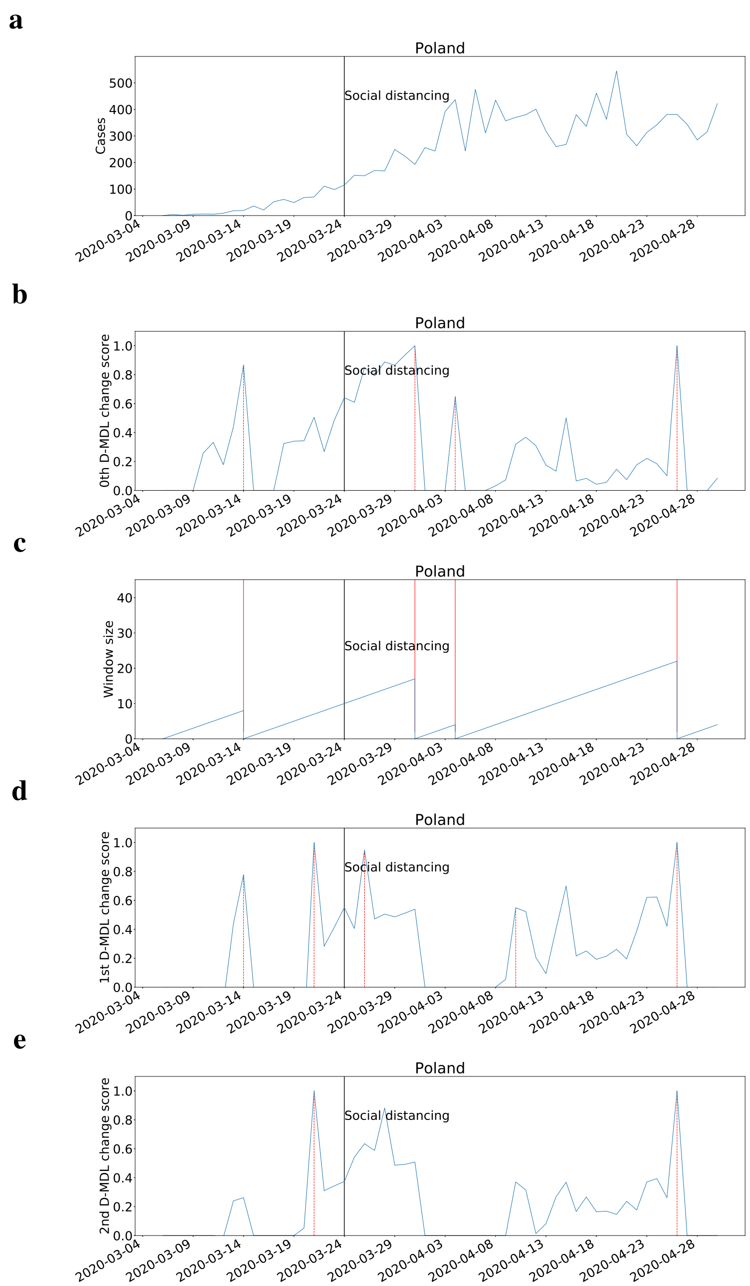

Fig. 45: The results for Poland with Gaussian modeling. The date on which the social distancing was implemented is marked by a solid line in black. **a,** the number of daily new cases. **b,** the change scores produced by the 0th M-DML where the line in blue denotes values of scores and dashed lines in red mark alarms. **c,** the window sized for the sequential D-DML algorithm with adaptive window where lines in red mark the shrinkage of windows. **d,** the change scores produced by the 1st D-MDL. **e,** the change scores produced by the 2nd D-MDL.

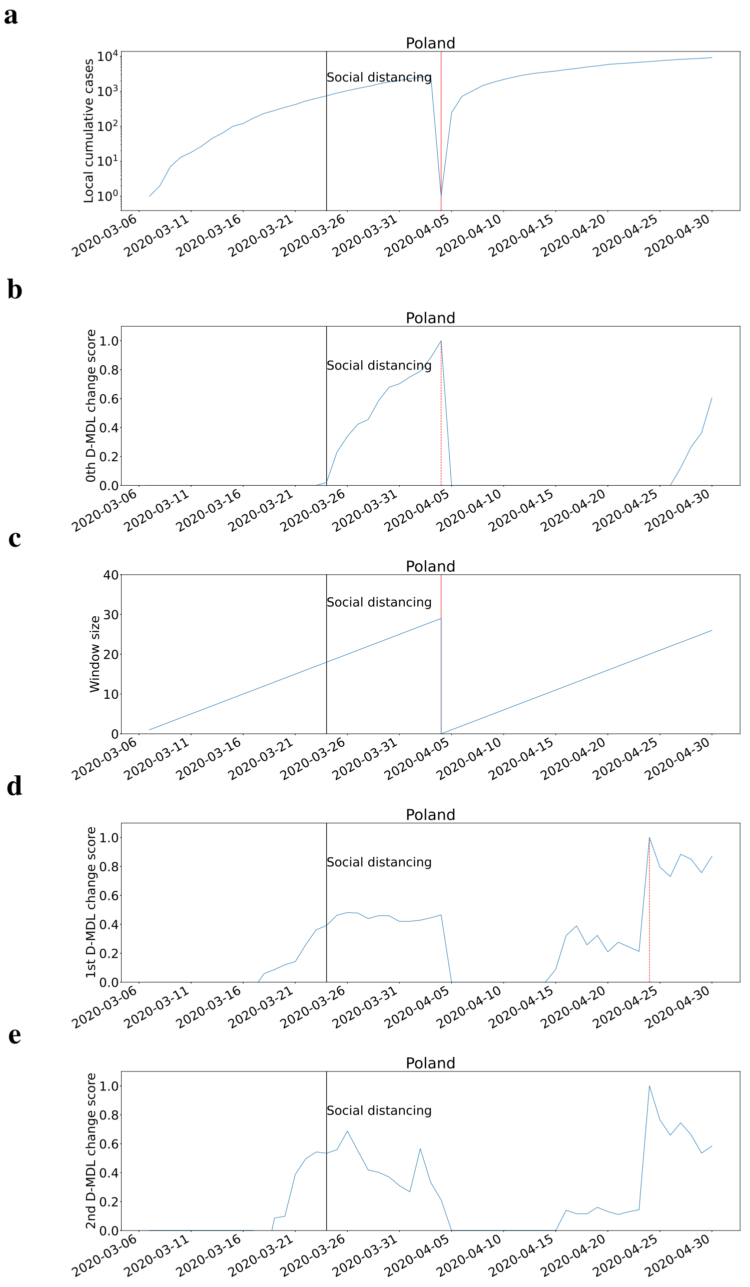

Fig. 46: The results for Poland with exponential modeling. The date on which the social distancing was implemented is marked by a solid line in black. **a,** the number of cumulative cases. **b,** the change scores produced by the 0th M-DML where the line in blue denotes values of scores and dashed lines in red mark alarms. **c,** the window sized for the sequential D-DML algorithm with adaptive window where lines in red mark the shrinkage of windows. **d,** the change scores produced by the 1st D-MDL. **e,** the change scores produced by the 2nd D-MDL.

a

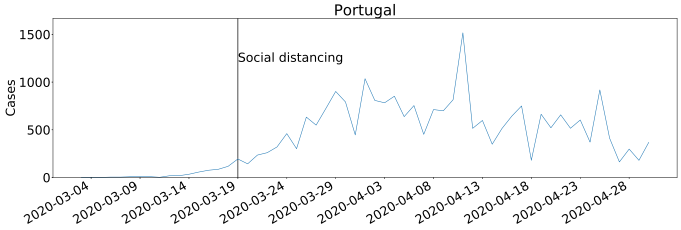

b

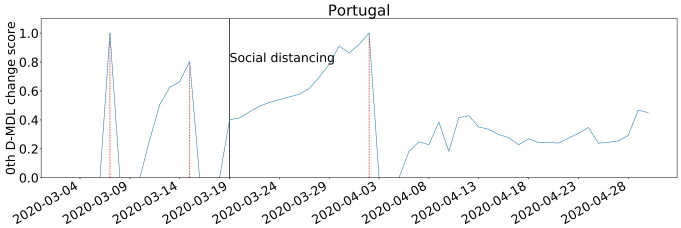

c

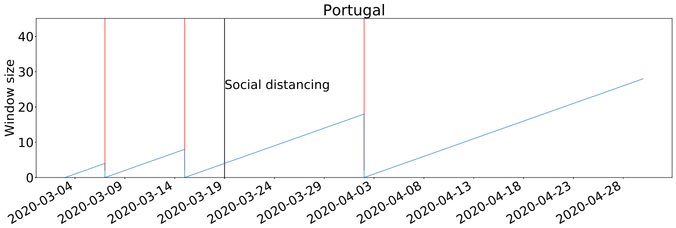

d

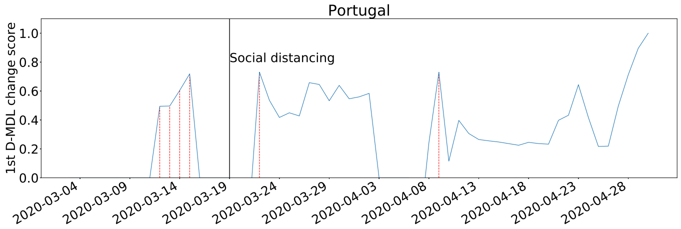

e

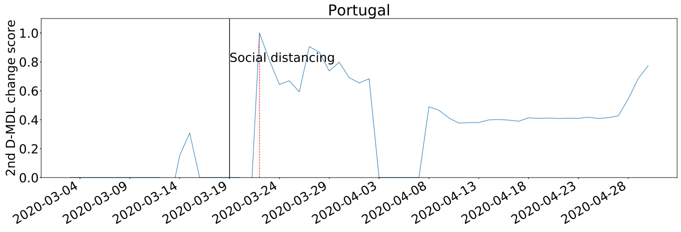

Fig. 47: The results for Portugal with Gaussian modeling. The date on which the social distancing was implemented is marked by a solid line in black. **a,** the number of daily new cases. **b,** the change scores produced by the 0th M-DML where the line in blue denotes values of scores and dashed lines in red mark alarms. **c,** the window sized for the sequential D-DML algorithm with adaptive window where lines in red mark the shrinkage of windows. **d,** the change scores produced by the 1st D-MDL. **e,** the change scores produced by the 2nd D-MDL.

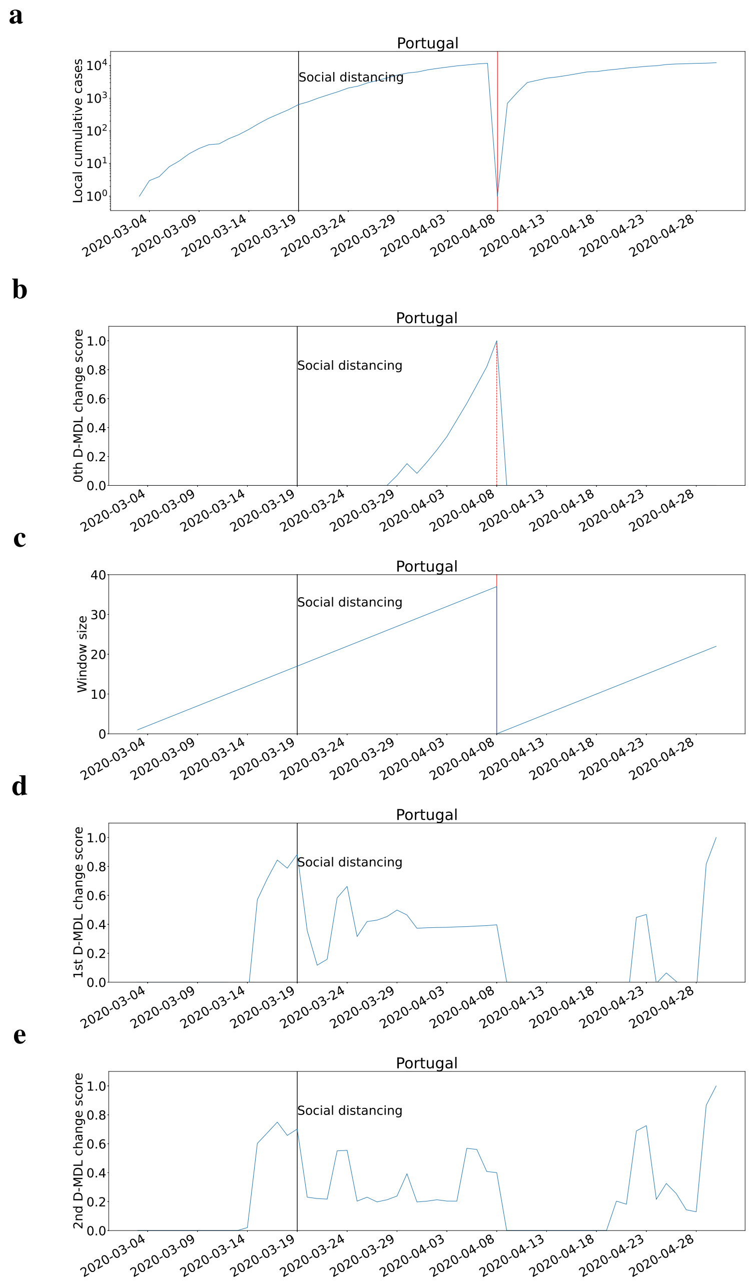

Fig. 48: The results for Portugal with exponential modeling. The date on which the social distancing was implemented is marked by a solid line in black. **a,** the number of cumulative cases. **b,** the change scores produced by the 0th M-DML where the line in blue denotes values of scores and dashed lines in red mark alarms. **c,** the window sized for the sequential D-DML algorithm with adaptive window where lines in red mark the shrinkage of windows. **d,** the change scores produced by the 1st D-MDL. **e,** the change scores produced by the 2nd D-MDL.

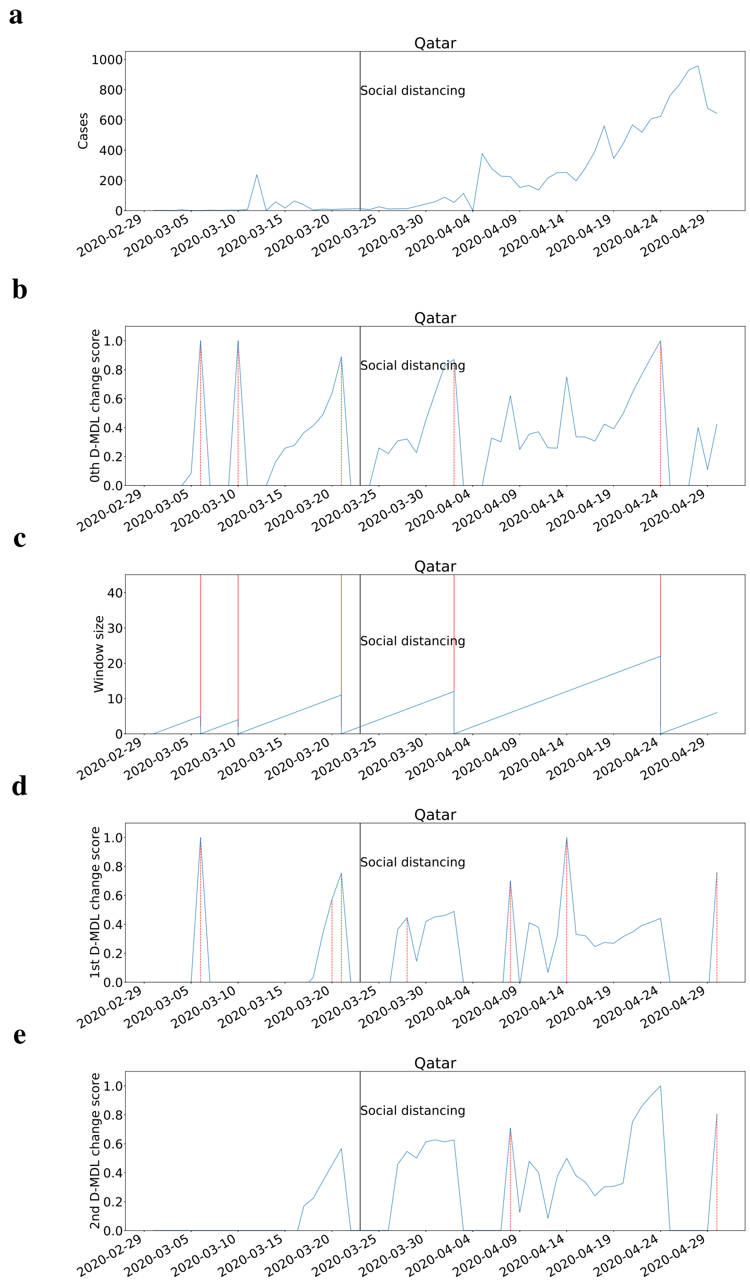

Fig. 49: The results for Qatar with Gaussian modeling. The date on which the social distancing was implemented is marked by a solid line in black. **a,** the number of daily new cases. **b,** the change scores produced by the 0th M-DML where the line in blue denotes values of scores and dashed lines in red mark alarms. **c,** the window sized for the sequential D-DML algorithm with adaptive window where lines in red mark the shrinkage of windows. **d,** the change scores produced by the 1st D-MDL. **e,** the change scores produced by the 2nd D-MDL.

a

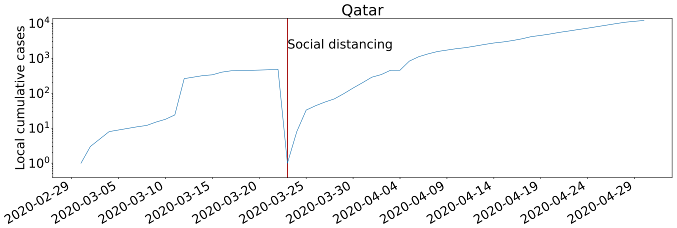

b

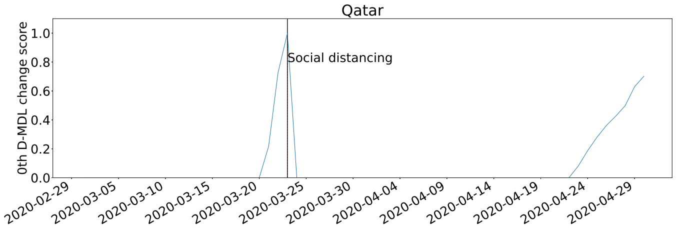

c

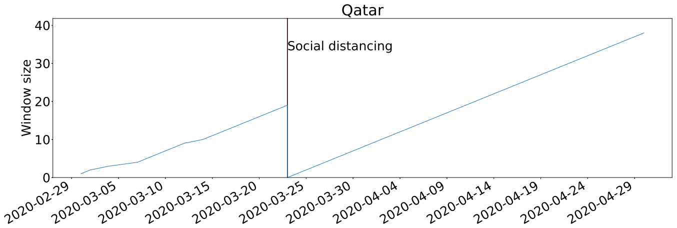

d

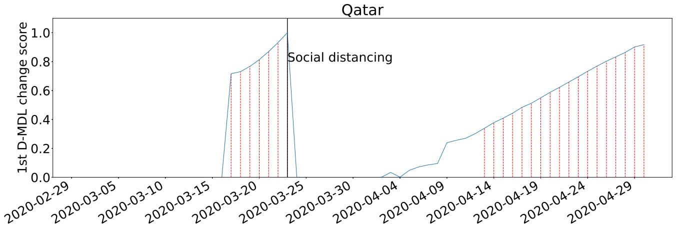

e

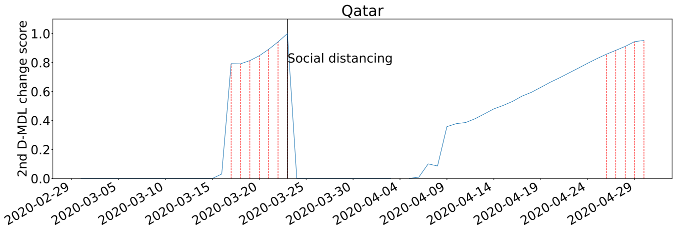

Fig. 50: The results for Qatar with exponential modeling. The date on which the social distancing was implemented is marked by a solid line in black. **a,** the number of cumulative cases. **b,** the change scores produced by the 0th M-DML where the line in blue denotes values of scores and dashed lines in red mark alarms. **c,** the window sized for the sequential D-DML algorithm with adaptive window where lines in red mark the shrinkage of windows. **d,** the change scores produced by the 1st D-MDL. **e,** the change scores produced by the 2nd D-MDL.

a

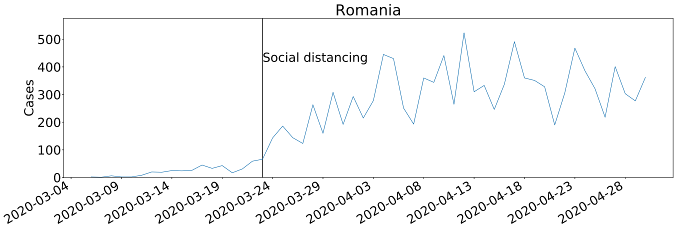

b

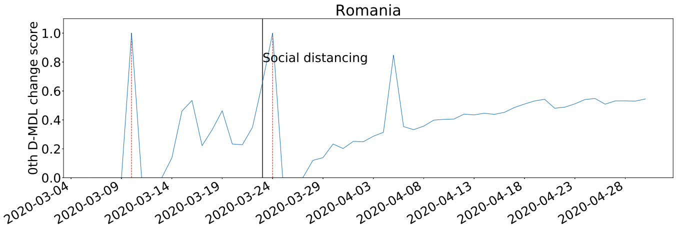

c

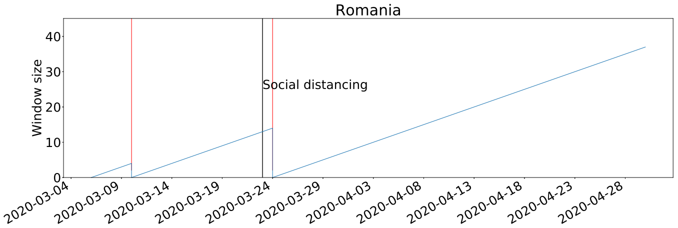

d

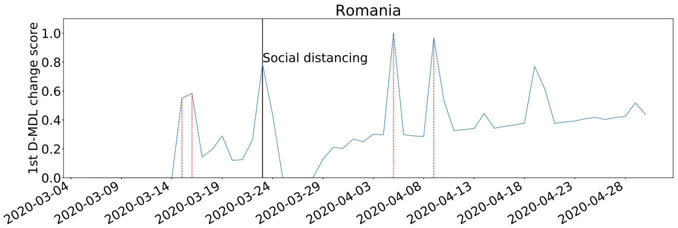

e

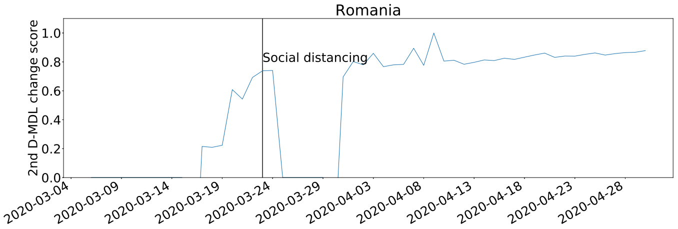

Fig. 51: The results for Romania with Gaussian modeling. The date on which the social distancing was implemented is marked by a solid line in black. **a,** the number of daily new cases. **b,** the change scores produced by the 0th M-DML where the line in blue denotes values of scores and dashed lines in red mark alarms. **c,** the window sized for the sequential D-DML algorithm with adaptive window where lines in red mark the shrinkage of windows. **d,** the change scores produced by the 1st D-MDL. **e,** the change scores produced by the 2nd D-MDL.

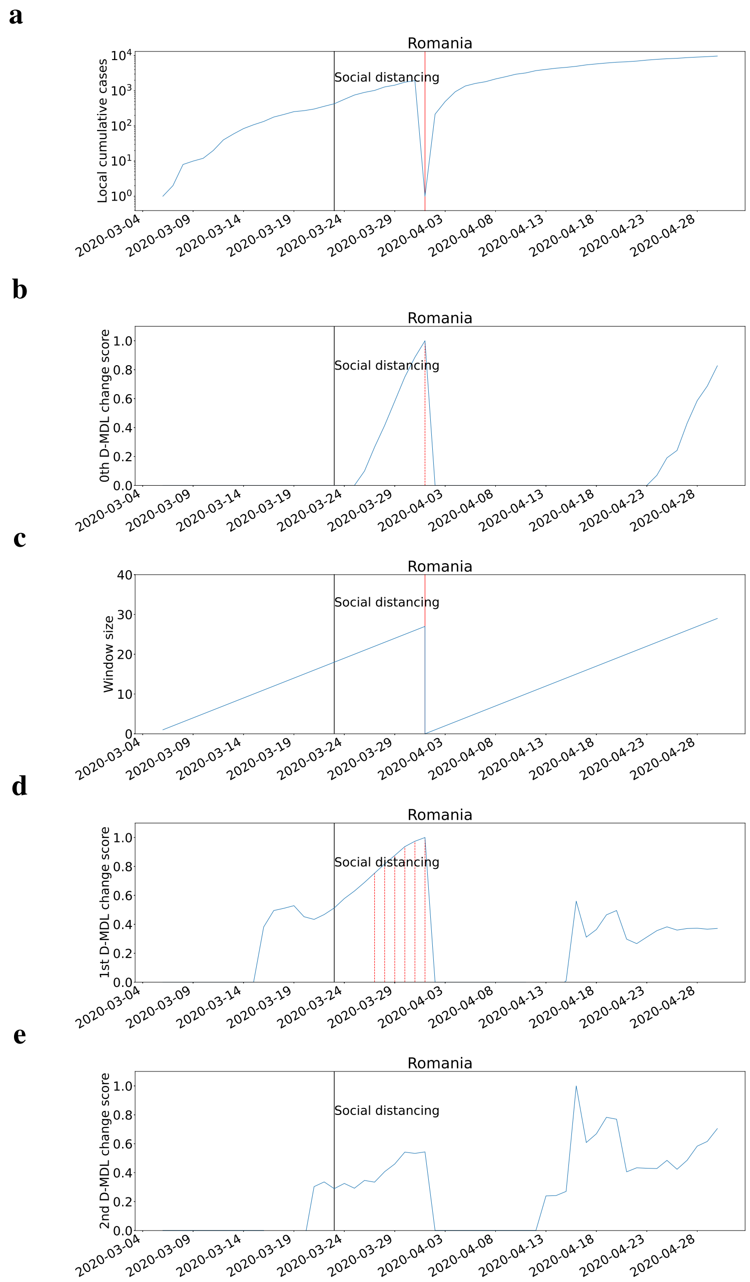

Fig. 52: The results for Romania with exponential modeling. The date on which the social distancing was implemented is marked by a solid line in black. **a,** the number of cumulative cases. **b,** the change scores produced by the 0th M-DML where the line in blue denotes values of scores and dashed lines in red mark alarms. **c,** the window sized for the sequential D-DML algorithm with adaptive window where lines in red mark the shrinkage of windows. **d,** the change scores produced by the 1st D-MDL. **e,** the change scores produced by the 2nd D-MDL.

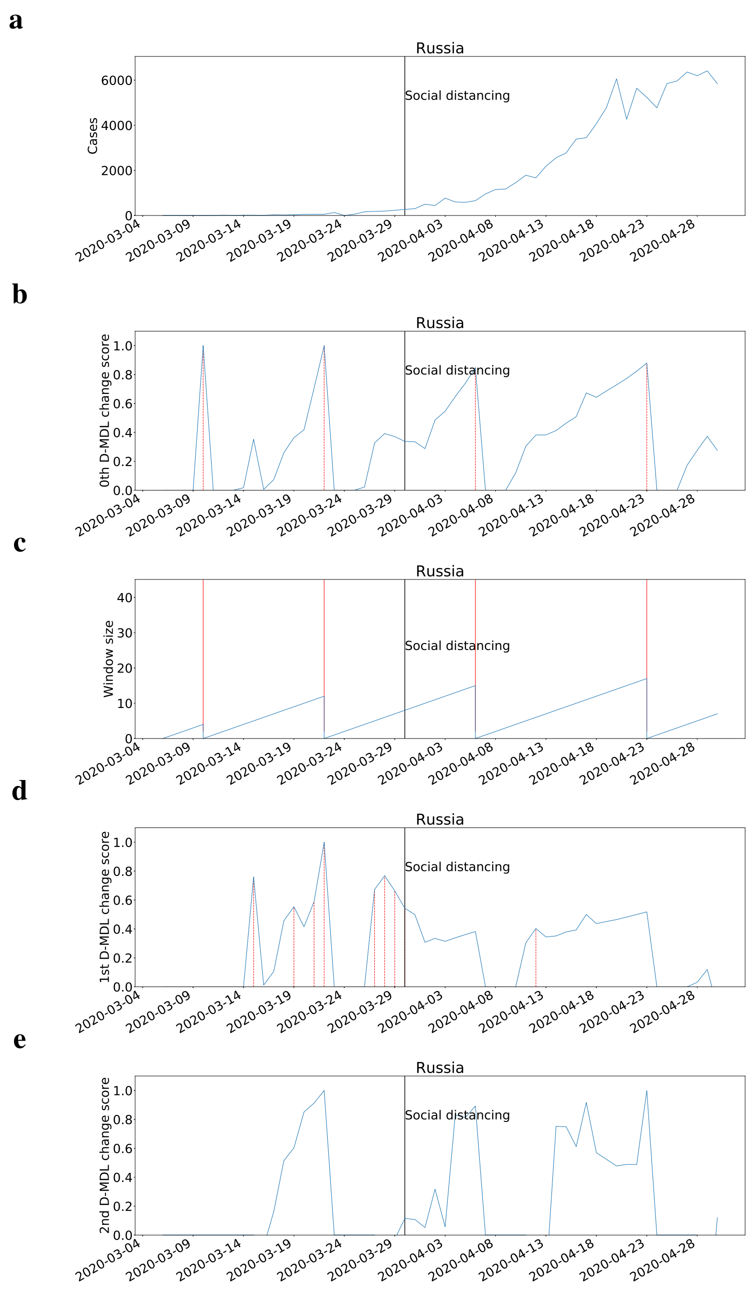

Fig. 53: The results for Russia with Gaussian modeling. The date on which the social distancing was implemented is marked by a solid line in black. **a,** the number of daily new cases. **b,** the change scores produced by the 0th M-DML where the line in blue denotes values of scores and dashed lines in red mark alarms. **c,** the window sized for the sequential D-DML algorithm with adaptive window where lines in red mark the shrinkage of windows. **d,** the change scores produced by the 1st D-MDL. **e,** the change scores produced by the 2nd D-MDL.

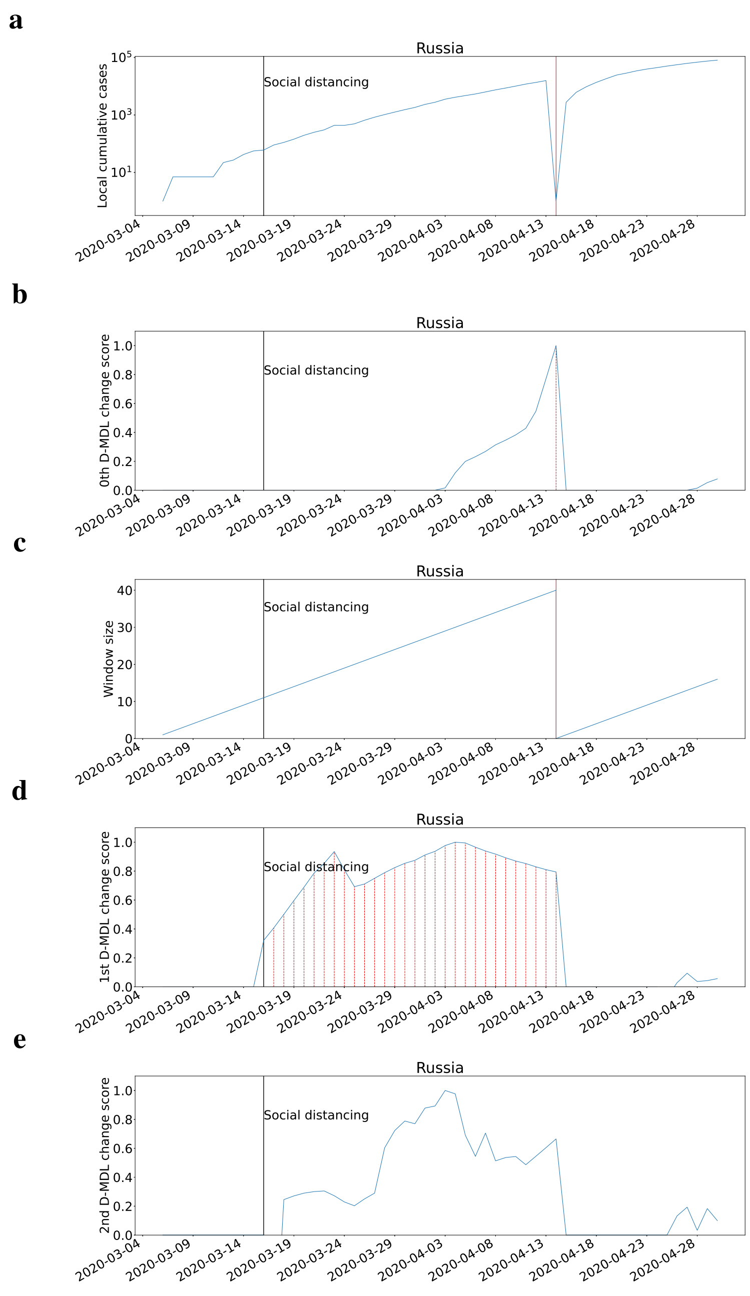

Fig. 54: The results for Russia with exponential modeling. The date on which the social distancing was implemented is marked by a solid line in black. **a,** the number of cumulative cases. **b,** the change scores produced by the 0th M-DML where the line in blue denotes values of scores and dashed lines in red mark alarms. **c,** the window sized for the sequential D-DML algorithm with adaptive window where lines in red mark the shrinkage of windows. **d,** the change scores produced by the 1st D-MDL. **e,** the change scores produced by the 2nd D-MDL.

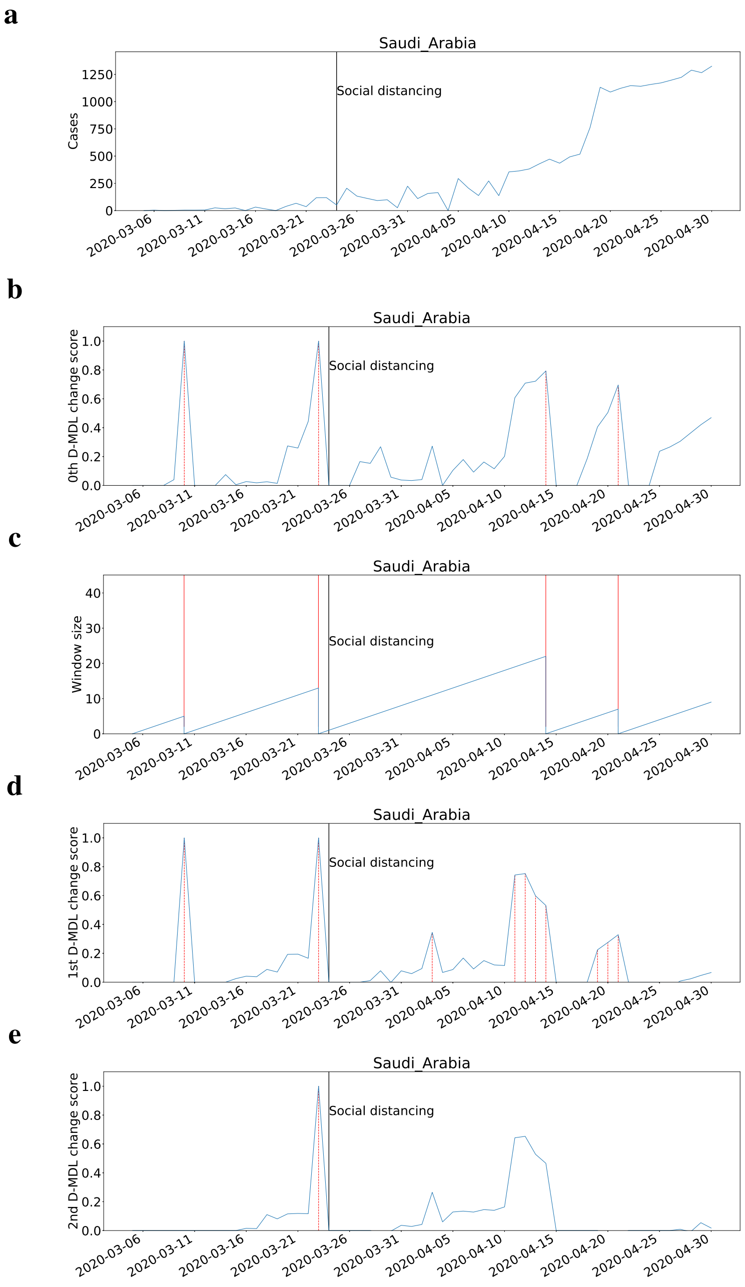

Fig. 55: The results for Saudi Arabia with Gaussian modeling. The date on which the social distancing was implemented is marked by a solid line in black. **a,** the number of daily new cases. **b,** the change scores produced by the 0th M-DML where the line in blue denotes values of scores and dashed lines in red mark alarms. **c,** the window sized for the sequential D-DML algorithm with adaptive window where lines in red mark the shrinkage of windows. **d,** the change scores produced by the 1st D-MDL. **e,** the change scores produced by the 2nd D-MDL.

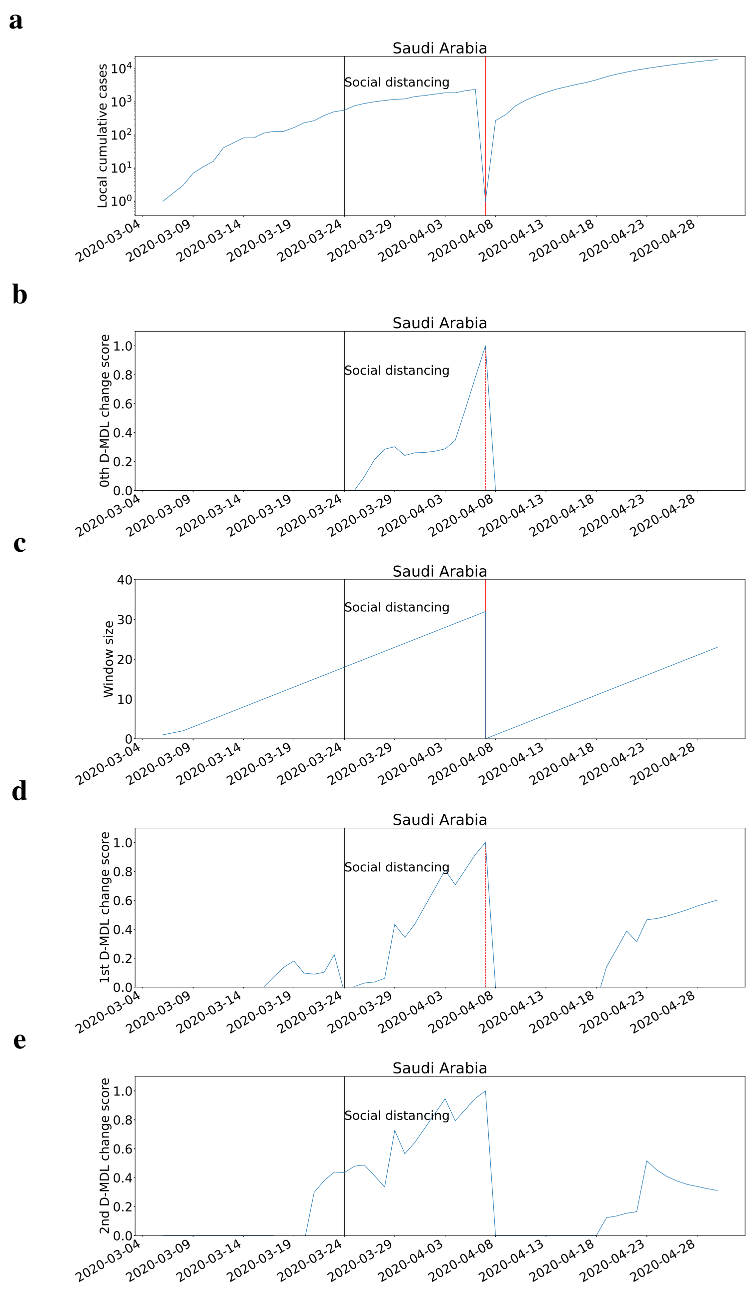

Fig. 56: The results for Saudi Arabia with exponential modeling. The date on which the social distancing was implemented is marked by a solid line in black. **a,** the number of cumulative cases. **b,** the change scores produced by the 0th M-DML where the line in blue denotes values of scores and dashed lines in red mark alarms. **c,** the window sized for the sequential D-DML algorithm with adaptive window where lines in red mark the shrinkage of windows. **d,** the change scores produced by the 1st D-MDL. **e,** the change scores produced by the 2nd D-MDL.

a

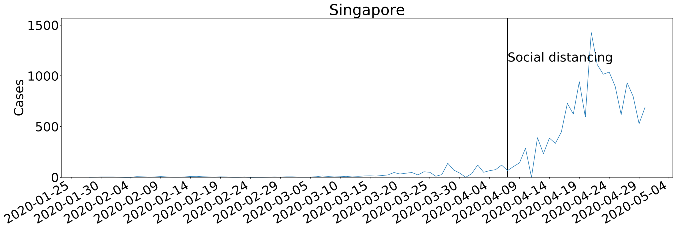

b

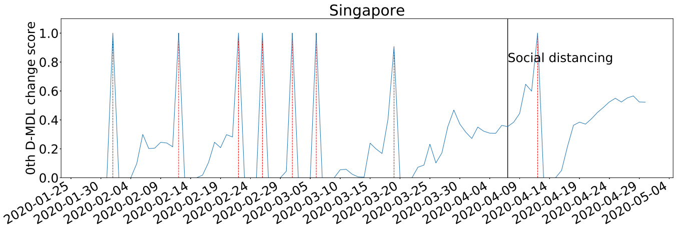

c

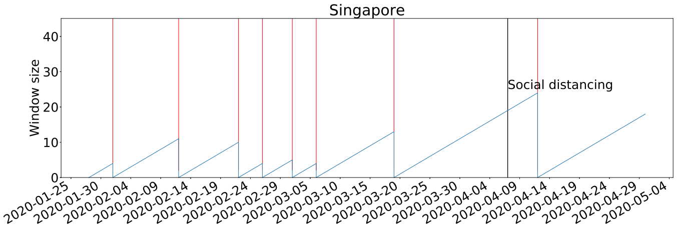

d

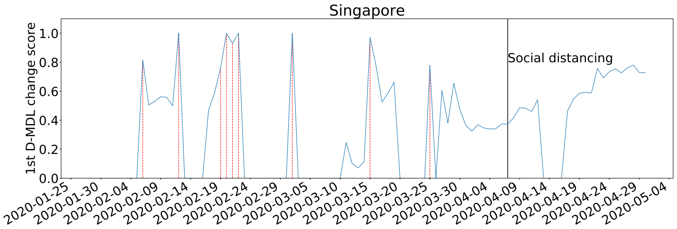

e

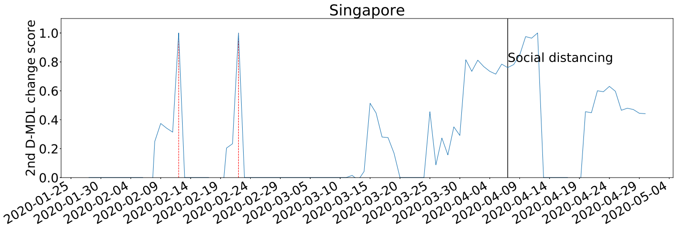

Fig. 57: The results for Singapore with Gaussian modeling. The date on which the social distancing was implemented is marked by a solid line in black. **a,** the number of daily new cases. **b,** the change scores produced by the 0th M-DML where the line in blue denotes values of scores and dashed lines in red mark alarms. **c,** the window sized for the sequential D-DML algorithm with adaptive window where lines in red mark the shrinkage of windows. **d,** the change scores produced by the 1st D-MDL. **e,** the change scores produced by the 2nd D-MDL.

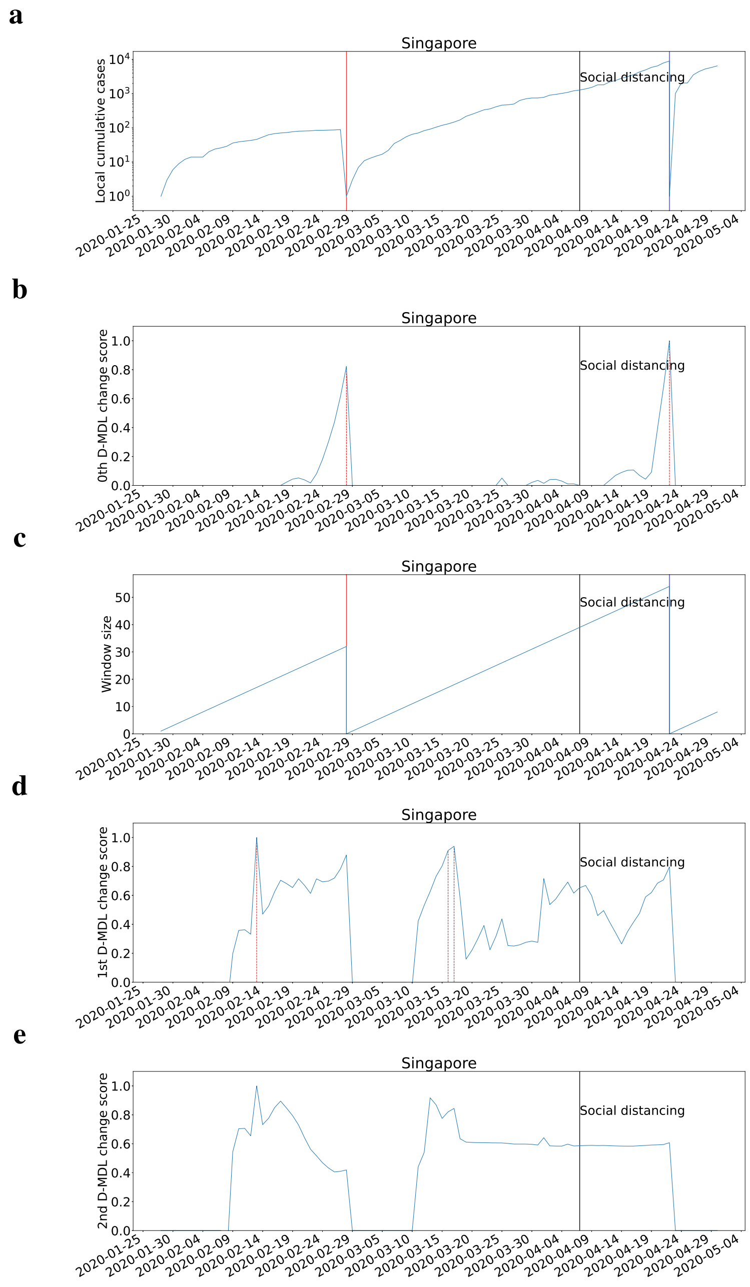

Fig. 58: The results for Singapore with exponential modeling. The date on which the social distancing was implemented is marked by a solid line in black. **a,** the number of cumulative cases. **b,** the change scores produced by the 0th M-DML where the line in blue denotes values of scores and dashed lines in red mark alarms. **c,** the window sized for the sequential D-DML algorithm with adaptive window where lines in red mark the shrinkage of windows. **d,** the change scores produced by the 1st D-MDL. **e,** the change scores produced by the 2nd D-MDL.

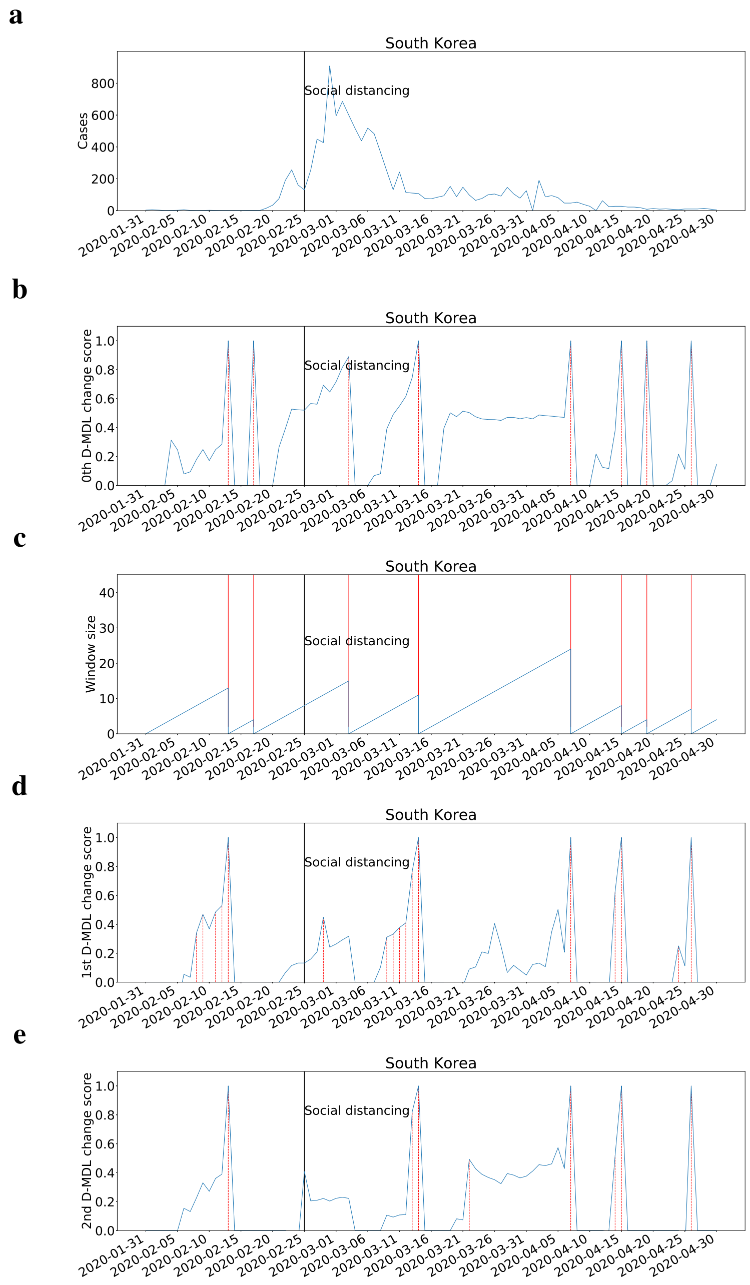

Fig. 59: The results for South Korea with Gaussian modeling. The date on which the social distancing was implemented is marked by a solid line in black. **a,** the number of daily new cases. **b,** the change scores produced by the 0th M-DML where the line in blue denotes values of scores and dashed lines in red mark alarms. **c,** the window sized for the sequential D-DML algorithm with adaptive window where lines in red mark the shrinkage of windows. **d,** the change scores produced by the 1st D-MDL. **e,** the change scores produced by the 2nd D-MDL.

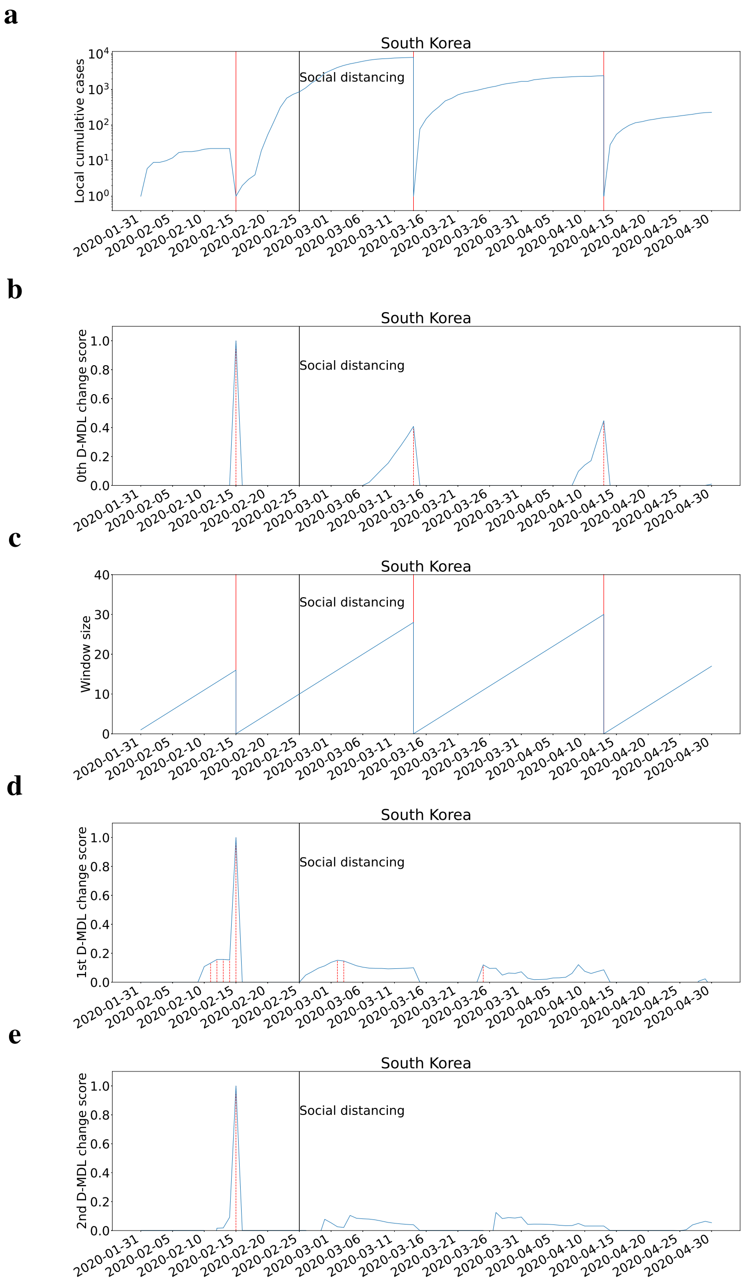

Fig. 60: The results for South Korea with exponential modeling. The date on which the social distancing was implemented is marked by a solid line in black. **a,** the number of cumulative cases. **b,** the change scores produced by the 0th M-DML where the line in blue denotes values of scores and dashed lines in red mark alarms. **c,** the window sized for the sequential D-DML algorithm with adaptive window where lines in red mark the shrinkage of windows. **d,** the change scores produced by the 1st D-MDL. **e,** the change scores produced by the 2nd D-MDL.

a

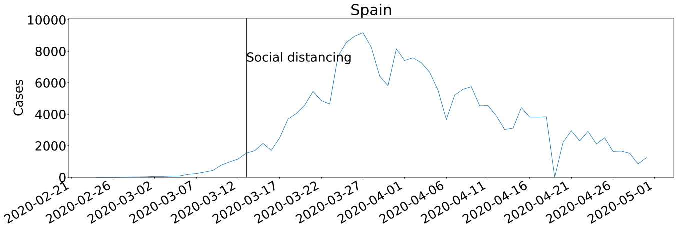

b

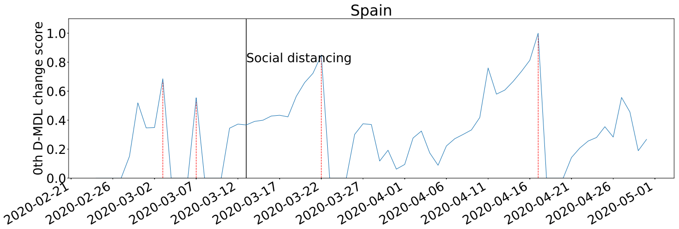

c

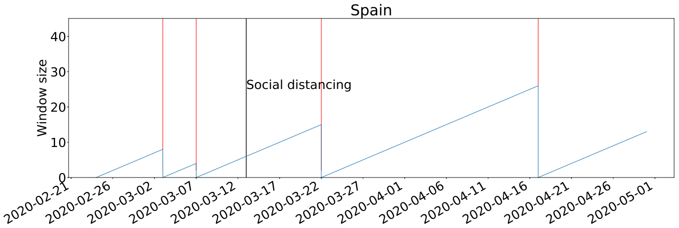

d

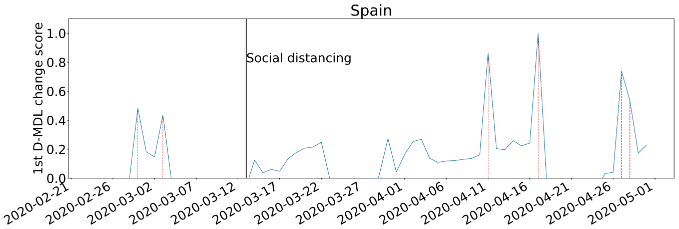

e

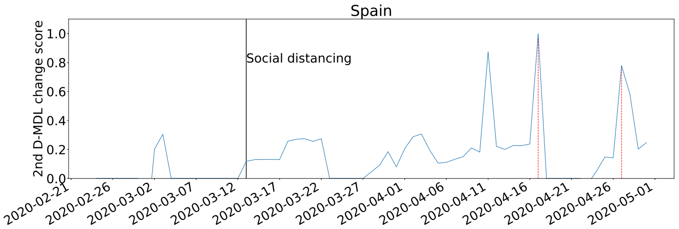

Fig. 61: The results for Spain with Gaussian modeling. The date on which the social distancing was implemented is marked by a solid line in black. **a,** the number of daily new cases. **b,** the change scores produced by the 0th M-DML where the line in blue denotes values of scores and dashed lines in red mark alarms. **c,** the window sized for the sequential D-DML algorithm with adaptive window where lines in red mark the shrinkage of windows. **d,** the change scores produced by the 1st D-MDL. **e,** the change scores produced by the 2nd D-MDL.

a

b

c

d

e

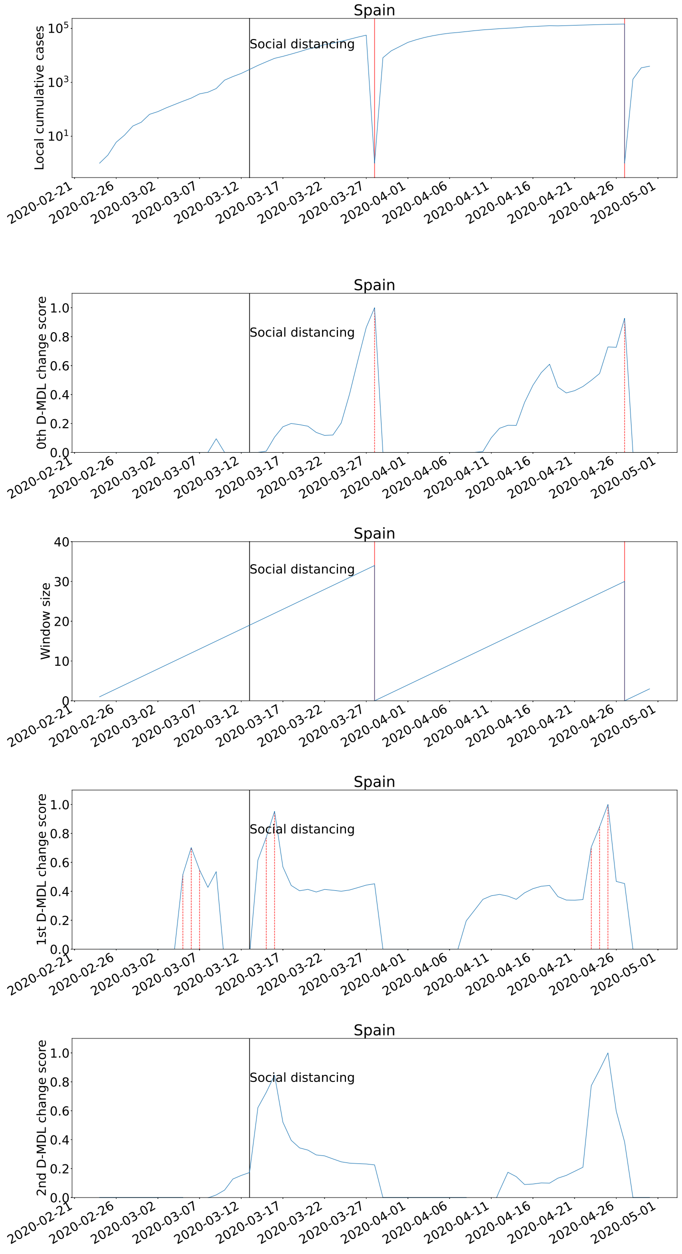

Fig. 62: The results for Spain with exponential modeling. The date on which the social distancing was implemented is marked by a solid line in black. **a,** the number of cumulative cases. **b,** the change scores produced by the 0th M-DML where the line in blue denotes values of scores and dashed lines in red mark alarms. **c,** the window sized for the sequential D-DML algorithm with adaptive window where lines in red mark the shrinkage of windows. **d,** the change scores produced by the 1st D-MDL. **e,** the change scores produced by the 2nd D-MDL.

a

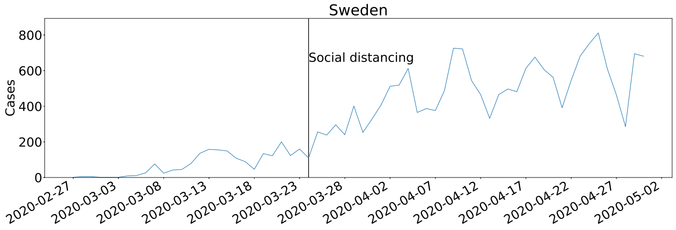

b

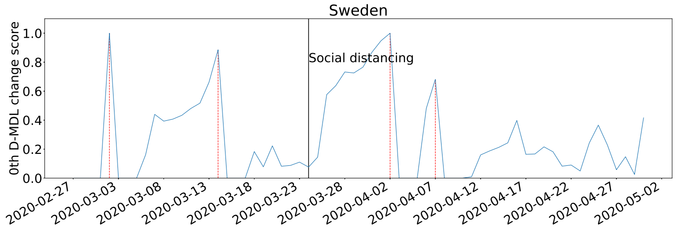

c

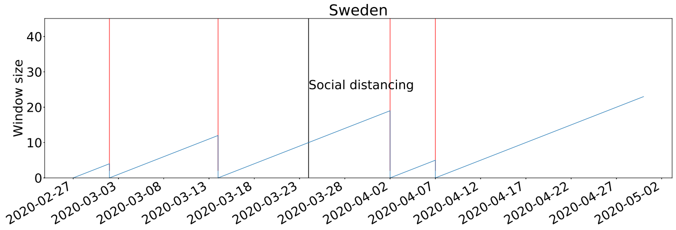

d

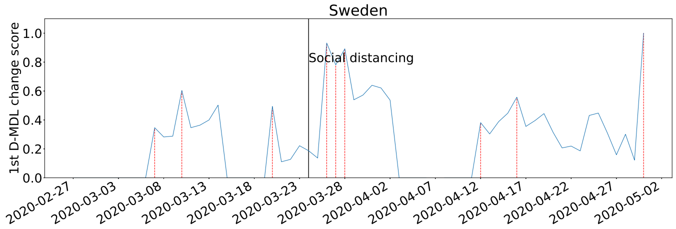

e

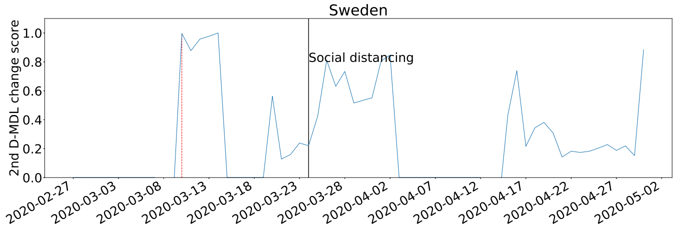

Fig. 63: The results for Sweden with Gaussian modeling. The date on which the social distancing was implemented is marked by a solid line in black. **a,** the number of daily new cases. **b,** the change scores produced by the 0th M-DML where the line in blue denotes values of scores and dashed lines in red mark alarms. **c,** the window sized for the sequential D-DML algorithm with adaptive window where lines in red mark the shrinkage of windows. **d,** the change scores produced by the 1st D-MDL. **e,** the change scores produced by the 2nd D-MDL.

a

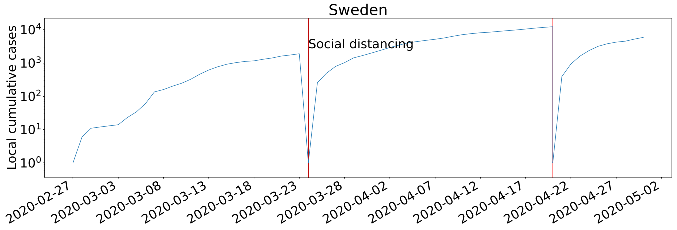

b

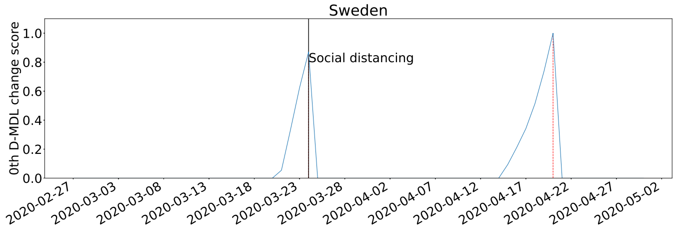

c

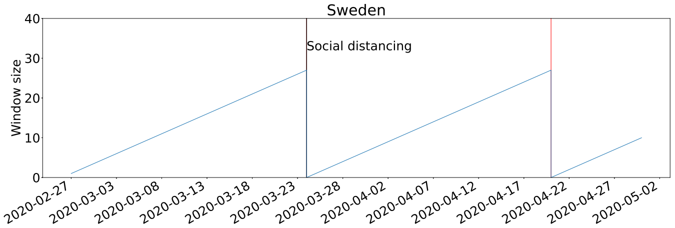

d

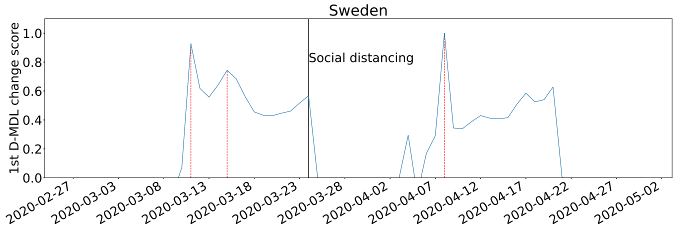

e

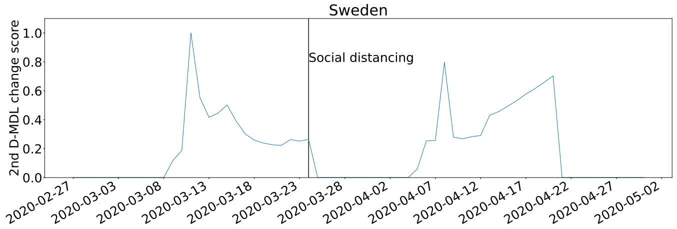

Fig. 64: The results for Sweden with exponential modeling. The date on which the social distancing was implemented is marked by a solid line in black. **a,** the number of cumulative cases. **b,** the change scores produced by the 0th M-DML where the line in blue denotes values of scores and dashed lines in red mark alarms. **c,** the window sized for the sequential D-DML algorithm with adaptive window where lines in red mark the shrinkage of windows. **d,** the change scores produced by the 1st D-MDL. **e,** the change scores produced by the 2nd D-MDL.

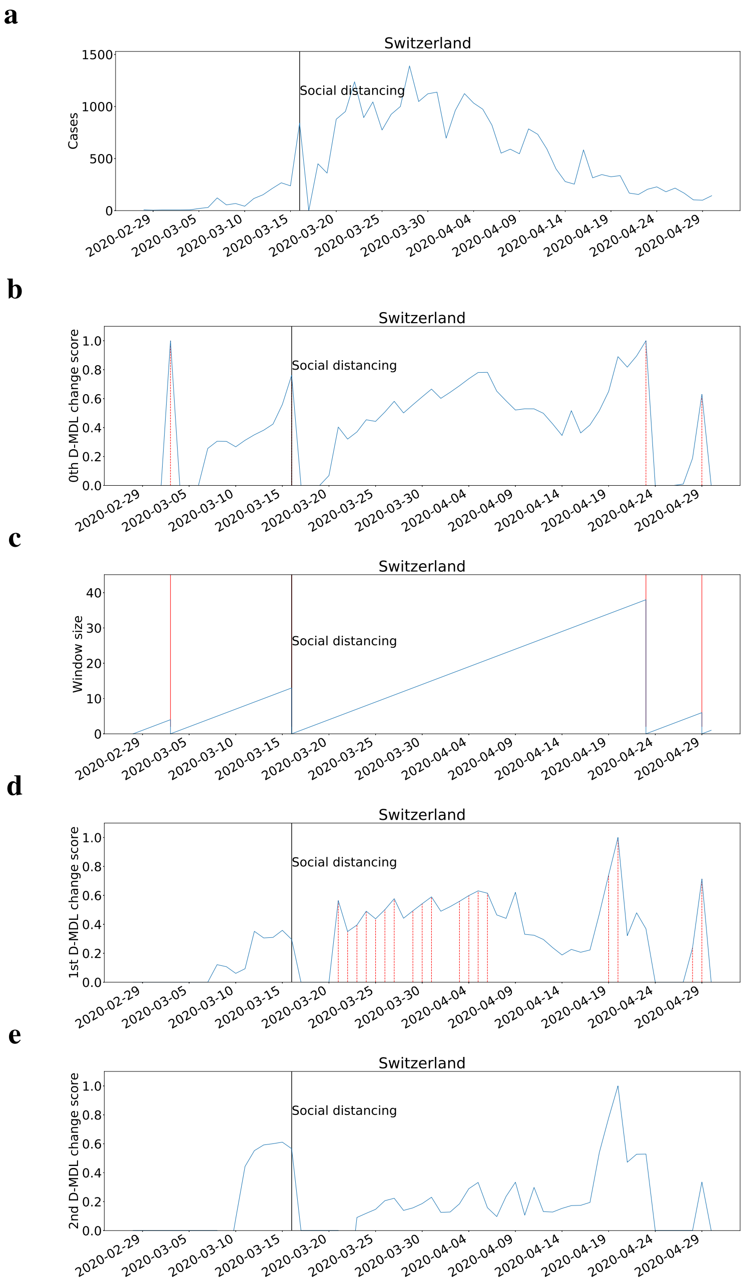

Fig. 65: The results for Switzerland with Gaussian modeling. The date on which the social distancing was implemented is marked by a solid line in black. **a,** the number of daily new cases. **b,** the change scores produced by the 0th M-DML where the line in blue denotes values of scores and dashed lines in red mark alarms. **c,** the window sized for the sequential D-DML algorithm with adaptive window where lines in red mark the shrinkage of windows. **d,** the change scores produced by the 1st D-MDL. **e,** the change scores produced by the 2nd D-MDL.

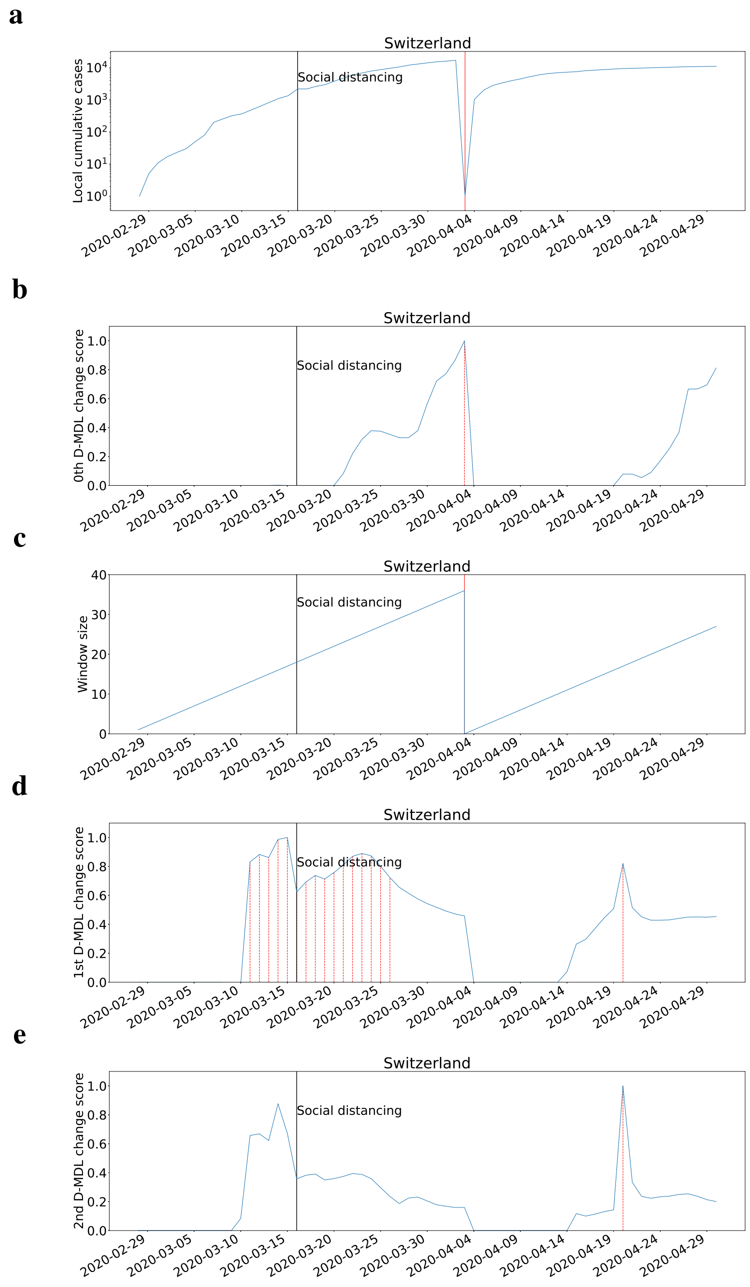

Fig. 66: The results for Switzerland with exponential modeling. The date on which the social distancing was implemented is marked by a solid line in black. **a,** the number of cumulative cases. **b,** the change scores produced by the 0th M-DML where the line in blue denotes values of scores and dashed lines in red mark alarms. **c,** the window sized for the sequential D-DML algorithm with adaptive window where lines in red mark the shrinkage of windows. **d,** the change scores produced by the 1st D-MDL. **e,** the change scores produced by the 2nd D-MDL.

a

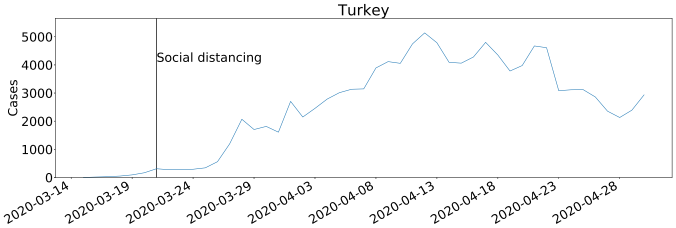

b

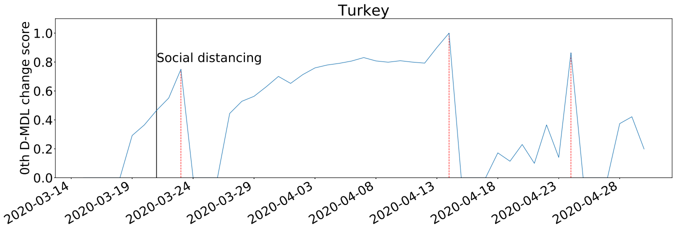

c

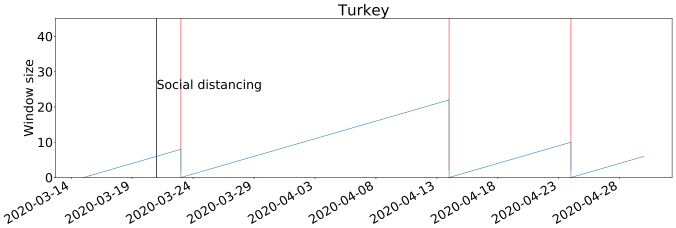

d

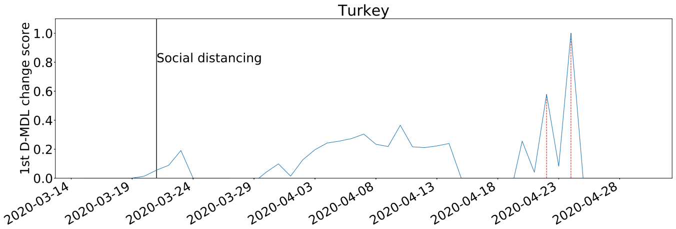

e

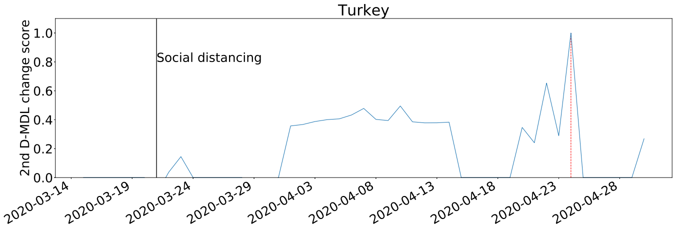

Fig. 67: The results for Turkey with Gaussian modeling. The date on which the social distancing was implemented is marked by a solid line in black. **a,** the number of daily new cases. **b,** the change scores produced by the 0th M-DML where the line in blue denotes values of scores and dashed lines in red mark alarms. **c,** the window sized for the sequential D-DML algorithm with adaptive window where lines in red mark the shrinkage of windows. **d,** the change scores produced by the 1st D-MDL. **e,** the change scores produced by the 2nd D-MDL.

a

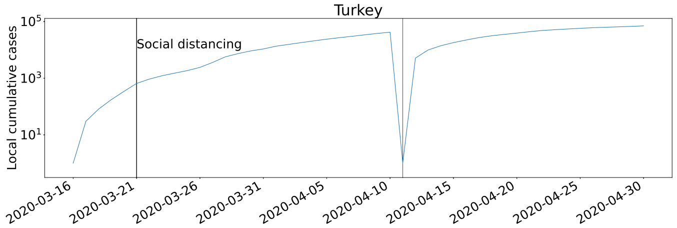

b

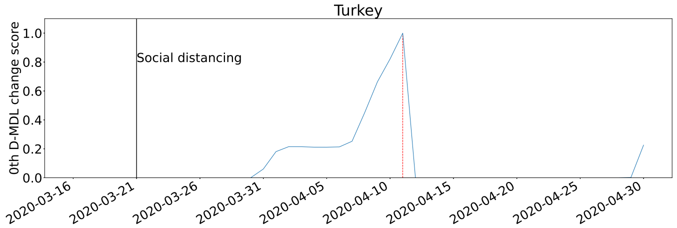

c

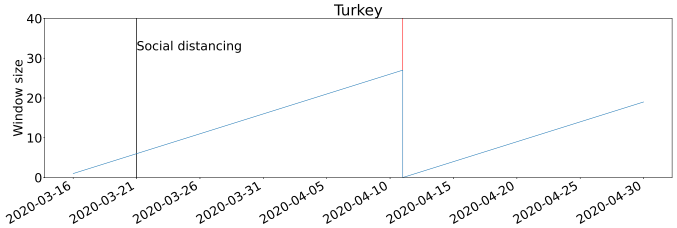

d

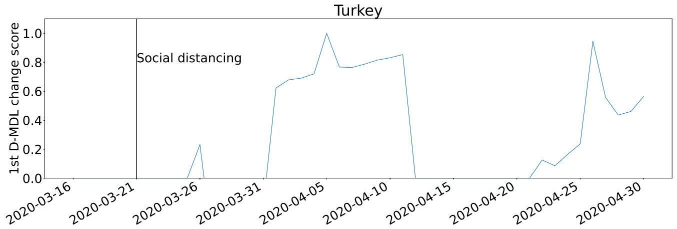

e

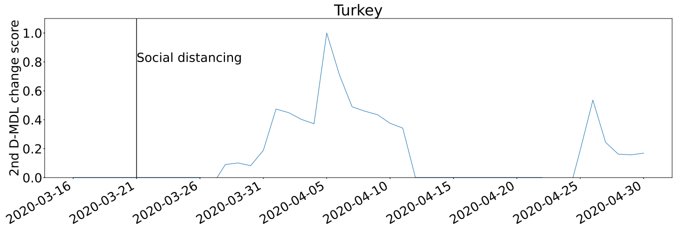

Fig. 68: The results for Turkey with exponential modeling. The date on which the social distancing was implemented is marked by a solid line in black. **a,** the number of cumulative cases. **b,** the change scores produced by the 0th M-DML where the line in blue denotes values of scores and dashed lines in red mark alarms. **c,** the window sized for the sequential D-DML algorithm with adaptive window where lines in red mark the shrinkage of windows. **d,** the change scores produced by the 1st D-MDL. **e,** the change scores produced by the 2nd D-MDL.

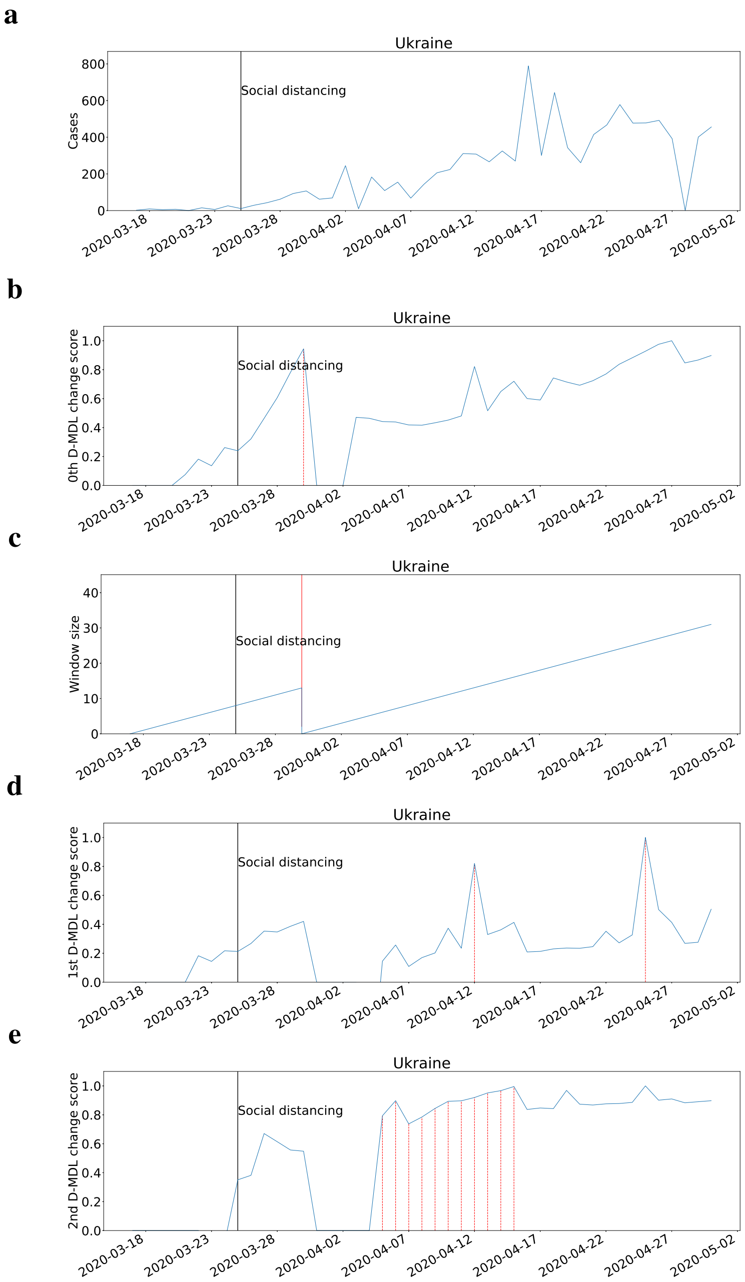

Fig. 69: The results for Ukraine with Gaussian modeling. The date on which the social distancing was implemented is marked by a solid line in black. **a,** the number of daily new cases. **b,** the change scores produced by the 0th M-DML where the line in blue denotes values of scores and dashed lines in red mark alarms. **c,** the window sized for the sequential D-DML algorithm with adaptive window where lines in red mark the shrinkage of windows. **d,** the change scores produced by the 1st D-MDL. **e,** the change scores produced by the 2nd D-MDL.

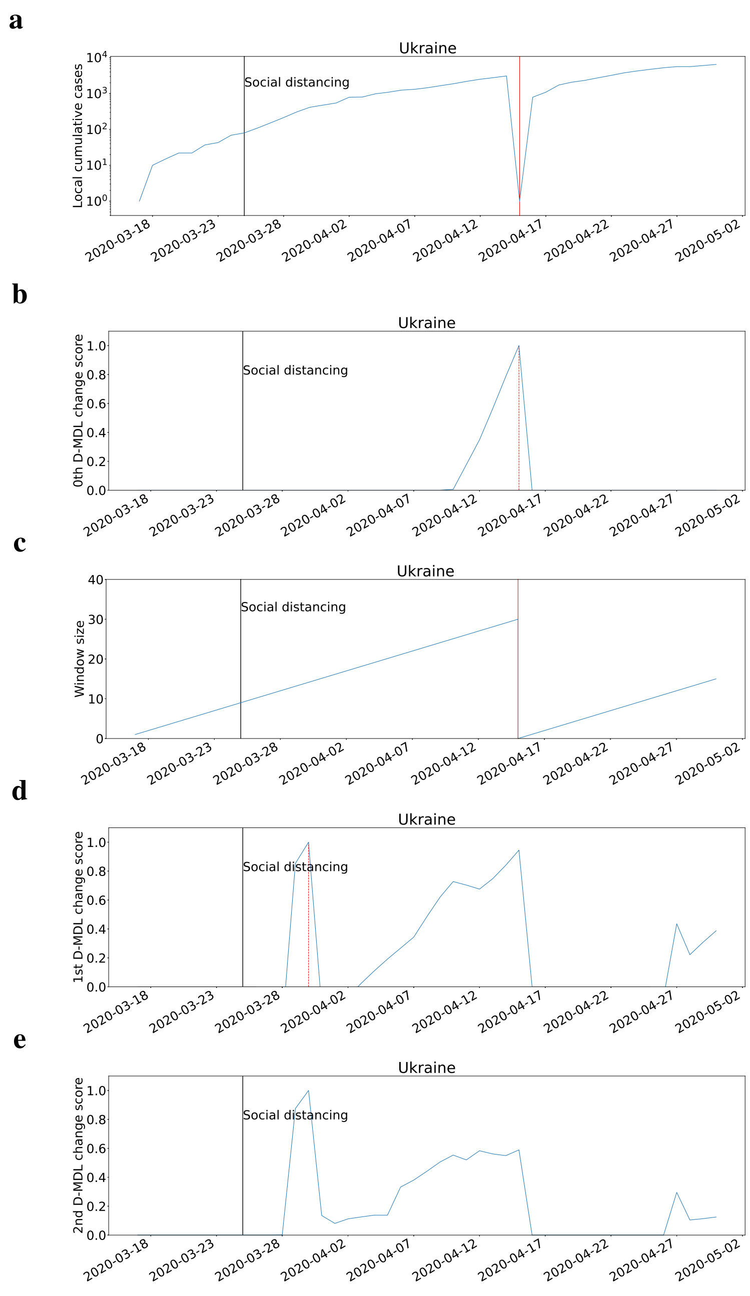

Fig. 70: The results for Ukraine with exponential modeling. The date on which the social distancing was implemented is marked by a solid line in black. **a,** the number of cumulative cases. **b,** the change scores produced by the 0th M-DML where the line in blue denotes values of scores and dashed lines in red mark alarms. **c,** the window sized for the sequential D-DML algorithm with adaptive window where lines in red mark the shrinkage of windows. **d,** the change scores produced by the 1st D-MDL. **e,** the change scores produced by the 2nd D-MDL.

a

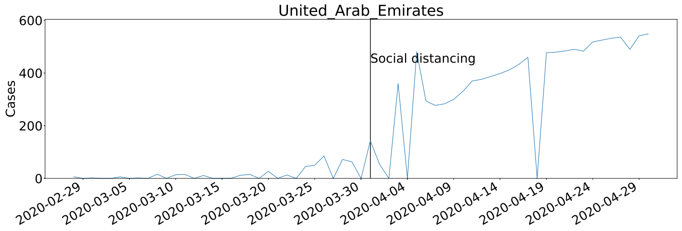

b

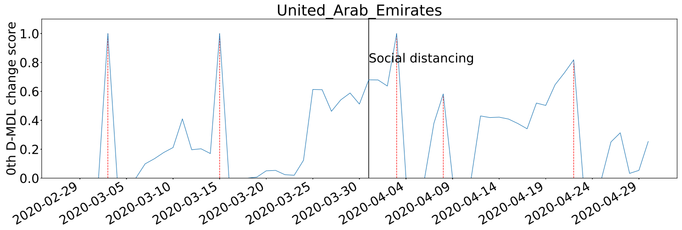

c

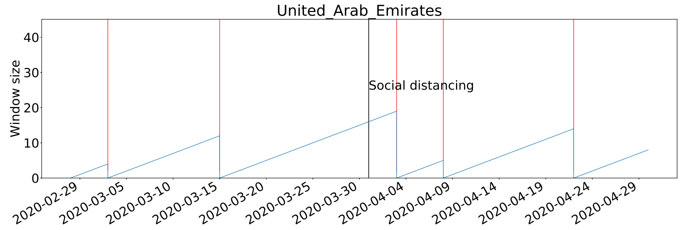

d

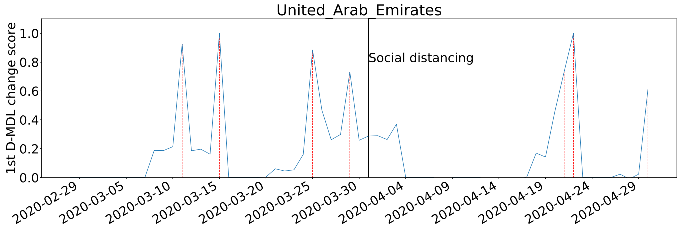

e

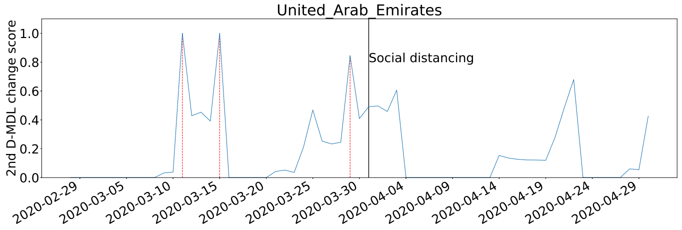

Fig. 71: The results for the United Arab Emirates with Gaussian modeling. The date on which the social distancing was implemented is marked by a solid line in black. **a,** the number of daily new cases. **b,** the change scores produced by the 0th M-DML where the line in blue denotes values of scores and dashed lines in red mark alarms. **c,** the window sized for the sequential D-DML algorithm with adaptive window where lines in red mark the shrinkage of windows. **d,** the change scores produced by the 1st D-MDL. **e,** the change scores produced by the 2nd D-MDL.

a

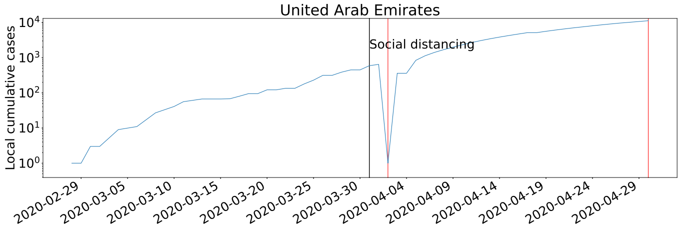

b

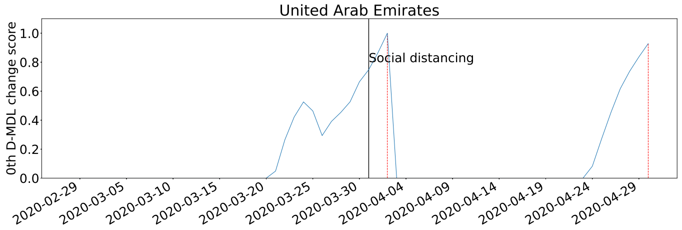

c

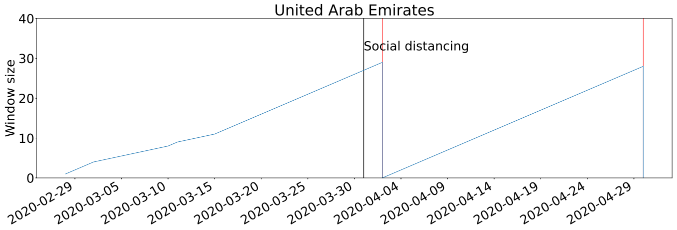

d

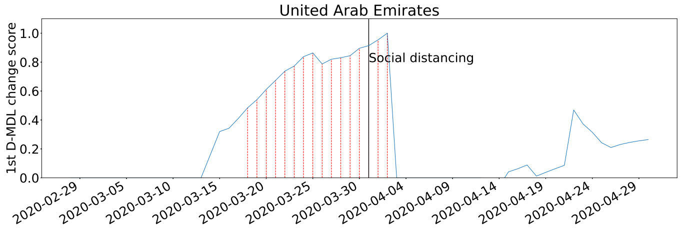

e

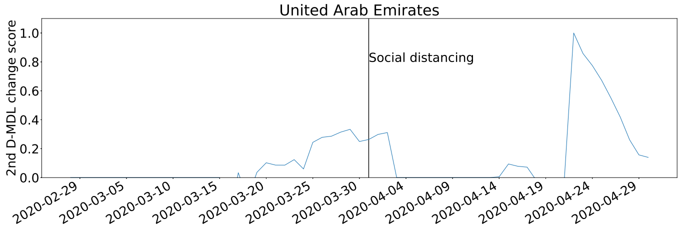

Fig. 72: The results for United Arab Emirates with exponential modeling. The date on which the social distancing was implemented is marked by a solid line in black. **a,** the number of cumulative cases. **b,** the change scores produced by the 0th M-DML where the line in blue denotes values of scores and dashed lines in red mark alarms. **c,** the window sized for the sequential D-DML algorithm with adaptive window where lines in red mark the shrinkage of windows. **d,** the change scores produced by the 1st D-MDL. **e,** the change scores produced by the 2nd D-MDL.

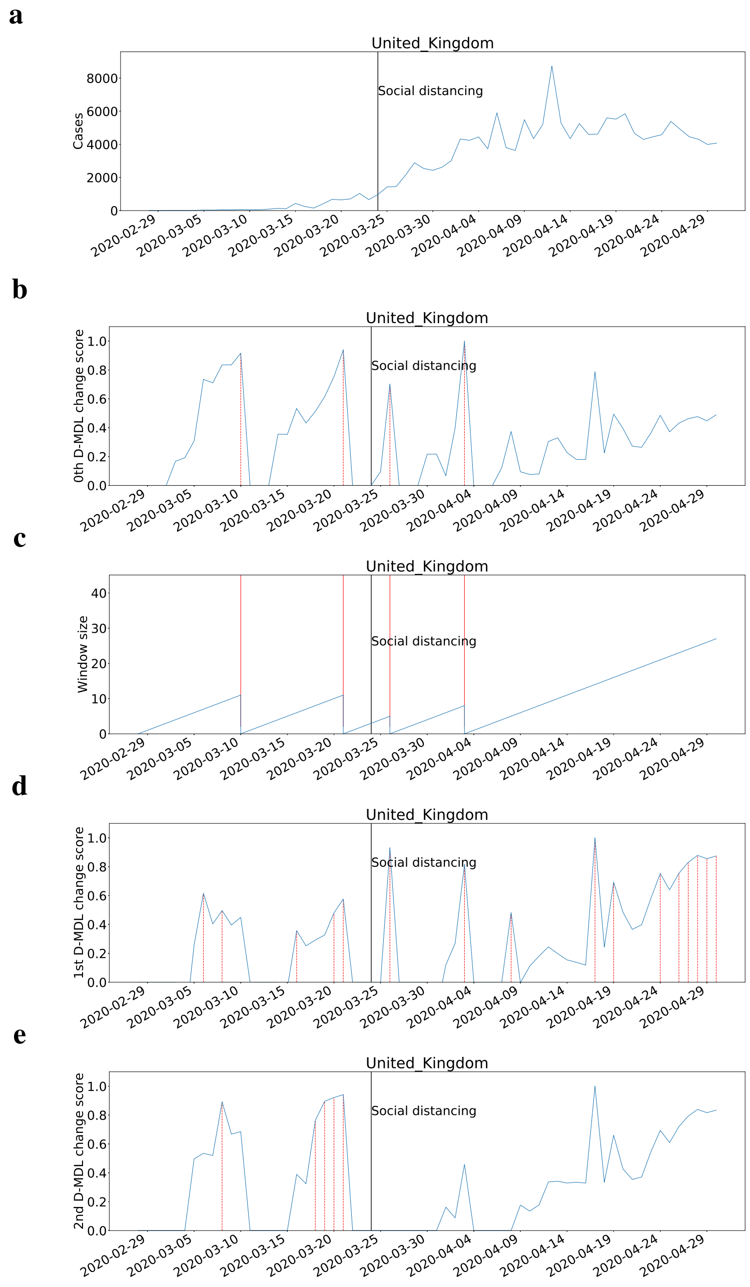

Fig. 73: The results for the United Kingdom with Gaussian modeling. The date on which the social distancing was implemented is marked by a solid line in black. **a,** the number of daily new cases. **b,** the change scores produced by the 0th M-DML where the line in blue denotes values of scores and dashed lines in red mark alarms. **c,** the window sized for the sequential D-DML algorithm with adaptive window where lines in red mark the shrinkage of windows. **d,** the change scores produced by the 1st D-MDL. **e,** the change scores produced by the 2nd D-MDL.

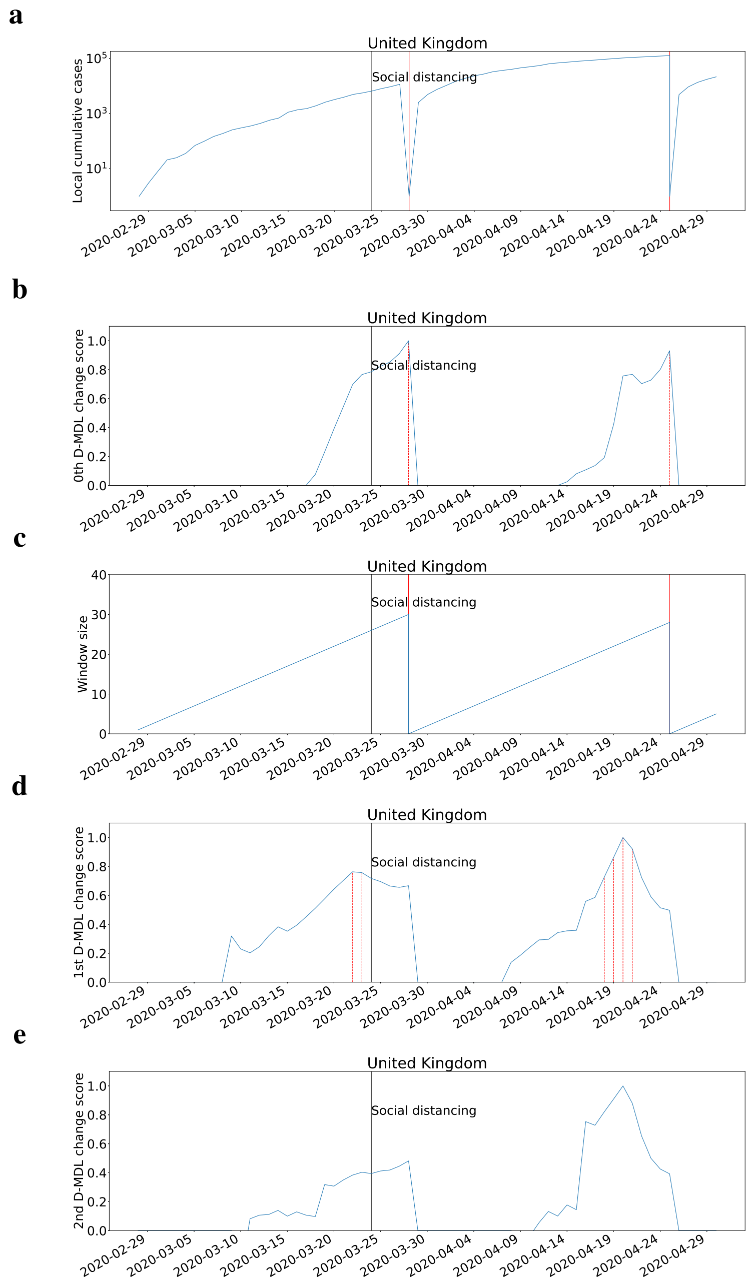

Fig. 74: The results for United Kingdom with exponential modeling. The date on which the social distancing was implemented is marked by a solid line in black. **a,** the number of cumulative cases. **b,** the change scores produced by the 0th M-DML where the line in blue denotes values of scores and dashed lines in red mark alarms. **c,** the window sized for the sequential D-DML algorithm with adaptive window where lines in red mark the shrinkage of windows. **d,** the change scores produced by the 1st D-MDL. **e,** the change scores produced by the 2nd D-MDL.

a

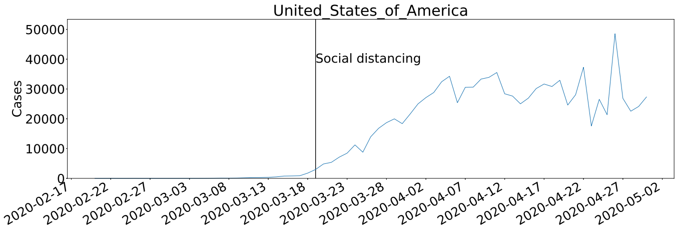

b

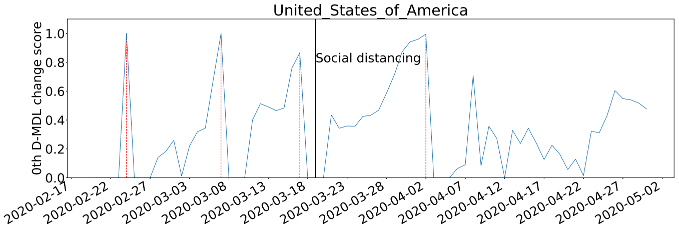

c

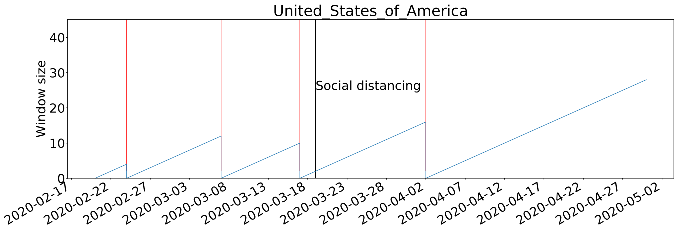

d

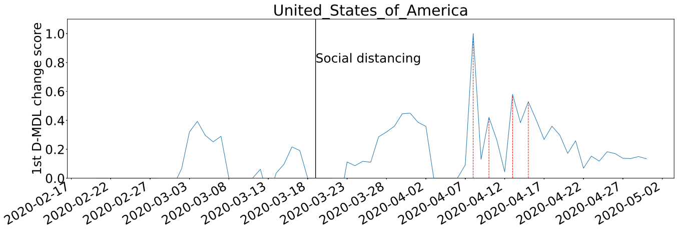

e

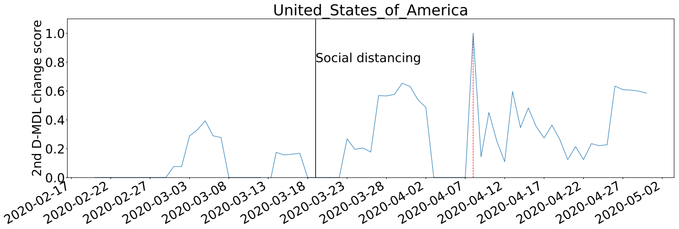

Fig. 75: The results for the United States of America with Gaussian modeling. The date on which the social distancing was implemented is marked by a solid line in black. **a,** the number of daily new cases. **b,** the change scores produced by the 0th M-DML where the line in blue denotes values of scores and dashed lines in red mark alarms. **c,** the window sized for the sequential D-DML algorithm with adaptive window where lines in red mark the shrinkage of windows. **d,** the change scores produced by the 1st D-MDL. **e,** the change scores produced by the 2nd D-MDL.

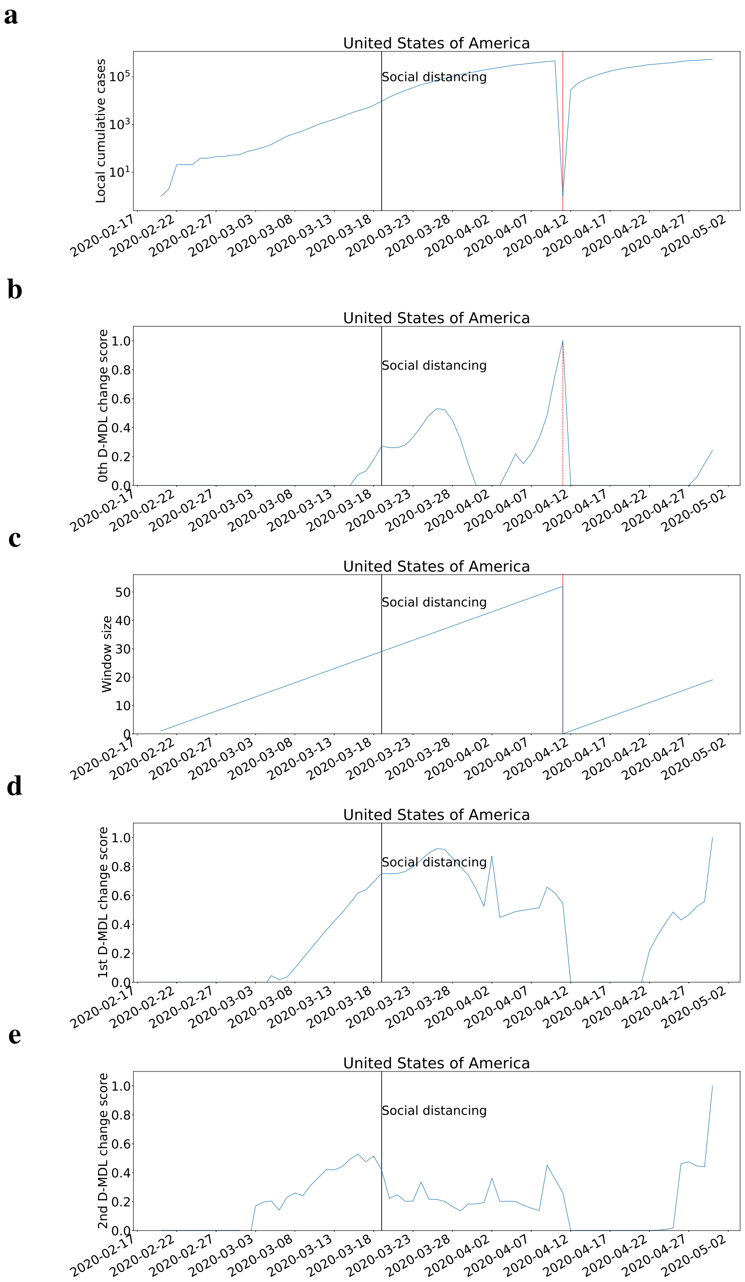

Fig. 76: The results for the United States of America with exponential modeling. The date on which the social distancing was implemented is marked by a solid line in black. **a,** the number of cumulative cases. **b,** the change scores produced by the 0th M-DML where the line in blue denotes values of scores and dashed lines in red mark alarms. **c,** the window sized for the sequential D-DML algorithm with adaptive window where lines in red mark the shrinkage of windows. **d,** the change scores produced by the 1st D-MDL. **e,** the change scores produced by the 2nd D-MDL.

References

[1] Rissanen, J.: MDL denoising, *IEEE Transactions on Information Theory*,46(7), pp:2537–2543 (2000).

[2] Shim, E., Tariq, A., Choi, W., Lee, Y., & Chowell, G. Transmission potential and severity of COVID-19 in South Korea. *Int. J. Infect. Dis.* **93**, 339-344 (2020).

[3] Park, S.W., Sun, K., Viboud, C., Grenfell, B.T. & Dushoff, J. Potential roles of social distancing in mitigating the spread of coronavirus disease 2019 (COVID-19) in South Korea. Preprint at `https://www.medrxiv.org/content/10.1101/2020.03.27.20045815v1` (2020).

[4] Greenstone, M. & Nigam, V. Does social distancing matter? *University of Chicago, Becker Friedman Institute for Economics Working Paper*, `https://bfi.uchicago.edu/wp-content/uploads/BFI$\_$WP$\_$202026.pdf` (2020).

[5] Wilder-Smith, A. & Freedman, D.O. Isolation, quarantine, social distancing and community containment: pivotal role for old-style public health measures in the novel coronavirus (2019-nCoV) outbreak. *J. Travel Med.* **27(2)**, doi: `https://doi.org/10.1093/jtm/taaa020` (2020).

[6] Jones, J. H. Notes on R0. *California: Department of Anthropological Sciences*, `https://web.stanford.edu/~jhj1/teachingdocs/Jones-on-R0.pdf` (2007).
